# αIIbβ3 variants in ten families with autosomal dominant macrothrombocytopenia: Expanding the mutational and clinical spectrum

**DOI:** 10.1371/journal.pone.0235136

**Published:** 2020-12-04

**Authors:** Sara Morais, Jorge Oliveira, Catarina Lau, Mónica Pereira, Marta Gonçalves, Catarina Monteiro, Ana Rita Gonçalves, Rui Matos, Marco Sampaio, Eugénia Cruz, Inês Freitas, Rosário Santos, Margarida Lima

**Affiliations:** 1 Setor de Trombose e Hemostase, Serviço de Hematologia Clínica, Hospital de Santo António (HSA), Centro Hospitalar Universitário do Porto (CHUP), Porto, Portugal; 2 Unidade Multidisciplinar de Investigação Biomédica, Instituto de Ciências Biomédicas, Universidade do Porto (UMIB/ICBAS/UP), Porto, Portugal; 3 Unidade de Genética Molecular, Centro de Genética Médica Doutor Jacinto Magalhães (CGMJM), Centro Hospitalar Universitário do Porto (CHUP), Porto, Portugal; 4 Laboratório de Citometria, Unidade de Diagnóstico Hematológico, Serviço de Hematologia Clínica, Hospital de Santo António (HSA), Centro Hospitalar Universitário do Porto (CHUP), Porto, Portugal; 5 Serviço de Hematologia Laboratorial, Hospital de Santo António (HSA), Centro Hospitalar Universitário do Porto (CHUP), Porto, Portugal; Universite de Liege (B34), BELGIUM

## Abstract

**Background:**

Rare pathogenic variants in either the *ITGA2B* or *ITGB3* genes have been linked to autosomal dominant macrothrombocytopenia associated with abnormal platelet production and function, deserving the designation of Glanzmann Thrombasthenia-Like Syndrome (GTLS) or ITGA2B/ITGB3-related thrombocytopenia.

**Objectives:**

To describe a series of patients with familial macrothrombocytopenia and decreased expression of αIIbβ3 integrin due to defects in the *ITGA2B* or *ITGB3* genes.

**Methods:**

We reviewed the clinical and laboratory records of 10 Portuguese families with GTLS (33 patients and 11 unaffected relatives), including the functional and genetic defects.

**Results:**

Patients had absent to moderate bleeding, macrothrombocytopenia, low αIIbβ3 expression, impaired platelet aggregation/ATP release to physiological agonists and low expression of activation-induced binding sites on αIIbβ3 (PAC-1) and receptor-induced binding sites on its ligand (bound fibrinogen), upon stimulation with TRAP-6 and ADP. Evidence for constitutive αIIbβ3 activation, occurred in 2 out of 9 patients from 8 families studied, but also in 2 out of 12 healthy controls. We identified 7 missense variants: 3 in *ITGA2B* (5 families), and 4 in *ITGB3* (5 families). Three variants (αIIb: p.Arg1026Trp and p.Arg1026Gln and β3: p.Asp749His) were previously reported. The remaining (αIIb: p.Gly1007Val and β3: p.Thr746Pro, p.His748Pro and p.Arg760Cys) are new, expanding the αIIbβ3 defects associated with GTLS. The integration of the clinical and laboratory data allowed the identification of two GTLS subgroups, with distinct disease severity.

**Conclusions:**

Previously reported *ITGA2B* and *ITGB3* variants related to thrombocytopenia were clustered in a confined region of the membrane-proximal cytoplasmic domains, the inner membrane clasp. For the first time, variants are reported at the outer membrane clasp, at the transmembrane domain of αIIb, and at the membrane distal cytoplasmic domains of β3. This is the largest single-center series of inherited macrothrombocytopenia associated with αIIbβ3 variants published to date.

## Introduction

Integrins are membrane proteins composed of alpha (α) and beta (β) subunits, each comprising a large extracellular domain (ECD) that binds to extracellular matrix (ECM) components, a single transmembrane (TM) domain (TMD), and a short C-terminal cytosolic domain (CTD) that interacts with the cytoskeleton and signaling molecules [1]. Integrins expressed on the cell surface are normally in an inactive state resulting from two major interactions: the “inner membrane clasp” (IMC) and the “outer membrane clasp” (OMC), which, in the case of integrin αIIbβ3, are based on the hydrophobic αIIb residues and αIIb–β3 salt bridge on the membrane-cytoplasmatic domains of the α- and β-subunits, and hydrophobic packing of TM residues on the extracellular side between the α- and β-subunits, respectively [2,3].

Integrin activation comprises outside-in and inside-out signalling, conformational changes in the heterodimer, and integrin clustering [4]. Inside-out activation starts with a signal generated inside the cell and leading to an increased affinity of the integrin ECD for its ligand. In outside-in activation, ligand binding induces conformational changes and integrin clustering, with subsequent intracellular events, such as phosphorylation. Talin, a protein that forms a linkage between the integrins and the actin cytoskeleton, binds the membrane-proximal and -distal regions of integrin cytoplasmic-TMDs, loosen the IMC and causing tail separation and conformational changes in the ECD [3,5].

Platelets (PLT), which originate from proplatelets formed by a complex demarcation of the megakaryocyte membrane [6,7], contain three β1 integrins that mediate PLT adhesion to the matrix proteins, and two β3 integrins, αvβ3 and αIIbβ3. Expression of integrin αIIbβ3, also named glycoprotein (GP) IIb/IIIa receptor complex, is restricted to the megakaryocyte lineage, being the most abundant PLT receptor [8].

Under normal conditions, αIIbβ3 is inactive, exhibiting low affinity for its primary ligand, fibrinogen (FG). Upon PLT activation, inside-out signaling promotes the association of αIIb and β3 to form the activated αIIbβ3 receptor complex, increasing its affinity for FG; integrin clustering then stimulates outside-in signaling, amplifying PLT activation [9]. Previous observations have suggested that αIIbβ3 is also involved in proplatelet formation and release of PLTs from proplatelet tips [10,11].

Pathogenic variants in *ITGA2B* and *ITGB3*, the genes coding for the αIIb and β3, give rise to Glanzmann´s Thrombasthenia (GT), a rare autosomal recessive (AR) bleeding disorder due to quantitative or qualitative defects of integrin αIIbβ3, characterized by absent PLT aggregation, and normal PLT counts and volumes [12]. Until March 2019, approximately 440 pathogenic variants were listed in the GT database (https://glanzmann.mcw.edu/) and seen to be widely distributed across both genes [13].

A limited number of patients have been reported to have autosomal dominant (AD) variants of GT associated with macrothrombocytopenia, PLT anisocytosis, PLT function defects and mild to moderate hemorrhage. These cases have deserved the designation of GT-like syndrome (GTLS) or ITGA2B/ITGB3-related thrombocytopenia (ITGA2B/ITGB3-RT). The first description dates to the 1990s [14,15], and, along the recent years, the disease has been characterized, with only a few variants being described in the *ITGA2B* and *ITGB3* genes. Genetic defects identified in GTLS patients localize mostly in TM-cytoplasmic domains of αIIbβ3, and some of these variants appear to cause constitutive activation of the GPIIb/IIIa receptor and to impair proplatelet formation [14–23]. None of them was identified in populational databases, such as ExAC/gnomAD, suggesting that they are very rare, with a minor allele frequency (MAF) < 0.01% [23].

In 1995, we identified for the first time a family with moderate bleeding tendency, low PLT counts, increased PLT size, and decreased expression of αIIbβ3 on the PLT surface, and at the time a single case had been described in the literature with comparable characteristics [14]. This family was subsequently found to have a heterozygous variant in *ITGA2B* (p.Arg1026Trp), in common with a case published in 2011 [19]. In 2006, we reported this family and three additional families [24]. We have since found 14 unrelated Portuguese families (42 patients) with macrothrombocytopenia and low αIIbβ3 expression. This is presently the most frequent inherited thrombocytopenia in our center, as opposed to other series reported in the literature [25–27]. Altogether, GTLS represents about 17% of families with inherited thrombocytopenia with an identified genetic defect being followed up in our hospital, being more frequent than *TUBB1* (13%), *ACTN1* (10%), *ANKRD26* (11%), and *MYH9* (10%) related thrombocytopenia, and biallelic (7%) or monoallelic (6%) Bernard Soulier Syndrome.

We report on 33 patients from 10 families with genetically characterized GTLS, describing their clinical manifestations and laboratory features, including the underlying functional and genetic defects, and discussing their contribution to disease manifestations. This series is the largest cohort of familial macrothrombocytopenia and decreased expression of αIIbβ3 described by a single center. It includes 3 previously reported and 4 novel missense variants, widening the genotypic and phenotypic spectrum of this rare disease.

## Material and methods

### Study population

Patients and their relatives (studied for segregation evaluation) belong to families with thrombocytopenia and decreased expression of integrin αIIbβ3, followed at the Centro Hospitalar Universitário do Porto (CHUP), Portugal. Healthy adult individuals used as controls were blood donors.

### Ethics approval and consent to publish

The study was approved by the Hospital Ethics Committee and Hospital Research Coordination Office, and authorized by the Hospital Administration Board, being registered with the number 2016/167-141-DEFI/130-CES. All patients and control subjects gave the informed consent before prior to entering the study, and the procedures followed were in accordance with the Helsinki Declaration.

### Clinical evaluation

Patients and their relatives underwent a clinical appointment where the bleeding score (BS) was determined using the International Society on Thrombosis and Hemostasis (ISTH)–Bleeding Assessment Tool (BAT) (ISTH-BAT) [28,29], and their familial history was collected. They were asked to cease all medication that could potentially interfere with PLT function for at least one week before blood collection.

Before blood donation, blood donors were interviewed by a medical doctor, being submitted to a brief clinical assessment to ensure that they were in good health, did not take any anticoagulant or anti-platelet drugs and did not have a history of bleeding or thrombosis.

### Blood samples

Peripheral blood (PB) samples were collected by venipuncture into vacutainer tubes containing sodium citrate for PLT lumiaggregometry and flow cytometry (FCM), and ethylene diamine tetracetic acid (EDTA)-K3 for PLT counts and to perform genetic studies. The first 3–4 ml of blood were discarded. The samples were processed within 2 hours after collection and stored at room temperature until use.

Platelet-rich plasma (PRP) used for PLT aggregometry studies was obtained from citrated whole blood from the patients and from at least three donors (used to prepare a pool of normal PRP) by centrifuging for 15 minutes at 150 *g*. Platelet poor plasma (PPP) was then obtained by centrifuging the remaining cell pellet for a further 15 minutes at 1500 *g*.

### Platelet counts and platelet indexes

The PLT count (normal range 150-400x10^9^/L) and the PLT indexes, which included the mean platelet volume (MPV) (normal range: 7.2–11.1fL), the platelet distribution width (PDW) (normal range: 9–14%) and the immature platelet fraction (IPF) (normal range: 1–7%), were determined using automated hematological analyzers (ADVIA 2120 –Siemens, Eschborn, Germany, or Sysmex XE-2100 –TOA Medical Electronics, Kobe, Japan).

### Platelet morphology

Platelet morphology was evaluated in blood smears stained with Leishman's solution (Merck, Darmstadt, Germany), and observed under light microscopy.

### Platelet functional studies

Automated assessment for PLT function included occlusion tests in citrated whole blood samples using a Platelet Function Analyzer (PFA) (PFA-100 and INNOVANCE PFA-200® System for the more recent samples; Siemens, Marburg, Germany) and employing test cartridges of both collagen/epinephrine (COL/EPI) (normal range: 88–146 seconds) and collagen/adenosine diphosphate (COL/ADP) (normal range: 56–120 seconds).

Light transmission aggregometry and adenosine triphosphate (ATP) release (ATP-R) were evaluated using a Chrono-Log 700 series or a 560CA optical lumiaggregometer (Chrono-Log Corporation, Havertown, PA, USA) (CLC) in the undiluted PRP and using the PPP as a baseline. Platelet aggregation studies were performed in PRP and the results are given as percentage maximum aggregation. ATP release was measured using the luciferin luciferase system (Chrono-Lume, CLC); the equipment was calibrated according to the manufacturer´s instructions. The changes in light transmission and luminescence were recorded for at least 300 seconds.

The following agonists were used at the indicated final concentrations: adenosine diphosphate (ADP) (CLC, PN-384; 10 μM), epinephrine (EPI) (CLC, PN-393; 10 μM), arachidonic acid (AA) (CLC, PN-390; 1mM), collagen (COL) (CLC, PN-385; 1.0 μg/mL), ristocetin (RIST) (CLC, PN-396; 0.5 and 1 mM), and Thrombin Receptor Agonist Peptide type 6 (TRAP-6) (Stago, France, 86926; 25 μM).

### Flow cytometry assays

#### Analysis of platelet glycoproteins

Surface expression of CD41a (GPIIb/IIIa), CD61 (GPIIIa) and CD42b (GPIb) was measured in citrated whole blood by FCM, using an in-house developed semi-quantitative method (S1 File, http://doi.org/10.5281/zenodo.3660449). Samples were analyzed in a Navios™ flow cytometer (Beckman Coulter–BC, Hialeah, FL, USA). PLTs were gated based on their forward (FSC) and side (SSC) scatter, and expression of the PLT-associated GP, and the median fluorescence intensity (MFI) was measured for each platelet GP analyzed. The results were expressed as a percentage of normal (patient MFI/median MFI of controls*100) and corrected for PLT size, as evaluated by the FSC. Normal reference values are: CD41a (85–115%); CD61 (80–120%); CD42b (70–130%).

#### Platelet activation studies

Platelet activation was assessed in whole blood using an in-house developed method, one hour after blood collection, in resting (basal, BAS), in inhibitory (with EDTA, 7 mM) and in stimulatory conditions, the latter being induced by PLT agonists: ADP (10 μM) or TRAP-6 (20 μM) (S1 File, http://doi.org/10.5281/zenodo.3660449).

Monoclonal antibodies (mAb) used were phycoerythrin (PE)-conjugated mouse anti-CD42b (GPIb) (clone AN51; Dako, Glostrup, Denmark) (DK), for gating the PLT cell population, and fluorescein isothiocyanate (FITC)-conjugated mouse anti-human PAC-1 (clone PAC-1, Becton Dickinson–BD, San Jose, CA, USA) and anti-human bound fibrinogen (bFG) (clone 9F9; Biocytex, Marseille, France) to measure PLT activation.

All samples were analyzed in a Navios™ flow cytometer (BC). PLTs were gated based on their FSC and SSC and cell surface expression of CD42b, and the MFI was measured for each activation-related marker analyzed (PAC-1 and bFG).

Platelet activation was measured by calculating the Platelet Activation Index (PAI) expressed as fold increase in the MFI obtained for a given activation-related marker (PAC-1 or bFG) in the test tubes (i.e. basal conditions and stimulatory conditions with ADP or TRAP-6), comparatively to the MFI obtained in the corresponding inhibitory conditions (i.e. in the presence of EDTA): (MFI test tube—MFI inhibitory tube) / MFI inhibitory tube. Also, the expression of PAC-1 or bFG in patients´ PLTs was compared to that observed in healthy controls, by calculating the ratio between the MFI obtained in each patient and the mean MFI obtained in the healthy controls processed under the same conditions, expressed as a percentage of the normal values (MFI of patient / mean MFI of controls*100).

One patient was studied per family, except for F3 and F10 (not studied) and F5 (2 patients studied), totalizing 9 patients from 8 families. Two normal blood samples obtained from healthy individuals were run daily, in parallel with patient samples, and two additional normal PB samples were studied, totalizing 12 normal PB samples.

### Genetic studies

After extraction of deoxyribonucleic acid (DNA) from whole blood, genetic analysis of *ITGA2B* and *ITGB3* genes was performed by next-generation sequencing (NGS) in selected patients from families 1, 4–6 and 8. Briefly, NGS libraries were prepared using an amplicon-based approach, resorting to a commercially available gene panel for hematologic diseases (Ion Ampliseq, Thermo Fisher Scientific–TFS, Waltham, Massachusetts, U.S.A) (**S2 File**). Template preparation and sequencing were performed on an Ion Chef and an Ion S5 system, respectively, according to the manufacturer’s instructions (TFS). Data analysis was conducted using the Ion Reporter (TFS) and Alamut Visual Software (Interactive Biosoftware, Rouen, France). Upon the identification of variants c.3020G>T, c.3076C>T and c.3077G>A (*ITGA2B* gene) and c.2236A>C, c.2245G>C and c.2278C>T (*ITGB3* gene), these were confirmed by targeted Sanger sequencing (primer sequences in **S2 File**).

The same approach was used to screen these variants in the remaining families and, in patients that remained uncharacterized, Sanger sequencing was carried out on the entire coding and flanking intronic regions of the *ITGA2B* and *ITGB3* genes. After purification, amplicons were sequenced using BigDye kit V3.1. Sequencing electropherograms were analysed using SeqScape v2.5 (TFS).

Variants are described using reference sequences NM_000419.4; NP_000410.2 for *ITGA2B* and NM_000212.2 / NP_000203.2 for *ITGB3*. In terms of legacy, the amino acid numbers may differ from those in previous publications, where numbering did not consider the signal peptide sequences (31 residues for αIIb and 26 residues for β3).

### Statistics

Qualitative data are presented as absolute and relative frequencies. Quantitative data are expressed as median, minimum and maximum values, and mean ± standard deviation (SD). Significance of between-group differences was tested by the non-parametric Mann-Whitney test for continuous variables and the chi-square test for categorical variables. The Spearman's rank correlation test was used to assess a possible two-way linear association between two continuous variables, the correlation being measured by the Spearman's rank correlation coefficient (R). P values <0.05 were considered statistically significant. Statistics were performed using SOFA Statistics version 1.4.6 (Paton-Simpson & Associates Ltd, Auckland, New Zealand).

## Results and discussion

**Fig 1:**
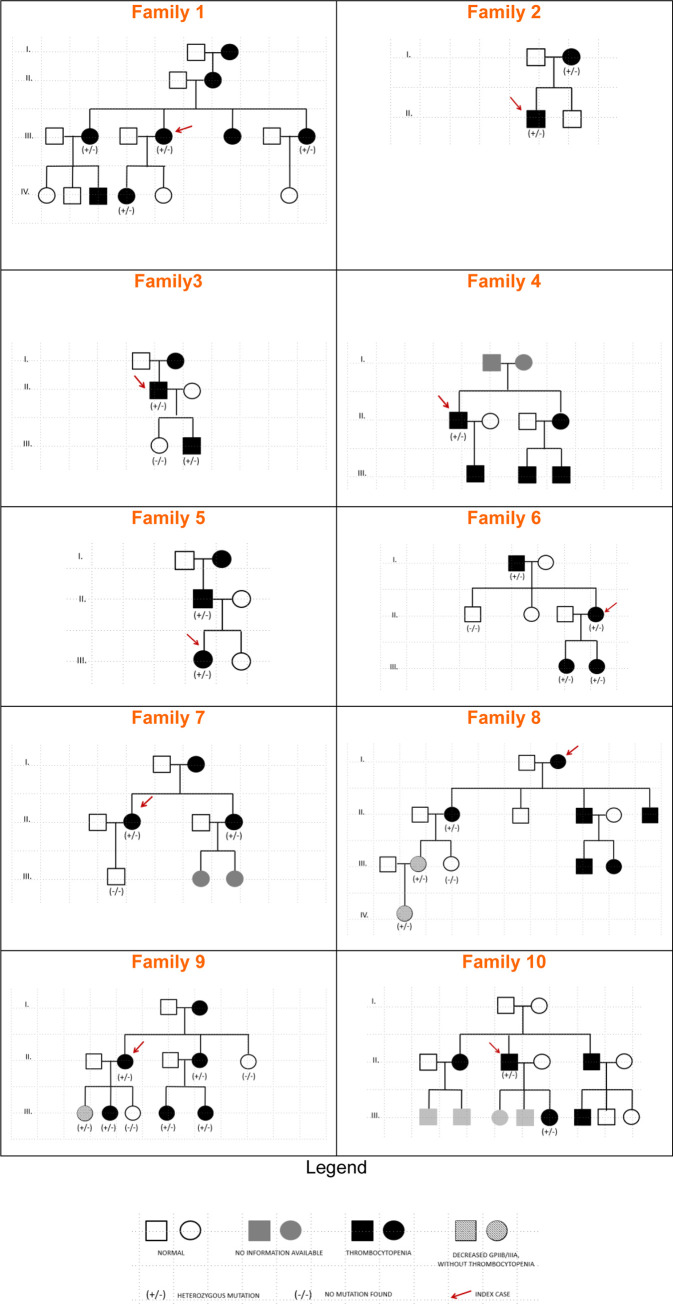
Pedigrees of the GTLS families described in this study.

**Fig 2:**
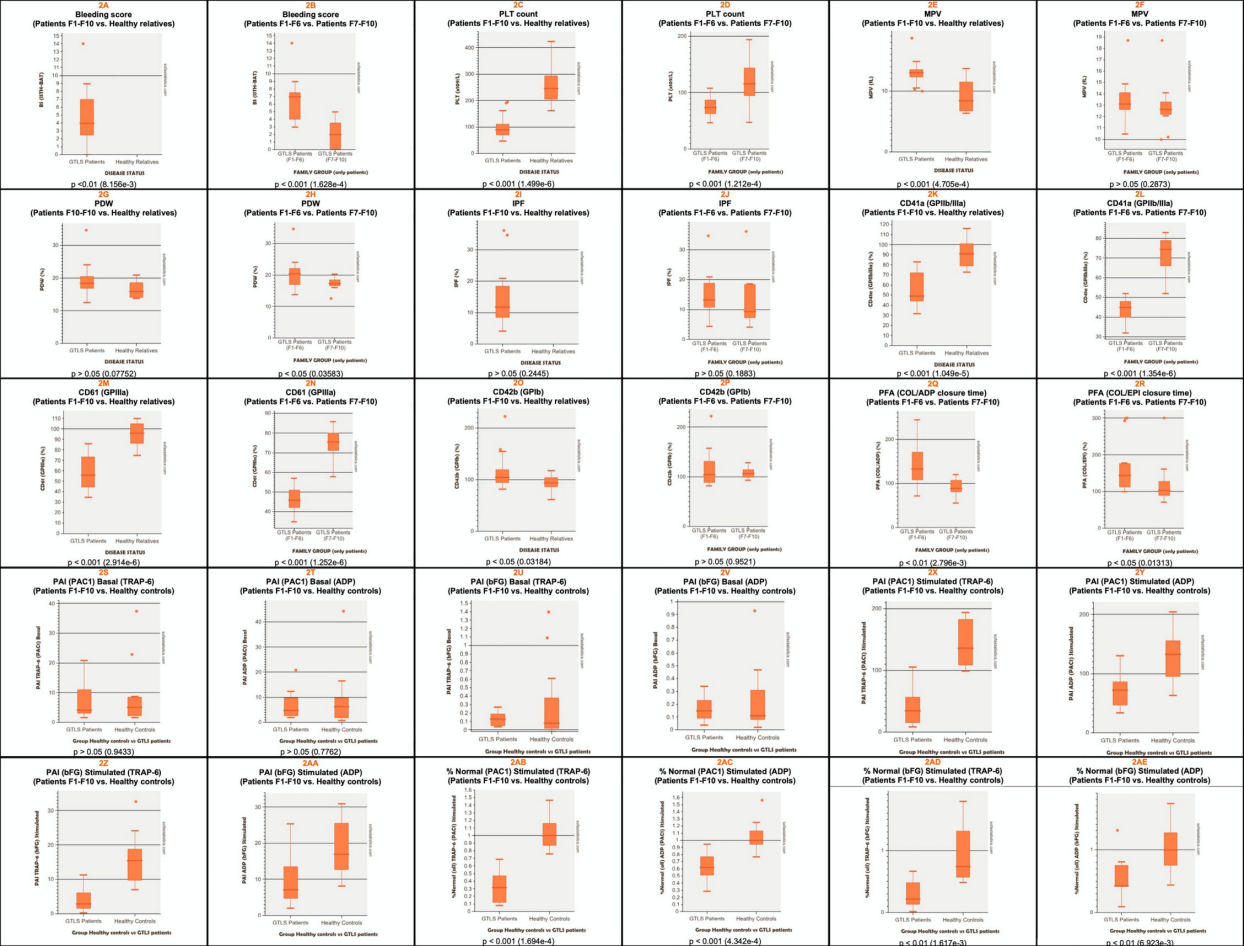
Comparison between GTLS patients, GTLS families and healthy relatives for the parameters analyzed in this study. Abbreviations: ADP, Adenosine diphosphate; bFG, bound fibrinogen; BS, Bleeding score (ISTH/BAT); COL/ADP, collagen / ADP; COL/EPI, collagen / epinephrine; IPF, immature platelet fraction (%); GTLS, Glanzmann Thrombasthenia like syndrome; MPV, mean platelet volume (fL); PDW, Platelet volume distribution width (%); PLT, platelets (x10^9^/L); PAI, platelet activation index; PFA, Platelet Function Assay; TRAP-6, Thrombin Receptor Agonist Peptide-6. Data information: Outliers displayed. Lower whiskers are 1.5 times the Inter-Quartile Range below the lower quartile, or the minimum value, whichever is closest to the middle. Upper whiskers are calculated using the same approach. P values were obtained using the Mann-Whitney test.

**Fig 3:**
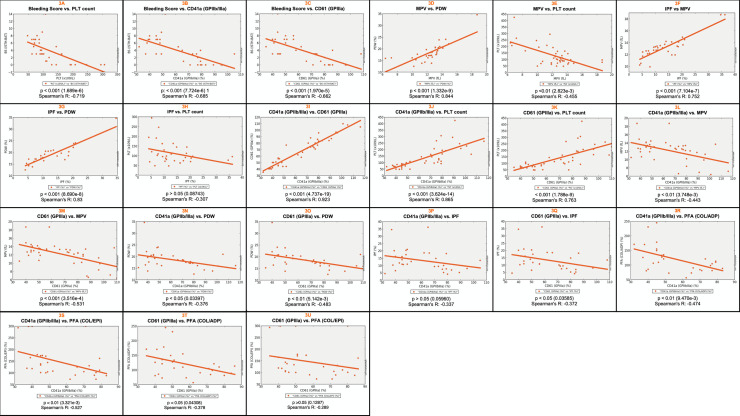
Correlations between the parameters evaluated in GTLS patients, including bleeding scores, platelet counts, platelet indexes, platelet function assays and platelet glycoprotein expression levels. Abbreviations: COL/ADP, collagen / ADP; COL/EPI, collagen / epinephrine; IPF, immature platelet fraction (%); GTLS, Glanzmann Thrombasthenia like syndrome; MPV, mean platelet volume (fL); PDW, Platelet volume distribution (%) width; PLT, platelets (x10^9^/L); PFA, Platelet Function Assay. P values were obtained using the Spearman´s R correlation test.

**Fig 4:**
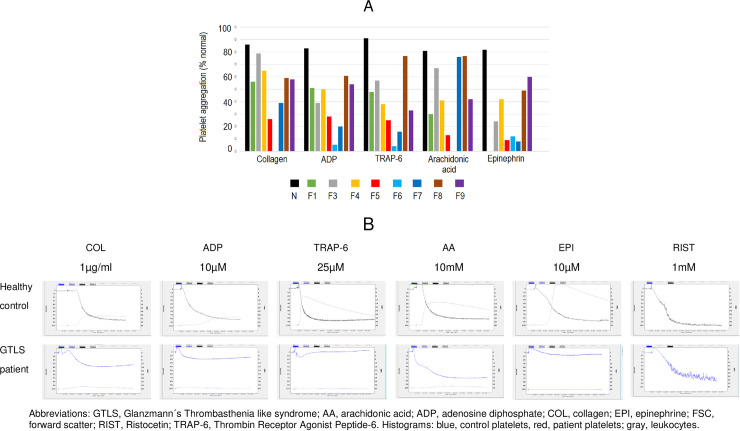
Platelet aggregation/agglutination in response to different agonists from a representative healthy control (N) and representative patients from GTLS families (panel A) obtained by lumiaggregometry, and platelet aggregation/agglutination tracings, from a representative healthy control and a representative GTLS patient (panel B). Abbreviations: GTLS, Glanzmann´s Thrombasthenia like syndrome; AA, arachidonic acid; ADP, adenosine diphosphate; COL, collagen; EPI, epinephrine; FSC, forward scatter; RIST, Ristocetin; TRAP-6, Thrombin Receptor Agonist Peptide-6. Histograms: blue, control platelets, red, patient platelets; gray, leukocytes.

The main purpose of this work is to present 10 new families with GTLS (ITGA2B/ITGB3-RT) with an identified genetic defect, whose pedigrees are depicted in **Fig 1**. They include 33 patients (25 females, 75.8%), median age at the diagnosis 30 years, ranging from 1 to 84 years) and 11 unaffected relatives (8 females, 72.7%), median age at the time of the study 14 years, ranging from 1 to 42 years. Their BS, PLT counts and indexes, PFA closure times, and PLT glycoprotein levels are summarized in **Table 1** and described individually in **S3 File.** The results from PLT lumiaggregometry assays are illustrated in **Fig 2**, and described in detail in **S4 File,** and the results from FCM based PLT activation studies are summarized in **Table 2** and detailed in **S5 File.** Comparisons between groups for the quantitative parameters analyzed in this study are depicted in **Fig 3**, and their correlations are shown in **Fig 4**. Finally, the genetic screening studies are summarized in **Table 3** and the variants detected in the *ITGA2B* and *ITGA3* genes are presented in **Table 4**.

**Table 1:** Summary of the bleeding scores and the PLT counts and indexes, function assays and GP levels in GTLS patients and healthy relatives.

	GTLS patients(F1-F10) (n = 33)	GTLS patients (F1-F6) (n = 19)	GTLS patients (F7-F10) (n = 14)	Healthy relatives(n = 11)
**Bleeding Score (ISTH-BAT)**				
BS	**4** (0–14)	**7** (3–14)	**2** (0–5)	**0** (0–3)
	4±3	6±3	2±2	1±1
Abnormal BS	10/29 (34%)	10/16 (63%)	0/13 (0%)	0/5 (0%)
**Platelet count, x10**^**9**^**/L**				
150–450	**90** (47–194)	**74** (47–108)	**116** (48–194)	**246** (163–425)
	96±38	76±17	124±40	252±74
<150	30/33 (91%)	19/19 (100%)	11/14 (79%)	0/11 (0%)
<100	22/30 (67%)	18/19 (95%)	4/14 (29%)	0/11 (0%)
<50	3/33 (9%)	2/19 (11%)	1/14 (7%)	0/11 (0%)
**Platelet indexes**				
** Mean Platelet Volume (MPV), fL**				
[7–11]	**13** (10–19)	**13** (11–19)	**13** (10–19)	**8** (6–14)
	13±2	13±2	13±2	9±3
>11	28/32 (88%)	16/18 (89%)	12/14 (86%)	3/9 (33%)
** Platelet Distribution Width (PDW), %**				
[9–14]	**18** (13–35)	**21** (14–35)	**17** (13–20)	**16** (14–21)
	19±4	20±5	17±2	16±3
> 14	25/27 (93%)	14/15 (93%)	11/12 (92%)	4/5 (80%)
** Immature Platelet Fraction (IPF), %**				
[1–7]	**12** (4–36)	**13** (5–35)	**9** (4–36)	**8** (5–14)
	14±8	15±7	13±9	9±4
> 7	24/29 (83%)	14/16 (88%)	10/13 (77%)	2/3 (67%)
**Platelet Function Assays (PFA)**				
**COL/ADP Closure Time, seconds**				
[56–120]	**110** (56–245)	**133** (72–245)	**89** (56–121)	NA
	120±45	141±48	92±19	NA
> 120	**12/29** (41%)	**11/17** (65%)	**1/12** (8%)	NA
** COL/EPI Closure Time, seconds**				
[88–146]	**125** (72–300)	**144** (100–300)	**103** (72–300)	NA
	147±69	166±68	120±62	NA
> 150	10/29 (34%)	8/17 (47%)	2/12 (17%)	NA
**Platelet glycoproteins (GP)**				
** CD41a (GPIIb/IIIa), % normal**				
[85–115]	**49** (32–83)	**45** (32–52)	**75** (52–83)	**91** (73–116)
	56±16	44±5	72±9	91±13
<85	33/33 (100%)	19/19 (100%)	14/14 (100%)	4/11 (36%)
** CD61 (GPIIIa), % normal**				
[80–120]	**56** (35–86)	**46** (35–57)	**76** (58–86)	**96** (75–110)
	59±16	47±6	75±8	94±11
<80	29/33 (88%)	21/21 (100%)	10/14 (71%)	1/11 (9%)
** CD42b (GPIb), % normal**				
[70–130]	**105** (82–222)	**105** (82–222)	**107** (93–129)	**94** (62–118)
	112±28	116±36	108±10	94±14
>130	5/32 (16%)	5/18 (28%)	0/14 (0%)	0/11 (0%)

Abbreviations: BS, Bleeding score; COL/ADP, collagen / ADP; COL/EPI, collagen / epinephrine; GP, glycoprotein; GTLS, Glanzmann Thrombasthenia like syndrome; ISTH-BAT, International Society on Thrombosis and Hemostasis–Bleeding Assessment Tool; PLT, platelets; Results are presented as median (minimum–maximum) values, and mean ± one standard deviation, as well as absolute and relative frequencies (percentage of cases) fulfilling the mentioned criteria. Normal reference values are indicated between square brackets.

BS (ISTH-BAT): the cut-off used for an abnormal BS was ≥4 in adult males, ≥6 in adult females and ≥3 in children [29].

**Table 2:** Summary of the basal and agonist induced PLT activation status as determined by FCM, in representative patients from the GTLS families and healthy controls, expressed as platelet activation indexes and as percentages of the normal values.

	Healthy controls (Blood donors)(n = 12)	GTLS patients(F1-F10)(n = 9)	GTLS patients (F1-F6)(n = 6)	GTLS patients (F7-F10)(n = 3)
**Platelet activation indexes (PAI)**				
**TRAP-6 experiments**				
**AIBS (PAC-1)**				
Basal	**5** (2–37)	**4** (2–21)	**4** (2–9)	**13** (7–21)
	9±11	7±7	4±3	14±7
*P value*	*>0*.*05*		***<0*.*05***	
Stimulated	**136** (100–194)	**35** (8–106)	**24** (8–68)	**37** (35–107)
	144±39	40±33	30±23	59±40
*P value*	***< 0*.*001 (3*.*077e-4)***		*>0*.*05*	
**RIBS (bFG)**				
Basal	**0** (0–1)	**0** (0–0)	**0** (0–0)	**0** (0–0)
	0±1	0±0	0±0	0±0
*P value*	*>0*.*05*		*>0*.*05*	
Stimulated	**13** (7–33)	**3** (0–11)	**2** (0–7)	**3** (3–11)
	15±8	4±4	3±3	6±5
*P value*	***< 0*.*001 (4*.*112e-4)***		*>0*.*05*	
**ADP experiments**				
**AIBS (PAC-1)**				
Basal	**6** (1–44)	**5** (2–21)	**3** (2–7)	**12** (7–21)
	9±12	7±7	4±2	13±7
*P value*	*>0*.*05*		***<0*.*05***	
Stimulated	**133** (63–205)	**73** (34–130)	**65** (34–84)	**88** (53–130)
	130±40	71±36	61±21	90±39
P value	**<0.01 (2.521e-3)**		>0.05	
**RIBS (bFG)**				
Basal	**0** (0–1)	**0** (0–0)	**0** (0–0)	**0** (0–0)
	0±0	0±0	0±0	0±0
P value	>0.05		>0.05	
Stimulated	**17** (8–31)	**7** (2–25)	**6** (2–13)	**14** (6–25)
	19±9	10±8	7±4	15±10
*P value*	***<0*.*01 (6*.*923e-3)***		*>0*.*05*	
**% of the normal values**				
**TRAP-6 experiments**				
AIBS (PAC-1), stimulated	**100** (76–147)	**32** (8–69)	**23** (8–51)	**36** (25–69)
	102±29	32±22	26±18	43±23
*P value*	***< 0*.*001 (1*.*694e-4)***		*>0*.*05*	
RIBS (bFG), stimulated	**100** (40–180)	**22** (2–67)	**20** (2–61)	**22** (19–67)
	101±49	29±23	25±21	36±27
*P value*	***< 0*.*01 (1*.*617e-3)***		*>0*.*05*	
**ADP experiments**				
AIBS (PAC-1), stimulated	**100** (77–156)	**62** (29–95)	**60** (29–84)	**64** (56–95)
	106±20	63±26	58±19	71±21
*P value*	***< 0*.*001 (4*.*342e-4)***		*>0*.*05*	
RIBS (bFG), stimulated	**100** (44–174)	**43** (10–131)	**43** (10–69)	**81** (41–131)
	100±38	57±40	43±20	84±46
*P value*	***< 0*.*01 (6*.*923e-3)***		*>0*.*05*	

Abbreviations: ADP, Adenosine diphosphate; AIBS, activation-induced binding sites on the GPIIb/IIIa receptor; BAS, basal conditions (PBS, no agonist); bFG, bound fibrinogen; FCM, flow cytometry; FI, fold increase; GT, Glanzmann Thrombasthenia; GTLS, Glanzmann Thrombasthenia like syndrome; NA, not available; PAI, platelet activation index; PBS, phosphate buffered saline; PLT, platelet; RIBS, receptor-induced binding sites on the fibrinogen ligand; TRAP-6, Thrombin Receptor Agonist Peptide-6.

Test samples were either unstimulated samples (PBS), or samples stimulated with TRAP-6 (20 μM) or with ADP (10 μM).

Platelet activation was studied using either FITC conjugated anti-PAC-1 or anti-bFG mAbs.

P values were calculated using the Mann-Whitney test.

**Table 3:** Summary of the genetic studies performed in the study GTLS families.

GTLS Families	Individuals	GTLS Patients	Healthy relatives	Affected gene		Genetic studies	Heterozygous mutation(+/-)	Non-mutated(-/-)
**F1**	12	8	4	*ITGA2B*		4	4	0
**F2**	2	2	0	*ITGA2B*		2	2	0
**F3**	3	2	1	*ITGA2B*		3	2	1
**F4**	1	1	0	*ITGA2B*		1	1	0
**F5**	2	2	0	*ITGA2B*		2	2	0
**F6**	6	4	2	*ITGB3*		5	4	1
**F7**	3	2	1	*ITGB3*		3	2	1
**F8**	5	4	1	*ITGB3*		4	3	1
**F9**	8	6	2	*ITGB3*		8	6	2
**F10**	2	2	0	*ITGB3*		2	2	0
**All families**	44(100%)	33(75%)	11(25%)	*ITGA2B*(5)	*ITGB3* (5)	34(100%)	28(82%)	6 (18%)

Abbreviations: GTLS, Glanzmann Thrombasthenia like syndrome.

**Table 4:** Genetic defects identified in the different families with thrombocytopenia, affecting the *ITGA2B* or the *ITGB3* genes.

Families	Gene (region)	Variants		Protein region	Frequency in gnomAD database	Classification (according to the ACMG guidelines) and applicable criteria	Reference
		DNA	Protein				
1, 2, 3	*ITGA2B*(exon 30)	NM_000419.4:c.3076C>T	p.(Arg1026Trp) p.(Arg995Trp)#	C-terminal cytoplasmic region, conserved site	Not listed	Pathogenic (by legacy)	[19]
4	*ITGA2B*(exon 30)	NM_000419.4:c.3077G>A	p.(Arg1026Gln)p.(Arg995Gln)#	C-terminal cytoplasmic region, conserved site	ALL: 0.00040%	Pathogenic (by legacy)	[15]
5	*ITGA2B*(exon 29)	NM_000419.4:c.3020G>T	p.(Gly1007Val)p.(Gly976Val)#	Integrin alpha chain, transmembrane	Not listed	VUS(PM2, PP1, PP2, PP3)	New
6	*ITGB3* (exon 14)	NM_000212.2:c.2236A>C	p.(Thr746Pro)p.(Thr720Pro)#	Integrin beta subunit, cytoplasmic	Not listed	VUS(PM2, PP1, PP2, PP3)	New
7	*ITGB3*(exon 14)	NM_000212.2:c.2243A>C	p.(His748Pro)p.(His722Pro)#	Integrin beta subunit, cytoplasmic	Not listed	VUS(PM2, PP1, PP2, PP3)	New
8, 9	*ITGB3*(exon 14)	NM_000212.2:c.2278C>T	p.(Arg760Cys)p.(Arg734Cys)#	Integrin beta subunit, cytoplasmic	Not listed	Likely-pathogenic(PS4, PM2, PP1, PP2, PP3)	New
10	*ITGB3*(exon 14)	NM_000212.2:c.2245G>C	p.(Asp749His)p.(Asp723His)#	Integrin beta subunit, cytoplasmic	Not listed	Pathogenic (by legacy)	[31]

Abbreviations: ACMG, American College of Medical Genetics and Genomics; PM2, Pathogenic Moderate criteria 2; PP1, Pathogenic Supporting criteria 1; PP2, Pathogenic Supporting criteria 2; PP3, Pathogenic Supporting criteria 3; PS4, Pathogenic Strong criteria 4; VUS, variant of unknown significance.

# In terms of legacy, we included the description of variants used for previous publications, where the signal peptide sequences (31 residues for αIIb and 26 residues for β3) was included.

### Clinical findings and family history

#### Family 1

The first patient with ITGA2B/ITGB3-*RT* identified by our group (F1.III.4, index, BS = 8) was a 26 years old female studied in 1995 consequently to having been submitted to tonsillectomy complicated by hemorrhage and needing to be transfused with red blood cells (RBC) (Fig 1) (S3 File). The patient subsequently underwent two caesarean sections and spinal surgery with desmopressin without bleeding, and she had no significant hemorrhagic symptoms in addition to easy bruising. Blood counts revealed moderate thrombocytopenia (80x10^9^/L) with PLT macrocytosis and anisocytosis. When searching for antiplatelet antibodies by FCM (negative results), we occasionally detected a decreased expression of CD41 (GPIIb/IIIa) on the PLT membrane, which was confirmed in subsequent studies. The patient had decreased PLT aggregation and diminished ATP release with the agonists ADP, collagen, and AA, with normal ristocetin agglutination. She had three sisters aged between 21 to 30 years, all with thrombocytopenia (47-80x10^9^/L) and a common hemorrhagic pattern: easy bruising and nose bleeding during infancy. Subsequent family studies revealed that her mother and grandmother had an identical phenotype, suggesting an AD inheritance, and co-segregation of thrombocytopenia and decreased GPIIb/IIIa in the affected patients was confirmed (Fig 1) (S3 File). By reviewing the literature, at that time we found only one case with possibly identical characteristics [14], and we assumed that these patients had a “GT variant”.

#### Family 2

Patient F2.II.1 (index, BS = 3), 17 years old male, was referred to our center in 2014 (Fig 1) (S3 File). He reported only mild bruises after minor trauma and had PLT counts between 90 and 100x10^9^/L, with PLT anisocytosis. His mother (F2.I.2; BS = 14) had a history of pregnancy with thrombocytopenia, requiring labor induction at 35 weeks of gestation; cesarean delivery had been complicated by postpartum hemorrhage, demanding hospitalization in Intensive Care, and RBC and PLT transfusions; she also reported bleeding after tooth brushing and easy bruising without trauma. His brother, who was 13 years old, had frequent epistaxis but the PLT counts and GP levels were normal.

#### Family 3

Patient F3.II.1 (index, BS = 3), male, was referred to our consultation in 2018, when he was 64 years old, for investigation of moderate thrombocytopenia, that had been detected in routine tests (Fig 1) (S3 File). He reported severe epistaxis as a child, but he had had multiple dental extractions without hemorrhage; there was no history of surgeries. His son (F3.III.2) had thrombocytopenia detected during blood donation. His mother also had thrombocytopenia and his daughter had normal PLT counts.

#### Family 4

Patient F4.II.1 (index, BS = 9), male, was sent to our consultation in 2014, when he was 48 years old, to clarify thrombocytopenia (Fig 1) (S3 File). He reported bleeding after tonsillectomy at 7 years of age and epistaxis in childhood, as well as easy ecchymosis and gingival bleeding. He had a family history of thrombocytopenia (son, sister and two nephews), but these relatives have not yet been studied.

#### Family 5

Patient F5.III.1 (index, BS = 6), female, was diagnosed in our hospital in 1997, when she was 16 years old (Fig 1) (S3 File). She had a family history of thrombocytopenia (father and paternal grandmother). The hemorrhagic symptoms consisted of easy bruising and menometrorrhagia, and she had no history of surgeries. Her father (F5.II.1), who was diagnosed in the same year, had prolonged hemorrhages after cuts and had been submitted to hemorrhoid surgery without hemorrhagic complications.

#### Family 6

Patient F6.II.4 (index, BS = 6), female, was diagnosed in 1996, when she was 30 years old (Fig 1) (S3 File). She mentioned easy bruising and gingival bleeding; she also referred two dental extractions complicated with hemorrhage and two deliveries without bleeding. Her father (F6.I.1), also diagnosed in 1996, had easy bruising without other relevant hemorrhagic manifestations, and suffered from multiple comorbidities, being medicated with anti-aggregation therapy and with direct oral anticoagulants. In 2017, at the age of 86, he had gastrointestinal bleeding due to duodenal angiodysplasia requiring RBC transfusions and intravenous iron. His condition stabilized after stopping oral anticoagulants. Her two daughters were also affected by the disease.

#### Family 7

Patients F7.II.2 and F7.II.4, females, are sisters who were studied in 2019, when they were 45 and 44 years old, respectively, in the context of an investigation of familial thrombocytopenia detected in their mother (Fig 1) (S3 File). Patient F7.II.2 (index, BS = 4), had mild thrombocytopenia detected nine years earlier, and previously interpreted as autoimmune, for having autoimmune thyroiditis. Patient F7.II.4 (BS = 5), also with mild thrombocytopenia, had a history of postpartum hemorrhage needing RBC transfusions, easy bruising and abundant menstruations.

#### Family 8

Patient F8.I.2 (index), female, was studied in 1978, at the age of 75, after being referred to our hospital for cholecystectomy, performed after prophylactic PLT transfusion without bleeding complications (Fig 1) (S3 File). Her thrombocytopenia had been detected 15 years earlier, at which time the diagnosis of chronic idiopathic thrombocytopenia had been assumed. She mentioned haemorrhage after tooth extraction and a family history of thrombocytopenia (two nephews). Subsequently, several family members were diagnosed with thrombocytopenia. Some of them have been studied, including her daughter (F8.II.2), two granddaughters (F8.III.2, with thrombocytopenia detected during gestation and F8.III.3, unaffected) and one great-granddaughter (F8.IV.1), who had no relevant bleeding.

#### Family 9

Patient F9.II.2 (index; BS = 3), female, was first studied in 2017, when she was 37 years old, because of thrombocytopenia, first detected in 1998, by the time of her first pregnancy (Fig 1) (S3 File). She referred easy bruising with minor trauma and occasional gingival bleeding. She had had three deliveries without hemorrhagic complications, having had pre-partum prophylactic PLT transfusions. She reported a family history of thrombocytopenia (mother, aunt and one sister). Her PLT count varied between 95 and 158x10^9^/L. One of her sisters (F9.II.4; BS = 3) also had thrombocytopenia detected in two pregnancies, which had been interpreted as gestational; she underwent PLT transfusions on her first cesarean section and corticoid therapy during the second pregnancy. However, she maintained moderate thrombocytopenia after delivery. She had had three previous surgeries (two caesarean sections and ear surgery) and dental extractions without bleeding.

#### Family 10

Patient F10.II.3 (index; BS = 1), a 74 years old male, was sent to our consultation in 2019 to clarify moderate thrombocytopenia known since 2004 (Fig 1) (S3 File). At that time, he was anticoagulated with rivaroxaban due to atrial fibrillation and he was doubly anti-aggregated for ischemic heart disease. He had no previous hemorrhagic symptoms, although he had never undergone surgery. Since he started anticoagulation and anti-aggregation, he suffered from gingival bleeding and easy bruising. Several family members (daughter, brother, sister and nephew) with thrombocytopenia have been identified, but only one daughter has been studied so far.

### Bleeding tendency

The patients had absent to moderate bleeding (median BS = 4), with F1-F6 having higher BS (median = 7) than F7-F10 (median = 2) (p<0.001) (**Table 1**) (**Fig 2A and 2B**). Patients from F1-F6 always had thrombocytopenia and they experienced hemorrhage episodes whenever they underwent hemostatic challenges such as surgeries. In contrast, bleeding was absent in F7-F10, where some patients alternate between normal or slightly decreased PLT counts and others even had normal PLT values. The BS showed a strong negative correlation with the PLT counts (p<0.001) (**Fig 3A**), as well as with the levels of CD41 (GPIIb/IIIa) and CD61 (GPIIIa) expression (p<0.001) (**Fig 3B and 3C**).

### Platelet counts, indexes and morphology

Mild to moderate thrombocytopenia (<150x10^9^/L) was a recurrent finding, being present in 30/33 patients (91%), with a median PLT count of 90x10^9^/L, and in none of the healthy relatives, who had a median PLT count of 246x10^9^/L (**Table 1**) (**Fig 2C**) (p<0.001). Families 1 to 6 had lower PLT counts than F7-10 (median PLT count of 74 vs. 116x10^9^/L) (p<0.001) (**Table 1**) (**Fig 2D**) (p<0.001). All patients from F1-F6 have thrombocytopenia, whereas F7-F10 include patients with normal PLT counts (e.g., P8.III.2 with thrombocytopenia during pregnancy, F8.IV.1, a 1-year-old child, F8.III.2’s daughter and F9.III.1) or with episodic thrombocytopenia (e.g., F9.II.2). Platelet counts <100x10^9^/L were observed in 18/19 patients from F1-F6 (95%), but only in 4/14 patients from F7-F10 (29%); and PLT counts <50 x10^9^/L were found in only 3 cases (9%) (**Table 1**).

The MPV was increased (>11fL) in 28/32 patients tested (88%), with a median value of 13fL, which was significantly higher compared to the healthy relatives, whose median MPV was of 8 (p<0.001) (**Table 1**) (**Fig 2E**); the median MPV observed in patients from F1-F6 and from F7-F10 were identical (13fL) (p>0.05) (**Table 1**) (**Fig 2F**). Also, the PDW was increased (>14%) in 25/27 patients tested (93%), with a median value of 18%, which did not differ significantly from the values observed in healthy relatives (16%, only 5 cases tested) (p = 0.077) (**Table 1**) (**Fig 2G**). The median PDW values observed in patients from F1-F6 was somewhat higher than in F7-F10 (21% and 17%, respectively) (p<0.005) (**Table 1**) (**Fig 2H**). There was a strong positive correlation between the MPV and the PDW (p<0.001) (**Fig 3D**), and a moderate negative correlation between the MPV and the PLT counts (p<0.01) (**Fig 3E**).

Curiously, the MPV values were also increased in some of the healthy relatives tested (3/9, 33%) (median value: 8fL), most of the cases evaluated having PLT anisocytosis (increased PDW in 4/5 cases, 80%), with a median PDW of 16% (**Table 1**). The PB smears revealed PLT macrocytosis and anisocytosis in most of the patients analyzed, but also in some unaffected relatives, sometimes with a variable fraction of giant PLT; small PLT aggregates were observed in some cases (**S3 File**).

The IPF, which estimates the fraction of young PLTs that have recently been released into the circulation by measuring reticulated, i.e., ribonucleic acid (RNA)-containing PLTs in the PB and is considered an index of thrombopoiesis [30], was increased (>7%) in 24/29 patients tested (83%), with a median value of 12%, comparatively to 8%, observed in healthy relatives (only 3 cases analyzed) (**Table 1**) (**Fig 2I**) (p>0.05). Also, patients from F1-F6 had higher IPF values as compared to patients from F7-F9 (median values: 13% and 9%, respectively), although differences did not reach statistical significance (p>0.05) (**Table 1**) (**Fig 2J**), maybe because of variability and the low number of cases studied. The IPF correlated positively with the MPV and the PDW values (p<0.001) (**Fig 3F and 3G**), but the negative correlation with the PLT counts did not reach statistical significance (p >0.05) (**Fig 3H**). Increased IPF values were previously described in GTLS [31].

Previous studies have indicated that at least some of the genetic variants found in GTLS patients, such as αIIb: p.Arg1026Trp [19] and β3: p.Asp749His [31], do interfere with PLT production, probably explaining the thrombocytopenia, but also the abnormal PLT indexes (MCV, PDW) and morphology, as well as the increased IPF observed in these patients. These studies have shown that αIIbβ3: p.Asp749His-transfected CHO cells, an epithelial cell line derived from the ovary of the Chinese hamster, had increased anti-PAC-1 binding [31], and, as αIIb: p.Arg1026Trp/β3-transfected CHO cells [19], exhibited membrane ruffling and abnormal cytoplasmic protrusions [31]; in addition, megakaryocytes derived from CD34+ stem cells from patients harboring the β3: p.Asp749His mutation [31] and αIIb: p.Arg1026Trp/β3-transfected megakaryocytes derived from mouse fetal liver cells [19] were found to have abnormal proplatelet formation.

### Platelet glycoproteins

The levels of CD41a (GPIIb/IIIa) expression on the PLT surface, as determined by FCM, were consistently low in patients, with a median value of 49%, comparatively to healthy relatives (median value: 91%) (normal reference interval: 85–115%) (p<0.001) (**Table 1**) (**Fig 2K**). It should however be noted that 4/11 healthy relatives studied (36% of cases) had slightly decreased levels of GPIIb/IIIa expression with a median value of 77% (**Table 1**). Patients from F1-F6 had median GPIIb/IIIa levels lower than those observed in patients from F7-F10, who had only marginally decreased levels of αIIbβ3 integrin (median values: 45% and 75%, respectively) (p<0.001) (**Table 1**) (**Fig 2L**). The same was observed for CD61 (GPIIIa) which was also lower in patients than in healthy relatives (median values: 46% and 96%) (normal reference interval: 80–120%) (**Table 1**) (**Fig 2M**), and lower in patients from F1-F6 (median value: 46%) comparatively to patients from F7-F10 (median value: 76%) (p<0.001) (**Table 1**) (**Fig 2N**).

Normal or increased levels of CD42b (GPIb) were observed in all GTLS patients (median value: 105%) (normal reference interval: 70–130%) (p<0.05) (**Table 1**) (**Fig 2O**). We have no explanation for the increased levels of GPIb observed in some patients. It cannot be explained by the increased PLT volume because platelet GP levels were corrected for PLT size, as evaluated by the FSC. This phenomenon affects particularly some of the families, i.e. F1, harboring the αIIbR995W variant, with five of seven patients tested (71%) having increased GPIb levels (**S3 File)**. Similar results were obtained in other cases described in the literature [19,32]. Differences for CD42b between patients from F1-F6 and F7-F10 did not reach statistical significance (**Table 1**) (**Fig 2P**).

As expected, and considering all cases studied, platelet GPIIb/IIIa and GPIIIa levels strongly correlated to each other (p<0.001) (**Fig 3I**). In addition, there was strong positive correlation with the PLT counts (p<0.001) (**Fig 3J and 3K**). Furthermore, GPIIb/IIIa and GPIIIa expression levels correlated inversely and moderately with the MPV values (p<0.01 and <0.001, respectively) (**Fig 3L and 3M**), as well as with the PDW values (p<0.05 and <0.01, respectively) (**Fig 3N and 3O**). Also, the levels of GPIIb/IIIa and GPIIIa expression showed a tendency for a negative correlation with the IPF values (p = 0.059 and p = 0.035, respectively) (**Fig 3P and 3Q**). In considering the PFA closure times, we found a moderate negative correlation between the levels of CD41 (GPIIb/IIIa) expression and the COL/ADP and COL/EPI closure times (p<0.01) (**Fig 3R and 3S**). Less strong correlations were observed with CD61 (GPIIIa) (p<0.05 and p>0.05, respectively) (**Fig 3T and 3U**). No significant correlations were found for CD42b (GPIb), except for a weak negative correlation with the PLT counts (p = <0.05).

### Platelet functional defects

Conventional laboratory tests such as PFA closure times, PLT aggregation and ATP release assays by lumiaggregometry, provide important and complementary information useful for diagnosing and classifying PLT disorders. They are shown in **Table 1** and **Fig 4,** where the results of a representative patient per family are presented (except for F2 and F10, who have not lumiaggregometry studies).

Patients from F1-F6 had higher COL/ADP closure times, as determined by PFA, as compared to patients from F7-F9 (median values: 133 and 89 seconds, respectively) (p<0.01) (**Fig 2Q**). The same was observed for COL/EPI, although differences were not so evident (median values: 144 and 73 seconds, respectively) (p<0.05) (**Fig 2R**). It should however be noted that in patients having several PFA determinations, there was some fluctuation of the COL/EPI and COL/ADP closure times overtime (**S4 File**). Markedly increased closure times, such as those typically found in GT, have never been observed, and the highest closure times were observed in F1 and F2; patients from F7-F10 always had normal closure time values.

Platelet lumiaggregometry studies revealed decreased aggregation and diminished or absent ATP-release in response to the agonists ADP, TRAP-6, collagen, arachidonic acid and epinephrine, and all but one patient studied (F5) had normal ristocetin-induced PLT agglutination (**Fig 4) (S4 File)**. However, there was great variability among families. For example, F1, F3 and F4 showed reduced response with the agonists studied, with variable decrease according to the agonist (the response was most reduced with epinephrine and better with collagen), and impaired ATP release, even absent in F3. Families 7 to 9 shared the same pattern of aggregation and ATP release of the families mentioned above. On the other hand, F5 and specially F6 had much more reduced or even absent aggregation and ATP release.

### Flow cytometry-based platelet activation studies

Flow cytometric analysis of PLT function has added value in diagnostics of PLT disorders [33–36]. In particular, FCM-based PLT activation studies allow measuring different types of activation-related epitopes on the GPIIb/IIIa receptor and its major ligand, FG, either at basal conditions or upon stimulation with physiological agonists [37]. These include: i) activation-induced binding sites (AIBS) on αIIbβ3 resulting from its conformational change, with exposure of FG binding sites (prototypic antibody: clone PAC1) [38]; ii) ligand-induced binding sites (LIBS) on activated αIIbβ3, as a consequence of FG binding (prototypic antibodies: clones PM 1.1, LIBS1 and LIBSG, not used in this study) [39–41]; and iii) receptor-induced binding sites (RIBS) on the FG ligand (prototypic antibodies: clones 2G5, 9F9 and F26) [42–45]. In this work, we focused on two consecutive steps: the conformational change of αIIbβ3 after activation, with exposure of FG binding sites (PAC-1); and binding of FG to these sites, with subsequent expression of new epitopes on the bound fibrinogen (bFG) molecule (9F9).

The results from FCM based PLT activation studies, performed in 9 patients from 8 out of 10 GTLS families reported herein are summarized in **Table 2**. The original data obtained for GTLS patients and healthy controls studied in parallel are provided in **S5 File**. Unfortunately, these studies were not available in F3 (none of the patients were available) and F10 (the index case was receiving anticoagulation and double anti-aggregation and his daughter was unavailable).

### Basal platelet activation

Under basal conditions (no agonist), PLTs from most healthy controls studied showed only residual constitutive activation as evaluated by the expression of AIBS, having a median basal PAI of 5 and 6 when evaluated with anti-PAC-1 in TRAP-6 and ADP experiments (i.e., without stimulation, the median MFI of anti-PAC-1 staining was about 5–6 times higher than that observed in inhibitory conditions, that is, in the presence of EDTA); curiously, the median basal PAI was of “0”, when evaluated by the expression of RIBS (bFG) both for TRAP-6 and ADP experiments (**Table 2**). Of note, two out of twelve controls (C9 and C10) had basal PAI/PAC-1 values clearly higher than the others, both in TRAP-6 experiments (23 and 37 for C9 and C10, respectively, vs. 2 to 9 for the other controls) and in ADP experiments (basal PAI of 17 and 44 for C9 and C10, respectively, vs. 1 to 11 for the other controls), suggesting increased levels of constitutive PLT activation (**S5 File**). However, there was no evidence of basal PLT activation when measuring RIBS with anti-bFG mAbs, neither in TRAP-6 (basal PAI of 1 for C9 and C10, respectively, vs. 0 to 1 for the other controls), nor in ADP experiments (basal PAI of “0” and “1” for C9 and C10, respectively, vs. 0 to 1 for the other controls) (**S5 File**).

Platelets from GTLS patients had median basal PAI values of 4 and 5 when evaluated with anti-PAC-1 in TRAP-6 and ADP experiments, respectively (**Table 2**), which were not significantly different from the values obtained in healthy controls (p>0.05) (**Fig 2S and 2T**). Two out of nine patients (F8.II.2 and F9.III.2) however, had basal PAI values clearly higher compared to other patients, when evaluated by anti-PAC-1, both in TRAP-6 (basal PAI of 21 and 13 for patients F8.II.2 and F9.III.2, respectively vs. 2 to 9 observed in the remaining patients) and in ADP experiments (basal PAI values of 21 and 12 for patients F8.II.2 and F9.III.2, respectively vs. 2 to 7 observed in the remaining patients) (**S5 File**). As in controls, we found no evidence of basal PLT activation when measuring RIBS (bFG) (**Fig 2U and 2V**) (p>0.05), including for patients F8.II.2 and F9.III.2, who had basal PLT activation evaluated by the expression of AIBS (PAC-1) (**S5 File**).

Altogether, the results obtained would suggest that the residual PLT activation observed in some individuals (either healthy controls or GTLS patients) with anti-PAC-1 is abortive, in the sense that it did not translate into efficient FG binding to the αIIbβ3 receptor. The possibility of an artefactual GPIIb/IIIa activation during the processing of these samples (C9, C10 and F8.II.2 and F9.III.2) cannot be completely excluded. However, it seems unlikely because the study of one of the patients (F8.II.2) was repeated in a different day, giving the same results.

It has been assumed that at least some of the pathogenic variants associated with GTLS (ITGA2B/ITGB3-RT) cause constitutive activation of the integrin receptor [19,31,32]. In our study, most of the GTLS patients studied, similarly to that observed in healthy controls, showed only residual levels of constitutive αIIbβ3 activation as evaluated by the expression of AIBS under basal conditions (no agonist). The only exceptions were patients with the β3: p.Arg760Cys variant who had basal PAIs clearly higher when evaluating AIBS on the αIIbβ3 using anti-PAC-1, but no evidence of basal PLT activation when measuring RIBS on its ligand, FG, using anti-bFG mAbs. Curiously, these patients had less pronounced PLT defects. Thus, our results are not in accordance with previous observations in the literature.

Ghevaert *et al*., reported, in 2008, that PLTs from GTLS patients having the β3: p.Asp749His had constitutive αIIbβ3 activation, as documented by spontaneous anti-PAC1 binding, but not FG binding [31]. Kunishima *et al*., 2011, also observed that PLTs from patients with αIIb: p.Arg1026Trp had spontaneous anti-PAC-1 binding [19]. However, Favier *et al*., 2018, reported that spontaneous PAC-1 binding was seen in the index case from a family having the αIIb: p.Arg1026Trp variant, but not in the index case from a second family having the same defect, nor in the index case of another family having the β3: p.Asp749His [32]. Some of these studies have been supported by transfection experiments. In accordance, Kunishima *et al*., 2011, have shown that integrin αIIb: p.Arg1026Trp/β3-transfected 293T cells (epithelial embryonic kidney cell line) had spontaneous phosphorylation of FAK, a downstream effector of integrin signaling [19] and Ghevaert *et al*. have found increased anti-PAC-1 binding on αIIb: p.Arg1026Trp/β3-transfected CHO cells (epithelial cell line derived from the ovary of the Chinese hamster) [31].

In our study, none of the two patients having the αIIb: p.Arg1026Trp variant (F1 and F2) had evidence for constitutive PLT activation as evaluated by PAC-1 and bFG expression on the PLT surface at the basal state; unfortunately, patients from the only family having the β3: p.Asp749His were not available for PLT activation studies. Transfection studies were not performed as they are not available in our center.

Discrepancies between studies can be explained by different experimental conditions used to evaluate PLT activation. For instance, Ghevaert *et al*. (β3: p.Asp749His) used a whole blood FCM assay, anti-PCA-1 and anti-FG (instead of an anti-bFG) mAbs [31]; Kunishima *et al*. (αIIb: p.Arg1026Trp) presented the results of only two independent experiments of only one patient with αIIb: p.Arg1026Trp variant, they performed the activation tests in washed PLTs suspended in Tyrode´s buffer; and they used an anti-PAC-1 mAb and FITC-conjugated FG (instead of an anti-bFG mAb) [19]. We tested several patients from several families running in parallel two normal blood samples per day, we performed tests in whole blood, without washes and with minimal sample manipulation in order to avoid artefactual PLT activation, and we evaluated simultaneously two activation related markers (PAC-1 and bFG).

### Platelet activation in response to stimuli

In healthy controls, the median values of PAI, as measured with anti-PAC-1, increased from 5 to 136 and from 6 to 133, after stimulation with TRAP-6 and ADP, respectively (**Table 2**). In addition, soluble FG efficiently binds to its receptor, as demonstrated by a correspondent increase in bFG (median values of PAI increasing from “0” to 16 and from “0” to 19, after stimulation with TRAP-6 and ADP, respectively) (**Table 2**). The only GT patient studied (as a negative control) had no evidence of PLT activation at all (PAI = 0–1, in all conditions tested).

Comparatively to controls, patients with GTLS had a marked impairment of expression of AIBS (PAC-1) on their PLTs after stimulation with TRAP-6 and ADP, having median PAI values of 35 (p<0.001) and 73 (p<0.01), respectively (**Table 2**) (**Fig 2X and 2Y**). Also, compared to controls, GTLS patients had a clear impairment of expression of RIBS (bFG) on their PLTs after stimulation with both PLT agonists, having median PAI values of 3 (p<0.001) and 7 (p<0.01), respectively (**Table 2**) (**Fig 2Z and 2AA**).

Expressing the results obtained in PLT activation studies as a percentage of the normal values, the median values of expression of PAC-1 epitopes on the surface of the PLTs from GTLS patients were of 32% and 22% of the normal, after stimulation with TRAP-6 (p<0.001) (**Table 2**) (**Fig 2A and 2B**), and of 62% and 43% after stimulation with ADP (p<0.01) (**Table 2**) (**Fig 2A and 2C**).

Thus, in general, the PLTs from GTLS patients were hyporeactive to PLT agonists, with PLT activation in response to TRAP-6 being more compromised than PLT activation in response to ADP. Differences between TRAP-6 and ADP can be due to may be related to the different potency of these agonists and their diverse mechanism of action [46] [47].

Patients from F7-F10, who had less severe thrombocytopenia and higher levels of GPIIb/IIIa expression, have somewhat less compromised PLT activation in response to TRAP-6, when compared to patients from F1-F6 (median PAI values: 37 and 24 and median percentages of the normal values: 36% and 23%, respectively). Similar results were obtained for bFG (median PAI values of 3 and 2, median percentages of the normal values of 22 and 20%, respectively) (**Table 2**). The results obtained with ADP were in line with those obtained with TRAP-6, both for PAC-1 (median PAI values: 88 and 65 and median percentages of the normal values: 64 and 60%, respectively) and for bFG (median PAI values: 14 and 6 and median percentages of the normal values: 81 and 43%, respectively). However, these differences did not reach statistical significance, which can be due to the low number of patients studied (6 patients from families F1-F6 and 3 patients from F7-F10).

### Genetic studies

Genetic studies were performed in 34/44 individuals (28/33 patients and 6/11 healthy relatives) from 10 families with GTLS, leading to the identification of *ITGA2B* or *ITGB3* heterozygous variants in 27 patients and excluding their presence in 6 healthy relatives (**Table 3**). Genetic analysis of *ITGA2B* and *ITGB3* genes was performed by NGS in selected patients from families with novel variants, using a commercially available gene panel for hematologic diseases (**S2 File**), excluding potentially pathogenic variants in 394 genes that have been implicated in PLT defects and other hematological disorders.

A total of 7 heterozygous variants were identified, 3 in *ITGA2B* (F1-F5) and 4 in *ITGB3* (F6-F10) (**Table 4**). Three of these genetic defects (in F1-F3, F4 and F10) were previously reported in the literature [15,19,31]. The remaining are novel and were classified as clinically relevant based on low MAF (absent in variant databases), predictions with bioinformatics algorithms and disease co-segregation analysis.

### Variants affecting the αIIb: p.Arg1026 / β3: p.Asp749 salt bridge

In the resting state, the αIIb helix of the αIIbβ3 complex is approximately perpendicular to the membrane whereas the β3 helix is tilted [48]. The dimer is stabilized by two interactions: an OMC, which involves glycine-mediated TM helix packing, and an IMC, which includes packing between αIIb Phe1023/Phe1024 and the two TM helices as well as a salt bridge between the highly conserved cytoplasmatic residues, αIIb: p.Arg1026 and β3: p.Asp749 [1,48,49]. This salt bridge, in which both residues point to each other, confer stability on the inactive state of integrin [49]. The binding of talin to the β3 cytoplasmic domain disrupts this interaction [50].

Until now, **three heterozygous αIIbβ3 variants that interfere with the αIIb: p.Arg1026 / β3: p.Asp749 salt bridge** have been identified—two in *ITGA2B* and one in *ITGB3*. These variants lead to the amino acid substitutions p.Arg1026Gln [15] and p.Arg1026Trp [19,32] in αIIb, and p.Asp749His in β3 [31,32], disrupting the salt link, and theoretically promoting integrin activation. Five of our families had αIIbβ3 variants that disrupt the αIIb: p.Arg1026 / β3: p.Asp749 salt bridge, four in *ITGA2B* (F1-4) and one in *ITGB3* (F10).

**Families 1, 2 and 3** share the **αIIb: p.Arg1026Trp** variant with four previously described Japanese families (11 patients) (19) and two recently reported French families (11 patients) (32). Patients from F1-F3 (twelve in total) have moderate bleeding tendency (median BS: 7), moderate thrombocytopenia (median PLT count: 76x10^9^/L), PLT macrocytosis and anisocytosis (median MPV and PDW: 13fL and 19%, respectively), increased IPF values (median of 14%), decreased expression of αIIbβ3 and β3 (median values: 45% and 50%, respectively), and diminished PLT aggregation and ATP release in response to physiological agonists. These results were similar to those described for the Japanese patients having the αIIb: p.Arg1026Trp variant [19]. Compatible results were also described in the French patients having the same variant [32]. Platelet activation studies evaluated by FCM in two representative patients from F1-F3 revealed a marked impairment of expression of AIBS (PAC-1) epitopes on αIIbβ3 and RIBS epitopes on bFG (9F9), reaching less than 15% of the normal values after stimulation with TRAP-6 and around 50% after stimulation with ADP. Again, these results are comparable to those obtained in both the Japanese and the French patients [19,32]. Concerning the evidence of spontaneous (basal) PLT activation, however, the results are not consistent. In our study, neither of the two patients studied from F1-F3 had evidence for constitutive PLT activation (**S5 File**). In contrast, in the Japanese series, the only patient studied showed spontaneous PAC-1 and FG binding to resting PLTs, suggesting that the mutated integrin is partially activated [19]. In the French series, however, Favier *et al*. observed spontaneous PAC-1 and FG binding in only one of the two index cases studied [32].

**Family 4** had the **αIIb: p.Arg1026Gln** variant, a genetic defect identified previously in an Italian boy [15], the first patient described in the literature [14]. The characteristics of the single patient studied in this family overlap with those described previously. As was seen for F1-F3, this patient also had a compromised PLT response to simulation with TRAP-6 and ADP, without evidence of constitutive PLT activation. This family had however a slightly less severe phenotype as compared with the families with the p.Arg1026Trp variant. A possible explanation may reside in the fact that the wild-type residue at position 1026 of αIIb (an arginine) is basic, where substitution by a neutral residue (glutamine) would exert a less detrimental effect than a hydrophobic residue (tryptophan) [16]. Thus, both αIIb variants (p.Arg1026Trp and p.Arg1026Gln) may disrupt the salt bridge interaction to different extent.

**Family 10** shares the **β3: p.Asp749His** variant with two European families and, like the first family described [31], had no bleeding symptoms. The studied individuals had mild thrombocytopenia (median PLT count: 128x10^9^/L), PLT macrocytosis and anisocytosis, increased IPF, and slightly decreased expression of αIIbβ3 integrin (around 70% of the normal)—between the results from the UK family with normal αIIbβ3 expression [31], and the results from the French family with intermediate αIIbβ3 expression [32]. As the index case was medicated with antiplatelet drugs, and his daughter was not available for functional studies, we were not able to perform PLT aggregation and activation studies in this family.

### Variants affecting the transmembrane domains of αIIbβ3

Previous studies have shown that activating variants of integrin αIIbβ3 are also found in TMDs [50]. Most of the activating variants are substitutions of Gly resides in both TMDs of αIIb (p.Gly1003, p.Gly1007) and β3 (p.Gly734) with amino acids containing bulky side chain, such as Leu, Ile, or Asn [51]. Two Gly in αIIb TMD are part of a GXXXG motif that plays an important role in TMD helix-helix association [51]. The glycine residues at αIIb p.Gly1003/Gly1007 and β3 p.Gly734 allow close inter-helical packing in the N-terminal half of the TMD and crossing of the αIIb and β3 TMCD helices at an angle of approximately 30 degrees [52]. It has been suggested that not only the inner membrane interaction involving αIIb (p.Phe1023, p.Phe1024 and p.Arg1026) and β3 (p.Asp749), but also the outer membrane interaction (including the αIIb GXXXG motif), decrease the TMD-tail association, being required for stabilization of the TMD interface and the inactive state of integrin [50].

**Family 5** has a **new heterozygous variant** (**αIIb: p.Gly1007Val**) **at the TMD of αIIb**. This family shares a similar phenotype with the families with variants affecting the p.Arg1026 residue: moderate bleeding (median BS: 5), mild thrombocytopenia (median PLT count: 93x10^9^/L), PLT macrocytosis and anisocytosis, increased IPF values and decreased expression of αIIbβ3 (around half of the normal). However, F5 has a markedly lower PLT aggregation in response to all the evaluated agonists than F1, F3 and F4, and it was the only family having impaired agglutination with ristocetin. This variant compromises αIIbβ3 activation induced by TRAP-6 and ADP, as evaluated by the exposition of AIBS (PAC-1) and RIBS (bFG) epitopes, at levels that are somewhat less severe as compared to those observed with variants that might interfere with the αIIb: p.Arg1026 / β3: p.Asp749 salt bridge. Once again, we found no evidence of constitutive PLT activation in two representative patients from this family, as evaluated by FCM.

### Variants affecting the transmembrane and cytoplasmic tail border

At the **transmembrane and cytoplasmic tail border**, it is conceivable that the side chains of the membrane-proximal residues p.Val1021-p.Phe1024, and p.Leu743-p.Ile747, part of the conserved domains GFFKR of αIIb and LLITIHD of β3 are inserted into or anchored to the membrane, which may stabilize the relative orientations of the TM helices [52]. The conformation of these mostly hydrophobic residues will control the TM helix tilt and, consequently, the default orientation at which αIIb and β3 will face each other [53].

**Family 6** has a new heterozygous variant at the transmembrane and cytoplasmic tail border of β3 (**β3: p.Thr746Pro**) converting a threonine (polar) onto a proline (non-polar) residue; one may speculate whether this polarity change affects the orientation of the TM helix tilt. This family shares a similar phenotype with the previous families described: moderate bleeding (median BS: 7), moderate thrombocytopenia (median PLT count: 69x10^9^/L), PLT macrocytosis (median MPV: 15fL) and anisocytosis (median PDW: 22%), increased IPF (median value: 20%), and decreased αIIbβ3 expression (median value: 39%). However, the PLT aggregation was worse (absent or almost absent with all the agonists), more resembling that observed in GT. Apparently, this variant strongly compromises αIIbβ3 activation induced by TRAP-6 and ADP, as measured by the expression of PAC-1 and bFG epitopes, compared to that observed for the variants that interfere with the αIIb: p.Arg1026 / β3: p.Asp749 salt bridge. Here again, there was no evidence of constitutive PLT activation in the study of one patient representative of this family.

### Variants affecting the membrane-proximal cytoplasmic domains

**Talin,** a cytoskeletal protein, activates the integrin through a **direct interaction between its F3 domain and the β integrin tail** [54]. This disrupts the αIIb/β3 interaction, causing tail separation, and promoting a more open and extended extracellular conformation of the αIIb/β3 integrin, which binds extracellular ligands much more tightly [2]. It was suggested that variants that affect the residues p.His748-p.Asp749 or p.Asp749-p.Arg750 substantially reduce talin binding to β3 [55,56], which likely disrupts the inhibitory interaction of the β integrin tail with the conserved p.Arg1026 residue in αIIb, thereby contributing to integrin activation [57].

**Family 7** has a missense variant in the membrane-proximal cytoplasmic domains of β3, resulting in a histidine (basic) to proline (non-polar) substitution (**β3: p.His748Pro**). This variant causes a phenotype of intermediate severity. Both patients studied (sisters) had mild to moderate bleeding (median BS = 4–5), mild thrombocytopenia (median PLT count: 120x10^9^/L), PLT macrocytosis (median MPV: 16fL) and anisocytosis (20%, one patient), increased IPF (median value: 27%), decreased αIIbβ3 expression (median value: 64%) and diminished PLT aggregation in response to physiological agonists. This family also had a compromised PLT response upon to simulation with TRAP-6 and ADP, without evidence of constitutive PLT activation.

### Variants affecting the membrane distal cytoplasmic domains

A **salt bridge interaction between the most membrane distal regions of the αIIb and β3 integrin subunits with residues p.Glu1037 and p.Arg760** respectively was previously described [3]. Talin is known to also disrupt this bridge, through the initial membrane-distal interactions that anchor this cytoskeletal protein to the αIIbβ3 integrin [3].

**Families 8 and 9** share a heterozygous variant affecting the membrane distal cytoplasmic domains of β3, leading to arginine to cysteine substitution (**β3: p.Arg760Cys**). These families have distinct characteristics consisting of: i) absent to mild bleeding (median BS = 3); ii) mild thrombocytopenia (median PLT count: 115x10^9^/L), with some patients even having normal PLT counts; iii) mild PLT macrocytosis (median MPV: 13 fL) and anisocytosis (median PDW: 17%); and iv) only slightly increased IPF (median value: 9%). In addition, PLT aggregations in response to physiological agonists are diminished but to a lesser extent than in F1-F6. Moreover, the levels of αIIbβ3 expression (median value: 77%) proximate to the lower limits of the normal values. PLT activation in response to PAC1 and ADP was less compromised than in other families, with F8 having normal activation with ADP. Interestingly, these were the only families in which we found evidence of constitutive PLT activation.

### Clinical relevance and pathogenicity of the new GTLS variants

Almost all the previously reported heterozygous variants cluster in a small region at the membrane-proximal domains of αIIbβ3 (amino acids 1022–1026 for αIIb and 744–749 for β3), the IMC, affecting the salt bridge between αIIb: p.Arg1026 and β3: p.Asp749. As in the previously reported cases, the genetic variants identified in our families localize mostly in TM-cytoplasmic domains of αIIbβ3. Five of our families had three previously described variants: αIIb (p.Arg1026Trp), αIIb (p.Arg1026Gln), and β3 (p.Asp749His), affecting the αIIb: p.Arg1026 / β3: p.Asp749 salt bridge. The remaining variants found in our patients, one in *ITGA2B* (F5) and three in *ITGB3* (F6-F9) are new, and they involve the TMD of αIIb (p.Gly1007Val), the TM and cytoplasmic tail border (p.Thr746Pro), and the membrane-proximal (p.His748Pro) and distal (p.Arg760Cys) cytoplasmic domains of β3.

The novel variants were confirmed as clinically relevant based on low MAF (absent in variant databases), predictions with bioinformatics algorithms and disease co-segregation analysis. According to the joint consensus recommendations of the American College of Medical Genetics and Genomics (ACMG) and the Association for Molecular Pathology (AMP) [58], three variants were classified as pathogenic– c.3076C>T (F1, F2 and F3) and c.3077G>A (F4) at *ITGA2B*, and c.2245G>C (F10) at *ITGB3 –*, one variant was classified as likely pathogenic– c.2278C>T at *ITGB3* (F8 and F9)–, and three variants were classified as having unknown significance (VUS)– c.3020G>T (F5) at *ITGA2B*, c.2236A>C (F6) and c.2243A>C (F7) at *ITGB3* (**Table 4**).

Although no transfection studies were performed, the pathogenicity of the novel variants is also supported by previous structural and molecular functional studies of integrin αIIbβ3. These studies have indicated major interactions between the transmembrane and cytoplasmatic domains of the *α*- and *β*-subunits of αIIbβ3 resulting in an inactive state of integrin. One of these interactions, confirmed by mutational studies, is represented by the TMD helix-helix association allowed by the glycine residues at αIIb p.Gly1003/Gly1007 and β3 p.Gly734 (outer membrane clasp); one of the novel variants found in our patients (αIIb: p.Gly1007Val) is a glycine substitution at αIIb p.Gly1007, interfering with inter-helical packing of the αIIb and β3 TMD. Another described interaction is a salt bridge between residues p.Glu1037 and p.Arg760 of the membrane distal cytoplasmatic domains of the αIIb and β3 integrin subunits, respectively; a novel variant found in this series (β3: p.Arg760Cys) may disrupt this interaction. Lastly, two novel variants are part of the conserved domain LLITIHD of β3: the first variant (β3: p.Thr746Pro), part of the p.Leu743-p.Ile747 residues, may stabilize the relative orientations of the TM helices; the second variant (β3: p.His748Pro) likely disrupts the αIIb/β3 inhibitory interaction between the αIIb: p.Arg1026 and β3: p.Asp749 by reducing talin binding to β3.

### Phenotypic variability and phenotype-genotype correlations

Notwithstanding some variability, our study reveals two different phenotypic patterns in GTLS.

The first and more severe, is characterized by moderate bleeding tendency, moderate macrothrombocytopenia, PLT anisocytosis, impaired PLT aggregation, intermediate levels of αIIbβ3 integrin and compromised PLT response to in vitro stimulation. This pattern is present in families F1-F6 in which variants are present in αIIb p.Arg1026 affecting the αIIb: p.Arg1026/β3: p.Asp749 salt bridge (four families), in αIIb p.Gly1007 interfering with inter-helical packing of the αIIb and β3 TMD (one family) and in β3 p.Thr746 stabilizing the relative orientations of the TM helices (one family).

The second and more “benign” phenotypic pattern is characterized by absent to mild bleeding, mild macrothrombocytopenia or even normal PLT counts, marginally diminished levels of αIIbβ3, and less compromised PLT aggregation and activation. The families F7-F10 with this milder pattern, have variants in β3: p.Asp749 and in near‐neighbor β3: p.His748 disrupting the αIIb/β3 interaction between the αIIb: p.Arg1026 and β3: p.Asp749, and in β3: p.Arg760, affecting another interaction between residues p.Glu1006 and p.Arg760 in the most membrane distal regions of the αIIb and β3 integrin subunits, respectively. Notice that our families with the β3: p.His748Pro/p.Asp749His had a similar profile to that of the first reported family with the variant β3: p.Asp749His, called a non‐synonymous single nucleotide polymorphism (SNP) by the authors [31].

It should be noted that all the cases presented here (including the families with a more “benign” phenotype, F7 to F10) share with the GTLS families previously reported by others, the same phenotype with a variable bleeding tendency, as well as variable grades of macrothrombocytopenia, platelet anisocytosis, low αIIbβ3 expression, impaired platelet aggregation, compromised PLT activation and, at least in some cases, evidence for constitutive αIIbβ3 activation. Variability in the platelet counts was also found in other previously published GTLS cases, including the first described family in which the platelet count of the proband varied between 100 and 160 x 10^9^/L [14]. Noteworthy, in our study, families F8 and F9, β3: p.Arg760Cys, whose PLT counts overlap with the normal ranges, were the only ones that in our series have evidence for constitutive PLT activation.

Discrepancies between studies concerning constitutive αIIbβ3 activation in patients with GTLS (ITGA2B/ITGB3-RT) are probably a reflection of different experimental conditions, indicating that FCM-based tests urgently need to be standardized to provide reproducible results in clinical settings. Unfortunately, although several efforts have been made in the last years to normalize FCM-based functional studies and some of the variables affecting the results have already been identified, there is yet no agreement on this subject [33–36]. As such, can we continue to assume GTLS (ITGA2B/ITGB3-RT) to be a disease that invariably causes constitutive activation of the GPIIb/IIIa receptor? In fact, rather than conferring a gain-of-function, GTLS-associated variants are invariably hypomorphic, i.e., variants in which the altered gene product possesses a reduced level of activity, or in which the wild-type gene product is expressed at a reduced level. In accordance, the genetic variants associated with GTLS invariably translate into decreased αIIbβ3 expression on the PLT membrane, as well as decreased PLT reactivity in response to several agonists, as measured by PFA, PLT aggregometry / ATP release and FCM-based PLT activation studies.

### Screening for GTLS in patients with chronic macrothrombocytopenia

Our study emphasizes the need of defining rational algorithms for phenotypic, functional and genetic investigation of the inherited PLT disorders, as already proposed [59]. The high number of families with GTLS (ITGA2B/ITGB3-RT) found in our Center is probably because, after discovering the first case in 1995, we have systematically screened for αIIbβ3 deficiency in patients with familial macrothrombocytopenia or with unexplained chronic macrothrombocytopenia, searching for possible pathogenic variants in *ITGA2B* or *ITGB3* genes in all cases with macrothrombocytopenia and decreased expression of αIIbβ3. Our study favors the idea that screening mutations by NGS alone (without performing phenotypic studies) may not be the best approach, and the relatively high number of families with distinct identified variants would suggest that the frequency of GTLS may be higher than previously thought, but still rare as none of these variants were identified in WES/WGS databases [23]. According to our experience, besides patients with familial macrothrombocytopenia, other patients that may benefit from GTLS screening are those with atypical “immune thrombocytopenia” (e.g., F7) or “chronic idiopathic macrothrombocytopenia” (e.g. F8, F10), even when a familial history is not obvious (as many patients are unaware of a family history of thrombocytopenia). Patients with “gestational thrombocytopenia” (e.g. F2, F8, F9) may also benefit from GTLS screening because thrombocytopenia detected during pregnancy is a relatively common reason for consulting the hematologist, and distinguishing GTLS from gestational thrombocytopenia, immune thrombocytopenia, preeclampsia, HELLP syndrome, or thrombotic thrombocytopenic purpura is essential since the treatment differs widely [60]. It is worth mentioning that in patients with GTLS, thrombocytopenia can worsen or event manifest only in special periods of life where there are additional thrombopoietic demands (e.g. pregnancy, intercurrent infections, surgeries), as it happens in some of our families. This is probably because, as previously mentioned, αIIbβ3 is involved in proplatelet formation and release of PLTs from proplatelet tips [10,11]. The importance of a correct diagnosis is unquestionable because some patients with GTLS had been investigated for a long time due to chronic thrombocytopenia, and some had been misdiagnosed (e.g. autoimmune thrombocytopenia, gestational thrombocytopenia), with therapeutic implications (e.g. corticosteroids and PLT transfusions).

## Conclusions

Our study details the phenotypic and functional defects observed in GTLS, confirms and expands the number and location of the associated αIIbβ3 variants, establish possible phenotype/genotype correlations, and reinforces the need of an adequate strategy for the diagnosis of this rare entity. To the best of our knowledge, this is the largest cohort of patients with GTLS (ITGA2B/ITGB3-RT) described by a single center, and one of the best characterized from the clinical and laboratory point of view.

## Supporting information

S1 File Flow cytometry protocols for platelet glycoprotein quantification and activation studies. (DOCX)

S2 File Next generation sequencing panel and primers used for gene sequencing. (DOCX)

S3 File Clinical and laboratory data from the study population. (DOCX)

S4 File Platelet function and lumiaggregometry assays. (DOCX)

S5 File Flow cytometry based platelet activation studies. (DOCX)

## Abbreviations

AA: arachidonic acid
ACMG: American College of Medical Genetics and Genomics
AD: autosomal dominant
ADP: adenosine diphosphate
AIBS: activation-induced binding sites (in GPIIb/IIIa)
AMP: Association for Molecular Pathology
AR: autosomal recessive
ATP: adenosine triphosphate
ATP-R: ATP release
BAS: basal state
BAT: bleeding assessment tool
BC: Beckman Coulter, Hialeah, FL, USA
BD: Becton Dickinson, San Jose, CA, USA
bFG: bound fibrinogen
BS: bleeding score
C: control
CHUP: Centro Hospitalar Universitário do Porto
CLC: Chrono-Log Corporation, Havertown, PA, USA
COL: collagen
COL/ADP: collagen/adenosine diphosphate
COL/EPI: collagen/epinephrine
CTD: C-terminal cytosolic domain
CTD: cytosolic domain
DK: Dako, Glostrup, Denmark
DNA: deoxyribonucleic acid
ECD: extracellular domain
ECM: extracellular matrix
EDTA: ethylene diamine tetracetic acid
EPI: epinephrine
F: female
FCM: flow cytometry
FG: fibrinogen
FITC: fluorescein isothiocyanate
FSC: forward scatter
GP: glycoprotein
GT: Glanzmann´s Thrombasthenia
GTLS: Glanzmann´s Thrombasthenia-like syndrome
IMC: inner membrane clasp
IPF: immature platelet fraction (%)
ISTH: International Society on Thrombosis and Hemostasis
ISTH-BAT: International Society on Thrombosis and Hemostasis–Bleeding Assessment Tool
ITGA2B: integrin alpha 2b gene
ITGA2B/ITGB3*-*RT: ITGA2B/ITGB3-related thrombocytopenia
ITGB3: integrin beta 3 gene
LIBS: ligand induced binding sites (on the GPIIb/IIIa receptor)
M: male
mAb: monoclonal antibody
MAF: minor allele frequency
MFI: median fluorescence intensity
MPV: mean platelet volume (fL)
NA: not available
NGS: next generation sequencing
OMC: outer membrane clasp
PAGL: platelet agglutination
PAGR: platelet aggregation
PAI: platelet activation index
PAI/ADP/bFG: PAI upon stimulation with ADP, as evaluated with anti-bFG mAb
PAI/ADP/PAC-1: PAI upon stimulation with ADP, as evaluated with anti-PAC-1 mAb
PAI/BAS/bFG: PAI at basal conditions, as evaluated with anti-bFG mAb
PAI/BAS/PAC-1: PAI at basal conditions, as evaluated with anti-PAC-1 mAb
PAI/TRAP-6/FG: PAI upon stimulation with TRAP-6, as evaluated with anti-bFG mAb
PAI/TRAP-6/PAC-1: PAI upon stimulation with TRAP-6, as evaluated with anti-PAC-1 mAb
PB: peripheral blood
PDW: platelet volume distribution width (%)
PE: phycoerythrin
PFA: platelet function assay
PLT: platelet
PPP: platelet poor plasma
PRP: platelet rich plasma
RBC: red blood cells
RIBS: receptor induced binding sites (on the fibrinogen ligand)
RIST: ristocetin
RNA: ribonucleic acid
SD: standard deviation
SNP: single nucleotide polymorphism
SSC: side scatter
TFS: Thermo Fisher Scientific
TM: transmembrane
TMD: transmembrane domain
TRAP-6: Thrombin Receptor Agonist Peptide-6
VUS: variant of unknown significance
