# Supplementary material for: αIIbβ3 variants in ten families with autosomal dominant macrothrombocytopenia: Expanding the mutational and clinical spectrum
Source: PLoS One. 2020 Dec 4;15(12):e0235136. doi: 10.1371/journal.pone.0235136 (PMC7717987; doi:10.1371/journal.pone.0235136)
Supplement: S1 File — (DOCX) [file pone.0235136.s001.docx]

Title**: αIIbβ3 variants in ten families with autosomal dominant macrothrombocytopenia: expanding the mutational and clinical spectrum**

Short title: **Familial macrothrombocytopenia with αIIbβ3 integrin deficiency**

**AUTHORS:** Sara Morais, Jorge Oliveira, Catarina Lau, Mónica Pereira, Marta Gonçalves, Catarina Monteiro, Ana Rita Gonçalves, Rui Matos, Marco Sampaio, Eugénia Cruz, Inês Freitas, Rosário Santos, Margarida Lima

**SUPPLENTARY FILE 1 (S1 FILE) HOSTED IN ZENODO:** http://doi.org/10.5281/zenodo.3660449

## S1 FILE | METHODS: FLOW CYTOMETRY PROTOCOLS FOR THE STUDY OF PATIENTS SUSPECTED OF HAVING GTLS / ITGA2B/ITGB3-RELATED THROMBOCYTOPENIA

### Background

Pathogenic variants in *ITGA2B* and *ITGB3*, the genes coding for the αIIb and β3 – platelet (PLT) glycoproteins (GP) IIb and IIIa, respectively, give rise to Glanzmann´s Thrombasthenia (GT), a rare autosomal recessive bleeding disorder due to quantitative or qualitative defects of integrin αIIbβ3 (GPIIb/IIIa complex) characterized by normal PLT counts and volume, and absent PLT aggregation ([Nurden AT, 2013](https://www.ncbi.nlm.nih.gov/pubmed/?term=23929305)) ([Nurden AT, 2018](https://www.ncbi.nlm.nih.gov/pubmed/?term=29125375)). A limited number of patients, however, have been reported to have autosomal dominant variants of GT associated with macrothrombocytopenia, PLT anisocytosis, PLT function defects and mild to moderate hemorrhage, in association with pathogenic variants in the *ITGA2B* or *ITGB3* genes. These cases have deserved the designation of GT-like syndrome (GTLS) or ITGA2B/ITGB3-related thrombocytopenia (ITGA2B/ITGB3-RT) ([Nurden AT, 2011](https://www.ncbi.nlm.nih.gov/pubmed/?term=22102273)) ([Kunishima S, 2011](https://www.ncbi.nlm.nih.gov/pubmed/?term=21454453)) ([Bury L, 2016](https://www.ncbi.nlm.nih.gov/pubmed/?term=26452979)) ([Favier M, 2018](https://www.ncbi.nlm.nih.gov/pubmed/?term=29090484)) ([Miyashita N, 2018](https://www.ncbi.nlm.nih.gov/pubmed/?term=29380037)).

The genetic variants associated with GTLS translate into decreased αIIbβ3 expression on the PLT membrane, as well as decreased PLT reactivity in response to several agonists, as measured by PLT function assays, PLT aggregometry / ATP release and flow cytometry (FCM)-based PLT activation studies. Macrothrombocytopenia observed in these patients has been attributed to cytoskeletal perturbations and abnormal proplatelet formation due to constitutive PLT activation ([Nurden AT, 2011](https://www.ncbi.nlm.nih.gov/pubmed/?term=22102273)) ([Kunishima S, 2011](https://www.ncbi.nlm.nih.gov/pubmed/?term=21454453)) ([Bury L, 2016](https://www.ncbi.nlm.nih.gov/pubmed/?term=26452979)) ([Favier M, 2018](https://www.ncbi.nlm.nih.gov/pubmed/?term=29090484)) ([Miyashita N, 2018](https://www.ncbi.nlm.nih.gov/pubmed/?term=29380037)). There are, however, discrepancies between studies regarding the levels of PLT activation obtained upon stimulations with different agonists, but also concerning the evidence of constitutive αIIbβ3 activation, which are probably a reflection of different experimental conditions. This indicates that FCM-based tests used for diagnosis of GTLS need to be standardized to provide reproducible results in clinical settings.

Herein we describe in detail in-house developed FCM-based methods used in our Laboratory for PLT glycoprotein evaluation and PLT activation studies in patients suspected of having GTLS / ITGA2B/ITGB3-RT.

**Key words:** Flow cytometry; Inherited platelet disorders; Thrombocytopenia; Platelet dysfunction; Platelet activation; Platelet glycoproteins; Glanzmann´s Thrombasthenia Like Syndrome; ITGA2B/ITGB3-related thrombocytopenia; GPIIb/IIIa; integrin αIIbβ3; ITGA2B; ITGB3

### SAMPLE COLLECTION AND STORAGE

Peripheral blood (PB) samples used for FCM studies are collected aseptically from an antecubital vein using a 21-gauge needle (0.8 x 32 mm) and a light tourniquet, into vacuum tubes containing sodium citrate 3.2% (1 volume trisodium citrate 0.105 M to 9 volumes of blood) (BD Vacutainer System) as anticoagulant.

The first 2-4 ml of PB collected are discarded.

Sample processing is initiated after resting for 1 hour at room temperature and completed within 4 hours after blood collection.

Blood samples are maintained at room temperature before and during sample processing.

All samples with evidence of hemolysis or blood clotting are be discarded.

Patients and controls are asked to stop all the medications that could potentially interfere with platelet function for at least one week before blood collection.

*Comments*

*Pre-analytic critical steps have been debated in previous studies, from the 90´s to the present, and include the procedures of blood collection, choice of the anticoagulant, conditions of sample storage, and time to sample processing, among others (*[*Michelson AD, 1996*](https://www.ncbi.nlm.nih.gov/pubmed/?term=8652804)*) ([Mody M, 1999](https://www.ncbi.nlm.nih.gov/pubmed/?term=10354385)) ([Huskens D, 2018](https://www.ncbi.nlm.nih.gov/pubmed/?term=29389990)) (*[*Pedersen OH,2018*](https://www.ncbi.nlm.nih.gov/pubmed/?term=28960147)*) (*[*van Asten I, 2018*](https://www.ncbi.nlm.nih.gov/pubmed/?term=29337406)*) (*[*van Asten I, 2018*](https://www.ncbi.nlm.nih.gov/pubmed/?term=29554715)*).*

### EQUIPMENT – FLOW CYTOMETER

We routinely use a Navios™ flow cytometer from Beckman Coulter (BC, Hialeah, FL, USA). However, these protocols can also be used with FACSCanto II from Becton Dickinson (BD, San Jose, CA, USA).

Flow cytometer set-up and calibration is performed accordingly to the Euroflow consortium SOAPs (*update versions available at the EuroFlow website -* [*http://euroflow.org/*](http://euroflow.org/)).

Daily quality controls are made to guarantee the quality of the study, including fluorescence standardization, linearity assessment and spectral compensation.

Eight-peak Rainbow bead calibration particles (Spherotech, Lake Forest, IL, USA) are used for initial PMT settings, as well as for daily checks.

Daily control is monitored by plotting fluorescence intensity values in Levy Jennings charts.

In addition, external quality assessment / proficiency testing is performed by participating in the Euroflow QA program.

*Comments*

*Local laboratories should ensure proper calibration of the equipment used, especially the flow cytometers, as well as their stability over the time, using validated Standard Operating Procedures (SOAPs), performing appropriate internal quality controls (IQC) and participating in external quality control / assessment programs (EQC). We recommend to use the SOAPs for instrument setup developed by Euroflow ([Kalina T, 2012](https://www.ncbi.nlm.nih.gov/pubmed/?term=22948490)), which are fully standardized and have been validated across different Beckman Coulter and Becton Dickinson platforms, allowing for comparable fluorescence measurements for at least three flow cytometers (Navios, FACSCanto II and FACSLyric) and enabling cross-platform inter- and intra-laboratory standardization ([Glier H, 2019](https://www.ncbi.nlm.nih.gov/pubmed/?term=31655051)). Taking in consideration that external Quality Assurance (QA) programs specific for platelet studies are not yet available, we suggest the participation in the Euroflow-QA program ([Kalina T, 2012](https://www.ncbi.nlm.nih.gov/pubmed/?term=25345353)).*

### S1 | PROTOCOL 1: FLOW CYTOMETRY PROTOCOL FOR PLATELET GLYCOPROTEIN EVALUATION

#### Brief explanation

Surface expression of platelet (PLT) glycoproteins is measured in whole peripheral blood (PB) by flow cytometry (FCM).

At least three (ideally four) PB samples obtained from healthy individuals (blood donors) are studied daily, in parallel with patients´ samples, as normal controls.

#### Duration

Approximately 30 minutes.

#### Temperature

Reagents and samples should be kept at room temperature when in use and samples should be processed at room temperature (approximately 22ºC).

#### Peripheral blood samples

- PB samples collected into sodium citrate containing tubes.
  - Peripheral blood (PB) sample from the patient.
  - PB samples from at least 3 (ideally 4) healthy controls to be processed in parallel.
- Leave the PB samples resting for 60 minutes at room temperature after collection until study begins.

#### Reagents

##### Monoclonal antibodies (mAbs)

PLT staining is performed using fluorescein isothiocyanate (FITC) and phycoerythrin (PE) -conjugated mouse anti-human mAbs from Beckman Coulter (BC, Hialeah, FL, USA) – Immunotech (IOT), Becton Dickinson (BD, San Jose, CA, USA) and from Dako (DK, Glostrup, Denmark), whose catalogue numbers (Ref.) are indicated below.

- PE-conjugated mouse anti-human CD42b (GPIb) (clone AN51; DK, Ref. R7014)
- FITC-conjugated mouse anti-human CD41a (GPIIbIIIa) (clone HIP8; BD, Ref. 333147)
- FITC-conjugated mouse anti-human CD61 (GPIIIa) (clone RUU-PL7F12; BD, Ref. 347407)
- PE-conjugated mouse anti-human CD62P (P-Selectin) (clone CLBThromb/6; BC-IOT, Ref. IM1759)

*Comments*

*Anti-CD61 and anti-CD41a mAbs are specific for the proteins of the GPIIb/IIIa complex: anti-CD61 recognizes the GPIIIa, whereas anti-human CD41a recognizes the calcium dependent GPIIb/IIIa complex. Anti-CD42b is specific for the GPIb glycoprotein. CD41a expression increases after PLT activation ([Matzdorff A, 2006](https://www.ncbi.nlm.nih.gov/pubmed/?term=15894353)), an opposite effect being observed on the levels of CD42b (*[*Michelson AD, 1994*](https://www.ncbi.nlm.nih.gov/pubmed/?term=8204882)*).*

*Basal expression of P-Selectin (CD62P) is used to evaluate artefactual PLT activation due to sample collection, storage or processing.*

*Background staining is evaluated in both cases using FITC- and PE-conjugated isotype mAbs.*

##### Other reagents

- Bovine Serum Albumin (BSA) (Sigma, Ref. A3294-50G)
- Ethylenediaminetetraacetic acid dipotassium salt dihydrate, puriss. p.a., >=99.0% (KT) (EDTA-K2) (Sigma-Aldrich; Ref. SAF-03660-100G).
- Phosphate buffered saline (PBS) (15 packets; 15x500mL) (BC, Ref. 6603369) – Each package contains 15 foil-wrapped packets of PBS Buffer reagent. Each package is brought up to a 500 mL volume with distilled water to yield a 0.01 M potassium phosphate, 0.15 M sodium chloride at a pH of 7.2 ± 0.2.

#### Solutions

- PBS containing BSA 0.2% (w/v) – PBS-BSA(0.2%) – conserved at the refrigerator (-4ºC) and less to stay at room temperature before use.

*Comments*

*Local FCM laboratories should implement IQC programs for the reagents used, especially concerning the mAbs, in order to prevent disparities related to antibody degradation (e.g. improper use after the expiration date, unappropriated storage conditions) and lot-to-lot variations.*

#### Technical protocol

##### Platelet staining

Sample staining is performed by adding 100 µL of PBS-BSA(0.2%), 10 µL of citrated whole blood and 10 µL of the mAb to the corresponding tube, mixing gently, and incubating for 15 minutes in darkness at room temperature. After incubation, cells are suspended with 500 µL of PBS-BSA(0.2%), mixed gently again, and immediately analyzed in the flow cytometer.

##### Sample acquisition

All samples are acquired in a Navios™ flow cytometer (BC). The minimum number of PLT events acquired per tube is of 5,000. All the events acquired are recorded and stored as listmode files (.lmd). Forward scatter (FSC) and side scatter (SSC), represented as FSC and SSC integral, are captured on a logarithmic scale; for fluorescence parameters, a logarithmic amplification is also used.

##### Data analysis

PLTs are discriminated from debris and from other cells and gated based on their light scatter properties, FSC and SSC, and cell surface expression of the PLT-associated GP, and the median fluorescence intensity (MFI) is measured for each platelet GP analyzed.

*Comments*

*For PLT glycoprotein evaluation we use the NAVIOS acquisition/analysis software; however, FCM data analysis may also be done using other compatible software, such as the Infinicyt^TM^ (Cytognos, Salamanca, Spain).*

#### Expression of results

The results are expressed as a percentage of normal (patient MFI/mean control MFI*100) and corrected for PLT size as evaluated by the FSC (**Figure 1**).

| 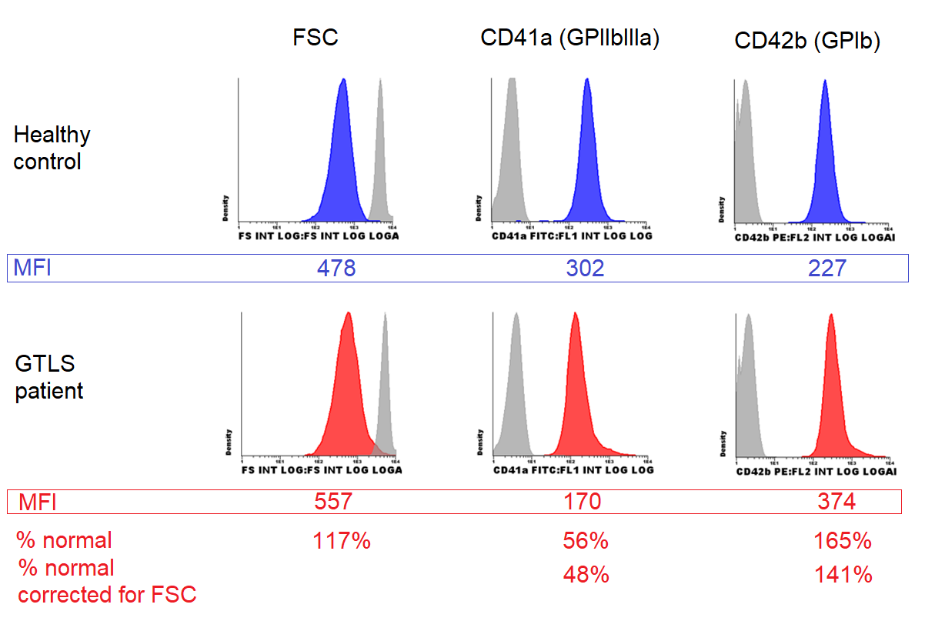 | **Abbreviations**:  GTLS, Glanzmann´s Thrombasthenia like syndrome;  FSC, forward scatter;  MFI, median fluorescence intensity.  **Color code**:  blue, control platelets;  red, patient´s platelets;  gray, leukocytes. |
| --- | --- |

**Figure 1.** Forward scatter (FSC), CD41a (GPIIb/IIIa) and CD42b (GPIb) histograms from a representative healthy control and a representative GTLS patient, as evaluated by flow cytometry.

#### Expected results

##### Normal Reference Values

Normal reference values for PLT glycoproteins with our method and using the mAbs mentioned above are as follows: CD41a (GPIIb/IIIa) (85-115%); CD61 (GPIIIa) (80-120%); CD42b (GPIb) (70-130%). These values were based on the study of about 50 healthy adults (blood donors) whose results, expressed as mean ± standard deviation and [percentile 5 – 95] values were: CD41a (n=56): 100 ± 16% [85-117%]; CD61 (n=52): 100 ± 17% [81-119%]; CD42b (n=52): 100 ± 21% [70-137%].

##### Expected Results in GTLS Patients

GTLS patients are expected to have low levels of CD41a (GPIIb/IIIa) (<85% of the normal values) and CD61 (GPIIIa) (<85% of the normal values), and normal or increased levels of CD42b (GPIb).

#### Protocol validation

This protocol, is being used in our laboratory since 1992 and has been applied to the diagnosis of dozens of patients with PLT defects, including GT, Bernard Soulier syndrome and other rare familial PLT disorders / inherited PLT function defects.

It was initially validated for the study of GTLS in a subset of 11 patients and 8 controls (healthy adult individuals, blood donors), by quantifying platelet GPs in parallel using the commercially available kits from Biocytex (Marseille, France): Platelet Gp Screen (Ref. 7008) and Platelet Gp/Receptors (Ref. 7004) (**Table 1**). Samples were processed according to the manufacture instructions and the results were expressed as numbers of GP molecules per platelet.

**Table 1.** Comparison of the in house developed semi-quantitative method used to evaluate platelet GP expression in patients with GTLS, with commercially available kits for quantification of platelet GP.

|  | Quantitative commercially available kits  (number of GP molecules per platelet) †  (Years: 1995-1998) | | | Semi-quantitative in house developed method ‡  (% of normal GP values) §  (Years: 1995-1998) | | |
| --- | --- | --- | --- | --- | --- | --- |
|  | CD41  (GPIIIa) | CD61  (GPIIIa) | CD42b  (GPIb) | CD41a  (GPIIb/IIIa) | CD61  (GPIIIa) | CD42b  (GPIb) |
| Controls* | N=8  46440 ± 14091 | N=8  54586 ± 12222 | N=8  27057 ± 19828 | N=8  100 ± 18  [80-120] | N=8  100 ± 12  [84-120] | N=8  100 ± 25  [73-134] |
| Patients | N=11  29313 ± 6180 | N=11  32548 ± 5963 | N=11  40626 ± 7660 | N=11  49 ± 6  (41-65) | N=11  60 ± 10  (48-77) | N=11  172 ± 36  (113-225) |
| Unaffected´ relatives | N=1  46056 | N=1  58528 | N=1  25411 | NA | NA | NA |

Abbreviations: GP, glycoprotein.

Results are presented as mean ± one standard deviation, [P5-P95] and (minimum-maximum) values.

* Healthy adult individuals (blood donors)

† Platelet Gp Screen (catalogue number 7008) and Platelet Gp/Receptors (catalogue number 7004) kits (Biocytex, Marseille, France).

‡ Whole blood staining with FITC-conjugated mouse anti-human CD41a (GPIIb/IIIa, clone HIP8) and FITC-conjugated mouse anti-human CD61 (GPIIIa) (clone RUU-PL7F12), both from Becton Dickinson Biosciences, San Jose, CA, USA, and PE-conjugated mouse anti-human CD42b (GPIb) (clone AN51), from Dako, Glostrup, Denmark.

§ For the semi-quantitative method, PLT glycoproteins levels are expressed as percentage of normal GP values, calculated by dividing the MFI obtained in PLT from each sample after staining with fluorochrome-conjugated monoclonal antibodies directed against the correspondent PLT glycoprotein, by the mean MFI obtained in the control group.

### S1 | PROTOCOL 2: FLOW CYTOMETRY PROTOCOL FOR PLATELET ACTIVATION STUDIES

#### Brief explanation

Platelet (PLT) activation is measured in whole peripheral blood (PB) by flow cytometry (FCM) by measuring the expression of activation-induced binding sites (AIBS) in GPIIb/IIIa (anti-PAC1 mAb) and receptor induced binding sites (RIBS) on the fibrinogen (FG) ligand (anti-bFG mAb), after stimulation with ADP (Adenosine Diphosphate) or TRAP-6 (Protease activated receptor (PAR)-1 agonist thrombin receptor activator peptide 6), using an in-house developed protocol, based on a commercially kit that is no longer in the market (PLATELET Fibrinogen, Biocytex, Marseille, France; catalogue number 7012).

Two PB samples obtained from healthy individuals (blood donors) are studied daily, in parallel with patients´ samples, as normal controls.

#### Duration

Approximately 2 hours.

#### Temperature

Reagents and samples should be kept at room temperature when in use and samples should be processed at room temperature (approximately 22ºC).

#### Peripheral blood samples

- PB samples collected into sodium citrate containing tubes.
  - PB sample from the patient.
  - PB samples from 2 healthy controls to be processed in parallel.
- Leave the PB samples resting for 60 minutes at room temperature after collection until study begins.

#### Reagents

##### Monoclonal antibodies (mAbs)

PLT staining is performed using fluorescein isothiocyanate (FITC) and phycoerythrin (PE) -conjugated mouse anti-human mAbs from Becton Dickinson (BD, San Jose, CA, USA), from Dako (DK, Glostrup, Denmark) and from Biocytex (Marseille, France), whose catalogue numbers (Ref.) are indicated below.

- PE-conjugated mouse anti-human CD42b (GPIb) (clone AN51; DK, Ref. R7014)
- FITC-conjugated mouse anti-human PAC-1 (clone PAC-1; BD, Ref. 340507)
- FITC-conjugated mouse anti-human bound fibrinogen (clone 9F9; Biocytex, Ref. 5009-F100T).

The anti-CD42b mAb was used for gating the PLT cell population and anti-PAC1 and anti-bFG mAbs are used to measure PLT activation.

Background staining is evaluated in both cases using FITC- and PE-conjugated isotype mAbs.

*Comments*

*Anti-CD61 and anti-CD41a mAbs are specific for the proteins of the GPIIb/IIIa complex: anti-CD61 recognizes the GPIIIa, whereas anti-human CD41a recognizes the calcium dependent GPIIb/IIIa complex. Anti-CD42b is specific for the GPIb glycoprotein. CD41a expression increases after PLT activation ([Matzdorff A, 2006](https://www.ncbi.nlm.nih.gov/pubmed/?term=15894353)), an opposite effect being observed on the levels of CD42b (*[*Michelson AD, 1994*](https://www.ncbi.nlm.nih.gov/pubmed/?term=8204882)*).*

*Anti-PAC-1 and anti-bFG mAbs are both conformation-specific, as they distinguish between resting and activated PLTs by recognizing ligand-induced binding sites (LIBS) on the GPIIb/IIIa receptor (*[*Ginsberg MH, 1990*](https://www.ncbi.nlm.nih.gov/pubmed/?term=2242424)*), and receptor induced binding sites (RIBS) on the FG ligand ([Ugarova TP, 1993](https://www.ncbi.nlm.nih.gov/pubmed/?term=7691805)), respectively. The anti-PAC-1 mAb recognizes a GPIIb/IIIa epitope expressed on activated platelets at or near the fibrinogen-binding site ([Shattil SJ, 1985](https://www.ncbi.nlm.nih.gov/pubmed/?term=2411729)) (*[*Ginsberg MH, 1990*](https://www.ncbi.nlm.nih.gov/pubmed/?term=2242424)*). Anti-human bFG is specific for human FG bound to the GPIIb/IIIa receptor by recognizing the 9F9 epitope located in the NH2-terminal part of the γ-chain of fragment D (g112-119) [Ugarova TP, 1993](https://www.ncbi.nlm.nih.gov/pubmed/?term=7691805)). Both have been used for a long time to detect activated platelets in humans ([Abrams CS, 1990](https://www.ncbi.nlm.nih.gov/pubmed/?term=2294986)).*

*Comments: Local FCM laboratories should implement IQC programs for the reagents used, especially concerning the mAbs, in order to prevent disparities related to antibody degradation (e.g. improper use after the expiration date, unappropriated storage conditions) and lot-to-lot variations.*

##### Platelet agonists

PLT agonists used in PLT activation studies included the P2Y12 agonist ADP (Chrono-Log Corporation – CLC, Havertown, PA, USA; catalogue number PN-384) (final concentration: 10 µM) and the protease activated receptor (PAR)-1 agonist thrombin receptor activator peptide 6 (TRAP-6) (Stago, Asnières-sur-Seine, France; catalogue number 86926) (final concentration: 20 µM).

*Comments*

*TRAP-6 and ADP have different potency as PLT agonists and diverse mechanisms of action. TRAP-6 is a synthetic hexapeptide fragment (SFLLRN) that acts as a strong agonist for the thrombin receptor (also known protease-activated receptor 1, PAR-1) and stimulates mobilization of free intracellular calcium, rapidly phosphorylates phosphodiesterase and induces PLTs to aggregate ([Vassalo RR, 1992](https://www.ncbi.nlm.nih.gov/pubmed/?term=1313429)). ADP is a weak PLT agonist that acts on the P2Y12, a G-coupled purinergic receptor ([Dorsam RT, 2004](https://www.ncbi.nlm.nih.gov/pubmed/?term=14755328)).*

##### Other reagents

- Bovine Serum Albumin (BSA) (Sigma, Ref. A3294-50G)
- Ethylenediaminetetraacetic acid dipotassium salt dihydrate, puriss. p.a., >=99.0% (KT) (EDTA-K2) (Sigma-Aldrich; Ref. SAF-03660-100G).
- Phosphate buffered saline (PBS) (15 packets; 15x500mL) (BC, Ref. 6603369) – Each package contains 15 foil-wrapped packets of PBS Buffer reagent. Each package is brought up to a 500 mL volume with distilled water to yield a 0.01 M potassium phosphate, 0.15 M sodium chloride at a pH of 7.2 ± 0.2.
- ADP (Chrono-Log Corporation – CLC, Havertown, PA, USA; Ref. PN-384) – Each vial contains 2.5mg of lyophilized adenosine diphosphate.
- TRAP-6 (Protease activated receptor (PAR)-1 agonist thrombin receptor activator peptide 6) (Stago, Asnières-sur-Seine, France; Ref. 86926) – Each package contains 3 vials of 1 mL; concentration: 500 µM; stability 18-25ºC: 8 hours; 2-8ºC: 2 weeks; -20ºC: 1 month.

#### Solutions

- PBS-BSA(0.2%) containing 9 mM EDTA – PBS-BSA(0.2%)-EDTA(9mM) – conserved at the refrigerator (-4ºC) and less to stay at room temperature before use.
- Formaldehyde stock solution 10% conserved at room temperature.
- TRAP-6 and ADP solutions:
  - TRAP-6 (500 μM) and ADP (1000 μM) stock solutions, conserved frozen at -20ºC:
  - Thaw aliquots of TRAP-6 and ADP stock solutions at room temperature at the day of the experiment, and prepare TRAP-6 and ADP working solutions as follows:
    - TRAP-6 working solution (80 μM): 20 μL TRAP-6 stock solution (500 μM) + 105 μL distilled H_2_O
    - ADP working solution (40 μM): 20 μL ADP stock solution (1000 μM) + 480 μL saline.

#### Technical protocol

##### Platelet activation

The citrated whole PB samples (1 patient e 2 healthy individuals processed in parallel) are incubated (2 minutes in darkness at room temperature) either with PBS-BSA containing 6.25 mM EDTA-K3 (PBS-BSA(0.2%)-EDTA(6.25mM) (inhibitory conditions), with PBS-BSA (basal conditions), or with PBS-BSA containing a physiological PLT agonist (stimulatory conditions), either ADP (10 µM) or TRAP-6 (20 µM).

##### Platelet staining

Platelet staining is performed by adding 10 µL of PE-conjugated anti-CD42b mAb, and 20 µL of FITC-conjugated anti-PAC-1 or 10 µL of FITC conjugated anti-bFG (+ 10 µL of PBS in the last case) and incubating for 10 minutes in darkness at room temperature.

*Comments*

*We recommend to stain for PAC-1 and bFG in different tubes in order to avoid steric hindrance effects because, as stated above, the epitopes recognized by these clones are in proximity, the anti-PAC-1 mAb recognizes a GPIIb/IIIa epitope expressed on activated platelets at or near the fibrinogen-binding site ([Shattil SJ, 1985](https://www.ncbi.nlm.nih.gov/pubmed/?term=2411729)) (*[*Ginsberg MH, 1990*](https://www.ncbi.nlm.nih.gov/pubmed/?term=2242424)*).*

##### Platelet fixation

After incubation, cells are suspended with 1 ml of PBS-BSA(0.2%), immediately fixed by adding 50 µL of 10% formaldehyde, mixing by vortex, and incubated for 30 minutes in the refrigerator before reading.

*Comments*

*Upon fixation, PLT markers were stable for 8 days, but in our laboratory in most cases sample acquisition is performed at the day of the experiment.*

#### Technical procedure – step by step

##### TRAP-6 experiments - PLT activation with TRAP-6 (20 µM)

| For each PB sample (patient + 2 healthy controls, prepare 3 tubes (T1-T3), as follows: | | | |
| --- | --- | --- | --- |
| T1 | 60 µL PBS-BSA(0.2%)-EDTA(9mM) | | + 20 μL PB to each tube. |
| T2 | 60 µL PBS-BSA(0.2%) | |  |
| T3 | 40 µL PBS-BSA(0.2%) + 20 µL TRAP-6 (80 µM) | |  |
| Vortex for 1 to 2 seconds. | | | |
| Incubate for 2 minutes, in darkness at room temperature (~22ºC) | | | |
| For each PB sample (patient + 2 healthy controls, prepare T4-T12, as follows. | | | |
| T4 | 10 μl CD42b to each tube | + 20 μL anti-PAC-1 to each tube | + 20 µL T1 |
| T5 |  |  | + 20 µL T2 |
| T6 |  |  | + 20 µL T3 |
| T7 |  | + 10 μL anti-bFG + 10 μL PBS to each tube | + 20 µL T1 |
| T8 |  |  | + 20 µL T2 |
| T9 |  |  | + 20 µL T3 |
| T10 |  | + 20 μL negative isotype control to each tube | + 20 µL T1 |
| T11 |  |  | + 20 µL T2 |
| T12 |  |  | + 20 µL T3 |
| Vortex for 1 to 2 seconds. | | | |
| Incubate for 10 minutes, in darkness at room temperature (~22ºC). | | | |
| Add 2 mL PBS-BSA(0.2%) to all tubes (T4-T12). | | | |
| Vortex for 1 to 2 seconds. | | | |
| Separate 1 mL from each tube to another tube with the same designation. | | | |
| Add 50 µL formaldehyde 10% (final concentration: 0.5%). | | | |
| Vortex for 1 to 2 seconds. | | | |
| Incubate 30 minutes, in darkness, at the refrigerator (~4ºC). (*) Start to prepare the tubes for ADP | | | |
| Vortex for 1 to 2 seconds. | | | |
| Acquire in the flow cytometer. | | | |

##### ADP experiments - PLT activation with ADP (10 µM)

| For each PB sample (patient + 2 healthy controls, prepare 3 tubes (A1-A3), as follows: | | | |
| --- | --- | --- | --- |
| A1 | 60 µL PBS-BSA(0.2%)-EDTA(9mM) | | + 20 μL PB to each tube |
| A2 | 60 µL PBS-BSA(0.2%) | |  |
| A3 | 40 µL PBS-BSA(0.2%) + 20 µL TRAP-6 (80 µM) | |  |
| Vortex for 1 to 2 seconds. | | | |
| Incubate for 2 minutes, in darkness at room temperature (~22ºC) | | | |
| For each PB sample (patient + 2 healthy controls, prepare T4-T12, as follows. | | | |
| A4 | 10 μl CD42b to each tube | + 20 μL anti-PAC-1 to each tube | + 20 µL A1 |
| A5 |  |  | + 20 µL A2 |
| A6 |  |  | + 20 µL A3 |
| A7 |  | + 10 μL anti-bFG + 10 μL PBS to each tube | + 20 µL A1 |
| A8 |  |  | + 20 µL A2 |
| A9 |  |  | + 20 µL A3 |
| A10 |  | + 20 μL negative isotype control to each tube | + 20 µL A1 |
| A11 |  |  | + 20 µL A2 |
| A12 |  |  | + 20 µL A3 |
| Vortex for 1 to 2 seconds. | | | |
| Incubate for 10 minutes, in darkness at room temperature (~22ºC). | | | |
| Add 2 mL PBS-BSA(0.2%) to all tubes (A4-A12). | | | |
| Vortex for 1 to 2 seconds. | | | |
| Separate 1 mL from each tube to another tube with the same designation. | | | |
| Add 50 µL formaldehyde 10% (final concentration: 0.5%). | | | |
| Vortex for 1 to 2 seconds. | | | |
| Incubate 30 minutes, in darkness, at the refrigerator (~4ºC). | | | |
| Vortex for 1 to 2 seconds. | | | |
| Acquire in the flow cytometer. | | | |

| Final concentrations |  |
| --- | --- |
| **EDTA-K3** (T1 and A1) | 6.25 mM [60 μL EDTA (9 mM) + 20 μL PB] |
| **TRAP-6** (T3) | 20 μM [40 μL PBS-BSA(0.2%) + 20 μL TRAP-6 (80 μM) + 20 μL PB] |
| **ADP** (A3) | 10 μM [40 μL PBS-BSA(0.2%) + 20 μL ADP (40 μM) + 20 μL PB] |
| **Formaldehyde** (T4-T12 and A4-A12) | 0.5% (50 μL stock solution 10% in 1mL of platelet suspension) |

#### Sample acquisition

Samples are acquired in a Navios^TM^ flow cytometer (BC). At least 5.000 PLT events are acquired and stored as listmode (lmd) files to be subsequently analyzed.

#### Data analysis

PLTs are gated based on their light scatter properties (FSC and SSC) and on the expression of CD42b (PE channel) (**Figure 2**).

Fluorescence intensity in the FITC channel is used evaluate the expression of activated GPIIb/IIIa receptor, either directly (as identified by the PAC-1 mAb), or indirectly (using the bFG mAb).

The level of PE fluorescence emitted by the PLTs allows to estimate the level of PLT activation in the sample.

The inhibitory tube (EDTA) is used as a negative control to discriminate between activated and non-activated platelets.

#### Expression of results

##### Expressing platelet activation using the Platelet Activation Index

Platelet activation is evaluated by calculating the Platelet Activation Index (PAI) expressed as fold increase in the MFI obtained for a given activation-related molecule (PAC-1 or bFG) in the test tubes (i.e., basal conditions, ADP-stimulated, and TRAP-6-stimulated), comparatively to the MFI obtained in the corresponding inhibitory conditions (EDTA tubes): (MFI test tube - MFI inhibitory tube) / MFI inhibitory tube.

For each case studied, six PAIs are calculated: two for basal conditions (BAS/PAC-1 and BAS/bFG), two for stimulation with ADP (ADP/PAC-1 and ADP/bFG), and another two for stimulation with TRAP-6 (TRAP-6/PAC-1 and TRAP-6/bFG).

##### Expressing PLT activation as % of the normal values

In addition, the expression of the activation related markers (PAC-1 or bFG) in patients´ PLTs is compared to that observed in healthy controls, by calculating the ratio between the MFI obtained in each patient and the mean MFI obtained in controls processed in the same conditions, expressed as percentage of the normal values (MFI of patient / mean MFI of controls*100).

**Figure 2.** Dot plots and histograms illustrating a typical PLT activation experiment using TRAP-6 as agonist. Platelets are selected based on their FSC and SSC, and on the expression of CD42b (FL2) (first row) and then analyzed for the expression of PAC-1 (second row) and bFG (third row) epitopes on the PLT surface (FL1) under A) inhibitory (EDTA); B) basal / unstimulated (PBS) and C) stimulatory (TRAP-6, 20 µM) conditions, respectively. FITC conjugated isotype matched mAb with irrelevant specificity are used as negative control (fourth row). Numbers inside the histograms indicate the median fluorescence intensity in FL1 channel.

#### Expected results

*Comments*

*Previous studies have demonstrated gender and age influence in PLT activation responses as evaluated by FCM, with PLT activation in response to ADP being higher in women than in men and increasing with age. In our study we did evaluate the effect of these parameters.*

*Each laboratory should validate these reference intervals using their specific experimental conditions.*

##### Normal Reference Values

A total of 26 control PB samples (healthy adults, blood donors) were studied till the moment, including the 12 PB samples studied in parallel with GTLS patients described in the present work (**Table 2**).

Two of the 26 healthy controls (8%) had evidence of PLT activation in unstimulated (basal) conditions when evaluated by PAC-1 binding, having basal PAI values of 26 and 37 in TRAP-6 experiments and of 17 and 44 in ADP experiments, respectively, comparatively of median PAI values of 2, ranging from 1 to 9 in TRAP-6 experiments and from 1 to 12 in ADP experiments, observed in the remaining 24 healthy controls.

None of the healthy controls had evidence of PLT activation in unstimulated (basal) conditions when evaluated by the expression of bFG on the PLT surface.

The cut-off values used to discriminate normal from abnormal results are presented below and are based on the 5th-95th percentile of the results for PAI and % of NV obtained in the 26 healthy controls studied, in TRAP-6 or ADP experiments, when PLT activation was evaluated using anti-PAC-1 or anti-bFG mAbs, respectively (**Table 2**).

- Decreased PLT activation in response to TRAP-6 (20 µM):
  - Evaluation with anti-PAC-1: PAI <73; %NV <73%
  - Evaluation with anti-bFG: PAI <7; %NV <42%
- Decreased PLT activation in response to ADP (10 µM):
  - Evaluation with anti-PAC-1: PAI <72; %NV <72%
  - Evaluated with anti-bFG: PAI <11; %NV <46%
- Increased PLT activation in basal conditions (no agonist):
  - Evaluation with anti-PAC-1: PAI >19 (TRAP-experiments); PAI >16 (ADP-experiments)
  - Evaluation with anti-bFG: PAI >1 (TRAP-experiments and ADP-experiments)

##### Expected Results in GTLS Patients

GTLS patients are expected to have variable decreased levels of PLT activation (evaluated by the expression of PAC-1 and bFG on the PLT surface) upon stimulation with TRAP-6 and ADP compared to that observed in controls (healthy individuals), with a variable decrease of the correspondent PAIs and lower percentages of PAC-1 and bFG, expressed as % of the normal values.

Some GTLS patients may have residual PLT activation at basal conditions, as evaluated by the expression of PAC-1 epitopes, but not when evaluated by the expression of bFG epitopes, suggesting that this residual PLT activation is abortive, i.e., it does no traduces in effective FG binding to the GPIIbIIIa complex. Further studies are needed in order to better understand the relevance of this phenomena, and if there is a relationship to the genetic variants.

**Table 2.** Platelet activation indexes at basal conditions and upon stimulation with TRAP-6 or ADP, as evaluated by the expression of PAC-1 and bFG on the PLT surface the 12 healthy controls studied in parallel with GTLS patients, and comparatively to a total of 26 healthy controls studied till now.

| **Experiments (agonist)** | **mAbs** | **Conditions** | **Statistic** | **N=12** | | **N=26** | |
| --- | --- | --- | --- | --- | --- | --- | --- |
|  |  |  |  | **PAI** | **% NV** | **PAI** | **% NV** |
| **TRAP-6** | **PAC-1** | **Unstimulated**  **(PBS)** | Median (range) | 5 (2-37) * | NA | 3 (1-37) * | NA |
|  |  |  | Mean ± SD | 9±11 | NA | 6±8 | NA |
|  |  |  | P25-P75 | 2-8 | NA | 2-6 | NA |
|  |  |  | P10-P90 | 2-21 | NA | 1-8 | NA |
|  |  |  | P5-P95 | 2-**29** | NA | 1-**19** | NA |
|  |  | **Stimulated**  **(TRAP-6 20 µM)** | Median (range) | 136 (100-194) | 100 (76-147) | 108 (69-194) | 100 (72-171) |
|  |  |  | Mean ± SD | 144±39 | 102±29 | 122±40 | 105±25 |
|  |  |  | P25-P75 | 113-172 | 88-110 | 93-157 | 90-119 |
|  |  |  | P10-P90 | 101-193 | 82-122 | 79-184 | 75-136 |
|  |  |  | P5-P95 | **100**-194 | **79**-134 | **73**-191 | **73**-141 |
|  | **bFG** | **Unstimulated**  **(PBS)** | Median (range) | 0 (0-1) ** | NA | 0 (0-1) ** | NA |
|  |  |  | Mean ± SD | 0±1 | NA | 0±0 | NA |
|  |  |  | P25-P75 | 0-0 | NA | 0-0 | NA |
|  |  |  | P10-P90 | 0-1 | NA | 0-1 | NA |
|  |  |  | P5-P95 | 0-**1** | NA | 0-**1** | NA |
|  |  | **Stimulated**  **(TRAP-6 20 µM)** | Median (range) | 13 (7-33) | 100 (40-180) | 15 (6-33) | 100 (32-180) |
|  |  |  | Mean ± SD | 15±8 | 101±49 | 16±7 | 99±41 |
|  |  |  | P25-P75 | 9-18 | 61-127 | 10-18 | 70-121 |
|  |  |  | P10-P90 | 9-24 | 50-175 | 7-25 | 51-159 |
|  |  |  | P5-P95 | **8**-28 | **45**-177 | **7**-31 | **42**-174 |
| **ADP** | **PAC-1** | **Unstimulated**  **(PBS)** | Median (range) | 6 (1-44) *** | NA | 3 (1-44) *** | NA |
|  |  |  | Mean ± SD | 9±12 | NA | 6±9 | NA |
|  |  |  | P25-P75 | 2-9 | NA | 2-6 | NA |
|  |  |  | P10-P90 | 1-16 | NA | 1-10 | NA |
|  |  |  | P5-P95 | 1-**29** | NA | 1-**16** | NA |
|  |  | **Stimulated**  **ADP (10 µM)** | Median (range) | 133 (63-205) | 100 (77-156) | 109 (63-205) | 100 (68-156) |
|  |  |  | Mean ± SD | 130±40 | 106±20 | 120±40 | 101±22 |
|  |  |  | P25-P75 | 99-155 | 95-112 | 90-140 | 89-112 |
|  |  |  | P10-P90 | 85-171 | 90-124 | 78-175 | 75-127 |
|  |  |  | P5-P95 | **75-**187 | **84**-139 | **72**-198 | **72**-144 |
|  | **bFG** | **Unstimulated**  **(PBS)** | Median (range) | 0 (0-1) **** | NA | 0 (0-1) **** | NA |
|  |  |  | Mean ± SD | 0±0 | NA | 0±0 | NA |
|  |  |  | P25-P75 | 0-0 | NA | 0-0 | NA |
|  |  |  | P10-P90 | 0-1 | NA | 0-1 | NA |
|  |  |  | P5-P95 | 0-**1** | NA | 0-**1** | NA |
|  |  | **Stimulated**  **ADP (10 µM)** | Median (range) | 17 (8-31) | 100 (44-174) | 18 (8-36) | 100 (41-167) |
|  |  |  | Mean ± SD | 19±8 | 100±38 | 20±7 | 101±33 |
|  |  |  | P25-P75 | 13-25 | 69-121 | 16-25 | 87-122 |
|  |  |  | P10-P90 | 11-28 | 59-140 | 12-28 | 58-134 |
|  |  |  | P5-P95 | **10**-29 | **52**-155 | **11**-30 | **46**-154 |

Abbreviations: ADP, adenosine diphosphate; bFG, bound fibrinogen; NA, not applicable; PAI, platelet activation index; TRAP-6, protease activated receptor (PAR)-1 agonist thrombin receptor activator peptide; %NV, % of the normal values.

The control population consisted of 26 healthy adult individuals (blood donors), 13 males and 13 females, with a median age of 51 years, ranging from 22 to 66 years

* In TRAP-experiments, 2 of the 26 healthy controls (8%) had evidence of PLT activation in unstimulated (basal) conditions when evaluated by anti-PAC-1 binding, having basal PAIs of 26 and 37, comparatively of median PAI values of 2, ranging from 1 to 9, observed in the remaining 24 healthy controls.

** In TRAP-experiments, none of the 26 healthy controls had evidence of PLT activation in unstimulated (basal) conditions when evaluated by anti-bFG binding.

*** In ADP-experiments, the same 2 healthy controls (8%) had evidence of PLT activation in unstimulated (basal) conditions when evaluated by PAC-1 binding, having basal PAIs of 17 and 44, respectively, comparatively of median PAI values of 2, ranging from 1 to 12 in ADP experiments, observed in the remaining 24 healthy controls.

**** In ADP-experiments, none of the 26 healthy controls had evidence of PLT activation in unstimulated (basal) conditions when evaluated by anti-bFG binding.

#### Protocol validation

As stated above, the FCM protocol described herein was developed locally, based on a commercially kit that is no longer in the market (PLATELET Fibrinogen, Biocytex, Marseille, France; catalogue number 7012).

##### Pre-validation tests

Before validating our FCM protocol for PLT activation studies we have made some pre-tests in order to establish the experimental conditions to be used, dedicating especial attention to the mAbs used to detect PLT activation and to the time between completion of sample processing and flow cytometer reading.

Concerning the staining conditions, we first used the volumes of anti-PAC1 FITC and anti-bFG FITC mAbs recommended by the manufacture (20 µL of mAb for ~1x10^6^/cells). We observed a high background staining with anti-bFG (data not shown), so we subsequently performed anti-bFG titration studies (20 µL, 10 µL and 5 µL) using TRAP-6 as agonist. We selected 10 µL as the volume of anti-bFG FITC that decreased non-specific PLT binding (background staining, inhibitory conditions, T7, and basal staining, non-stimulatory conditions, T8) without compromising the fluorescence intensity obtained in stimulatory conditions (TRAP-6, T9) (**Figure 3**).


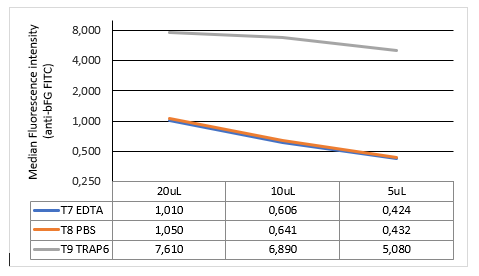


**Figure 3.** Median fluorescence intensity at FL1 of blood PLTs from a healthy control stained with 20 µL, 10 µL and 5 µL of anti-bFG FITC, at inhibitory (T7, EDTA), basal (non-stimulated; T8, PBS) and stimulatory conditions (T9, TRAP-6 10 µM).

Next, we tested the stability of the fluorescence intensity over time in 2 blood samples from healthy controls, by reading them in the flow cytometer immediately after processing (T0), 1 hour later (T1) and 3 hours later (T3). We observed that the median fluorescence intensity (MFI) rapidly decreased over time, especially for PAC-1 FITC, indicating reversibility of the activation-induced PLT changes (**Figure 4**).


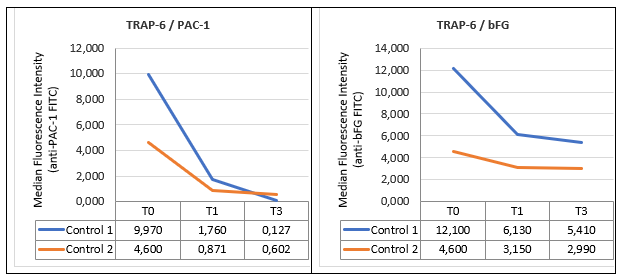


**Figure 4.** Median fluorescence intensity at FL1 of blood PLTs from 2 healthy controls stained stimulated with TRAP-6 and stained with anti-PAC1 FITC or with anti-bFG FITC, when reading in the flow cytometer immediately (T0), 1 hour after (T1) and 3 hours after (T3) finishing sample processing,

Taking in consideration what was said above, we decided to fix the PLT with 0.5% formaldehyde immediately after finishing sample processing.

We observed that PLT fixation markedly increase the MFI of staining with anti-PAC-1 FITC and anti-bFG FITC, both in TRAP-6-stimulated and in ADP-stimulated PLTs, without relevant changes in background staining (**Figure 5**), and that the MFI of the stained PLTs remained stable for at least 24 hours (**Figure 6**).


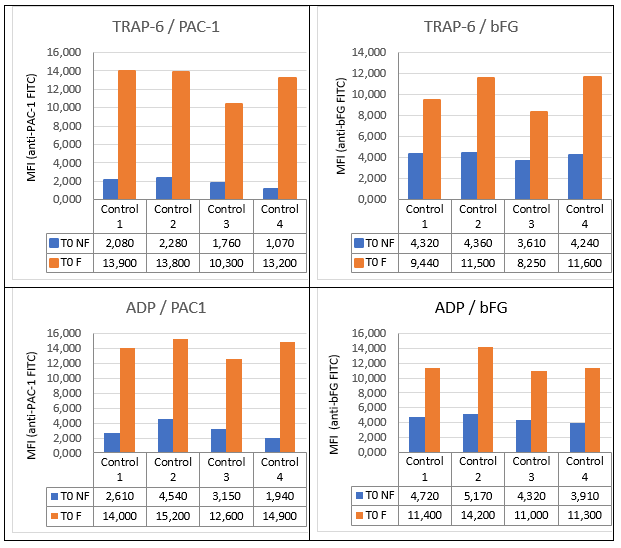


**Figure 5.** Median fluorescence intensity at FL1 of blood PLTs from 4 healthy controls stimulated with TRAP-6 or with ADP, stained with anti-PAC1 FITC or with anti-bFG FITC, and read in the flow cytometer immediately after staining (T0), without fixation (T0 NF) and after being fixed with 0.5% formaldehyde (T0 F).


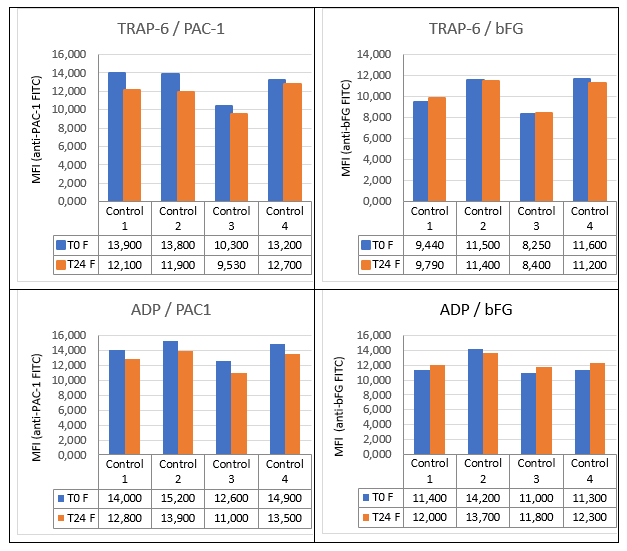


**Figure 6.** Median fluorescence intensity (MFI) at FL1 of blood PLTs from 4 healthy controls stimulated with TRAP-6 or with ADP, stained with anti-PAC1 FITC or with anti-bFG FITC, fixed with 0.5% formaldehyde and read in the flow cytometer immediately after staining (T0 F) and 24 hours after staining (T24 F).

Once established the experimental conditions, we performed validation tests, which included accuracy tests to determine the intra-operator repeatability and the reproducibility between operators (intermediate precision), as well as to estimate the sensitivity and specificity for the diagnosis of GTLS.

##### Validation tests

###### Reliability tests

Reliability tests revealed a good to excellent reproducibility between operators (intermediate precision) and intra-operator repeatability, as evaluated by the Intraclass Correlation Coefficients (ICC) for the results obtained in samples processed in parallel by to operators and in samples processed in duplicate by the same operator.

Table 2.Test reliability - Reproducibility between operators (intermediate precision) as evaluated by the Intraclass Correlation coefficient* for the results obtained in samples processed in parallel by two operators

| Measurements | T5+A5-Op1  T5+A5-Op2 | T8+A8-Op1  T8+A8-Op2 | T6-Op1  T6-Op2 | T9-Op1  T9-Op2 | A6-Op1  A6-Op2 | A9-Op1  A9-Op2 |
| --- | --- | --- | --- | --- | --- | --- |
| Experiment | TRAP-6 or  ADP | TRAP-6 or  ADP | TRAP-6 | TRAP-6 | ADP | ADP |
| Agonist | No (NST) | No (NST) | Yes (ST) | Yes (ST) | Yes (ST) | Yes (ST) |
| Monoclonal antibody | PAC-1 | bFG | PAC-1 | bFG | PAC-1 | bFG |
| Number of subjects (n) | 8 | 10 | 5 | 5 | NA | 3 |
| Number of raters (k) | 2 | 2 | 2 | 2 | NA | 2 |
| Model | The same raters for all subjects. Two-way model. | | | | | |
| Type | Consistency: systematic differences between raters are irrelevant. | | | | | |
| ICC (95%CI)  Single measures** | 0,6500  (-0.02883 to 0.9185) | 0.8693  (0.5607 to 0.9659) | 0.6627  (-3216 to 0.9586) | 0.7599  (-0.1342 to 0.9720) | NA | 0.7189  (-0.7280 to 0.9916) |
| ICC (95%CI)  Average measures*** | 0,7879  (-0,05937 to 9,9575) | 0.9310  (0.7185 to 0.9826) | 0,7971  (-0.9483 to 0.9789) | 0.8636  (-0.3101 to 0.9858) | NA | 0.8354  (-5.3786 to 0.9958) |

Abbreviations: ICC, Intraclass correlation coefficient; CI, confidence interval; NST, non-stimulated; ST, stimulated; NA, the results are not available.

The MedCalc Statistical Software version 16.4.3 (MedCalc Software, Ostend, Belgium) was used for statistics.

* The degree of consistency among measurements

** Estimates the reliability of single ratings; this ICC is an index for the reliability of the ratings for one, typical, single rater.

*** Estimates the reliability of averages of k ratings; this ICC is an index for the reliability of different raters averaged together and is always higher than the Single measures ICC.

Table 3. Test reliability - Intra-operator repeatability, as evaluated by the Intraclass Correlation Coefficient* for the results obtained in samples processed in duplicate by the same operator.

| Measurements | T5+A5-M1  T5+A5-M2 | T8+A8-M1  T8+A8-M2 | T6-M1  T6-M2 | T9-M1  T9-M2 | A6-M1  A6-M2 | A9-M1  A9-M2 |
| --- | --- | --- | --- | --- | --- | --- |
| Experiment | TRAP-6 or  ADP | TRAP-6 or  ADP | TRAP-6 | TRAP-6 | ADP | ADP |
| Agonist | No (NST) | No (NST) | Yes (ST) | Yes (ST) | Yes (ST) | Yes (ST) |
| Monoclonal antibody | PAC-1 | bFG | PAC-1 | bFG |  | bFG |
| Number of subjects (n) | 6 | 4 | 6 | 5 | 3 | 2 |
| Number of raters (k) | 2 | 2 | 2 | 2 | 2 | 2 |
| Model | The same raters for all subjects. Two-way model. | | | | | |
| Type | Consistency: systematic differences between raters are irrelevant. | | | | | |
| ICC (95%CIs)  Single measures** | 0,4615  (-0.4495 to 0.9019) | 0.8525  (0.1030 to 0.9897) | 0.6731  (-0,7680 to 0.9900) | 0.6270  (-0.7989 to 0.9883) | 0.6960  (-0.7497 to 0.9909) | 1.0000  (0.9933 to 1.0000) |
| ICC (95%CI)  Average measures*** | 0,6315  (1.6332 to 0.9484) | 0.9204  (0.2297 to 0.9948) | 0.8046  (6.618 to 0.9950) | 0.7707  (-7.9420 to 0.9941) | 0.8208  (-5.9897 to 0.9954) | 1.0000 (0.9966 to 1.0000) |

Abbreviations: ICC, Intraclass correlation coefficient; CI, confidence interval;

The MedCalc Statistical Software version 16.4.3 (MedCalc Software, Ostend, Belgium) was used for statistics.

* The degree of consistency among measurements

** Estimates the reliability of single ratings; this ICC is an index for the reliability of the ratings for one, typical, single rater.

*** Estimates the reliability of averages of k ratings; this ICC is an index for the reliability of different raters averaged together and is always higher than the Single measures ICC.

*Comments:*

*Cicchetti ([1994](https://pdfs.semanticscholar.org/50d7/f68422d0c0424674f6b235ac23be8300da38.pdf)) gave the following often quoted guidelines for interpretation for ICC inter-rater agreement measures: bellow 0.40 - poor; between 0.40 and 0.59 - fair; between 0.60 and 0.74 - good; between 0.75 and 1.00 – excellent reliability. Different guidelines were subsequently given by* Rosner (2006) (ICC < 0.4: poor reliability, 0.4 ≤ ICC < 0.75: fair to good reliability, and ICC ≥ 0.75: excellent reliability) and by *Koo and Li ([2016](https://www.ncbi.nlm.nih.gov/pubmed/?term=273305)) (*ICC < *0.50: poor; between 0.50 and 0.75: moderate; between 0.75 and 0.90: good; above 0.90: excellent).*

###### Specificity and sensitivity

Validation of FCM-based diagnostic tests to evaluate PLT function is difficult because of the absence of a gold standard test and because of the rarity of inherited PLT disorders.

The protocol described above was initially validated by studying 11 healthy control PB samples, 1 PB sample from a patient with GTLS with genetic diagnosis and 1 PB sample from a patient with type I GT.

After that, 9 patients with GTLS with genetic diagnosis (including the patient mentioned above) from 8 different GTLS families were studied together with another 12 PB samples from healthy controls.

Subsequently, 5 patients with other types of familial thrombocytopenia with normal GPIIb/IIIa levels due to other genetic defects were evaluated, together with another 6 normal PB samples. In addition, the study of one of the GTLS patients who had been previously studied was repeated in a different time with similar results.

In overall, the study population consisted of 26 healthy adult individuals, 13 males (50%), with a median age of 51 years, ranging from 22 to 66 years, and 14 patients, 6 males and 9 females, with a median age of 45 years, ranging from 12 to 66 years (**Table 4**). The patient´s group included 9 patients with GTLS (7 females, 2 males; median range of 45 years, ranging from 19 to 66 years) and 6 patients with other inherited PLT disorders (2 females, 4 males, median age of 31 years, ranging from 12 to 53 years), from which 1 patient had GT.

The median PLT counts were of 222x10^9^/L in healthy controls, ranging from 117 to 299x10^9^/L, and of 118x10^9^/L in patients, ranging from 69 to 328x10^9^/L, respectively. Patients with GTLS had a median PLT count of 83x10^9^/L (range: 69 - 130x10^9^/L), whereas the in the remaining patients studied the median PLT count was of 264x10^9^/L (range: 183 - 328x10^9^/L).

Table 4. Blood samples used to evaluate FCM test sensibility and specificity.

|  | Blood samples | | | |
| --- | --- | --- | --- | --- |
| Patients | Initial validation | This study | Subsequent studies | Total |
| Healthy controls | 11 | 12 | 6 | 26 |
| GT patients | 1 | 0 | 0 | 1 |
| GTLS patients | 1 | 9* | 1** | 11*** |
| Patients with other IPD | 0 | 0 | 5 | 5 |
| Total | 13 | 21* | 12** | 43*** |

Abbreviations: GT, Glanzmann´s Thrombasthenia; GTLS, Glanzmann´s Thrombasthenia-like syndrome; ITP, Inherited Platelet Disorders.

* One GTLS patient was evaluated in the initial validation procedures and in this study; **One of the GTLS patients included in this study was subsequently re-evaluated; *** Two GTLS patients were studied in different occasions with similar results.

Considering that the gold standard for diagnosis of GTLS consist on the demonstration of macrothrombocytopenia associated with partial deficiency of GPIIb/IIIa, usually with a positive family history suggesting an AD pattern of inheritance, and having a documented variants in the *ITGA2B* or *ITGB3* genes), the approach used in this protocol (familial macrothrombocytopenia + partial deficiency of GPIIb/IIIa + decreased PLT activation in response to TRAP-6 and ADP as evaluated by the expression of PAC-1 and bFG epitopes on the PLT surface) revealed a sensitivity and a specificity of 100% for the diagnosis of GTLS.

### REFERENCES

**Abrams CS**, Ellison N, Budzynski AZ, Shattil SJ. Direct detection of activated platelets and platelet-derived microparticles in humans. Blood. 1990;75(1):128-38. PMID: 2294986

**Bury L**, Falcinelli E, Chiasserini D, Springer TA, Italiano JE Jr, Gresele P. Cytoskeletal perturbation leads to platelet dysfunction and thrombocytopenia in variant forms of Glanzmann thrombasthenia. Haematologica. 2016 Jan;101(1):46-56. DOI: 10.3324/haematol.2015.130849. PMID: 26452979

**Cicchetti DV**, Domenic V. Guidelines, criteria, and rules of thumb for evaluating normed and standardized assessment instruments in psychology. Psychological Assessment. 1994; 6 (4): 284–290. DOI:10.1037/1040-3590.6.4.284.

**Dorsam RT**, Kunapuli SP. Central role of the P2Y12 receptor in platelet activation. J Clin Invest. 2004;113: 340-345. DOI: 10.1172/JCI20986. PMID: 14755328. Review

**Favier M**, Bordet JC, Favier R, Gkalea V, Pillois X, Rameau P, Debili N, Alessi MC, Nurden P, Raslova H, Nurden A. Mutations of the integrin αIIb/β3 intracytoplasmic salt bridge cause macrothrombocytopenia and enlarged platelet α-granules. Am J Hematol. 2018 Feb;93(2):195-204. DOI: 10.1002/ajh.24958.PMID: 29090484

**Ginsberg MH**, Frelinger AL, Lam SC, Forsyth J, McMillan R, Plow EF, Shattil SJ. Analysis of platelet aggregation disorders based on flow cytometric analysis of membrane glycoprotein IIb-IIIa with conformation-specific monoclonal antibodies. Blood. 1990;76(10):2017-23. PMID: 2242424

**Glier H**, Novakova M, Te Marvelde J, Bijkerk A, Morf D, Thurner D, Rejlova K, Lange S, Finke J, van der Sluijs-Gelling A, Sedek L, Flores-Montero J, Böttcher S, Fernandez P, Ritgen M, van Dongen JJM, Orfao A, van der Velden VHJ, Kalina T. Comments on EuroFlow standard operating procedures for instrument setup and compensation for BD FACS Canto II, Navios and BD FACS Lyric instruments. J Immunol Methods. 2019 Dec;475:112680. DOI: 10.1016/j.jim.2019.112680. PMID: 31655051

**Huskens D**, Sang Y, Konings J, van der Vorm L, de Laat B, Kelchtermans H, et al. Standardization and reference ranges for whole blood platelet function measurements using a flow cytometric platelet activation test. PloS One. 2018;13: e0192079. DOI: 10.1371/journal.pone.0192079. PMID: 29389990

**Kalina T**, Flores-Montero J, Lecrevisse Q, Pedreira CE, van der Velden VHJ, Novakova M, Mejstrikova E, Hrusak O, Böttcher S, Karsch D, et al. Quality assessment program for EuroFlow protocols: summary results of four-year (2010-2013) quality assurance rounds. Cytometry A. 2015;87:145-56. DOI: 10.1002/cyto.a.22581. PMID: 25345353

**Kalina T**, Flores-Montero J, van der Velden VHJ, Martin-Ayuso M, Böttcher S, Ritgen M, Almeida J, Lhermitte L, Asnafi V, Mendonça A, et al. EuroFlow standardization of flow cytometer instrument settings and immunophenotyping protocols. Leukemia. 2012;26:1986-2010. DOI: 10.1038/leu.2012.122. PMID: 22948490

**Koo TK**, Li MY. A guideline of selecting and reporting intraclass correlation coefficients for reliability research. J Chiropr Med. 2016;15(2):155-63. DOI: 10.1016/j.jcm.2016.02.012. PMID 27330520.

**Kunishima S**, Kashiwagi H, Otsu M, Takayama N, Eto K, Onodera M, Miyajima Y, Takamatsu Y, Suzumiya J, Matsubara K, Tomiyama Y, Saito H. Heterozygous ITGA2B R995W mutation inducing constitutive activation of the αIIbβ3 receptor affects proplatelet formation and causes congenital macrothrombocytopenia. Blood. 2011 May 19;117(20):5479-84. DOI: 10.1182/blood-2010-12-323691. PMID: 21454453

**Matzdorff A**, Voss R. Upregulation of GP IIb/IIIa receptors during platelet activation: influence on efficacy of receptor blockade. Thromb Res. 2006;117(3):307-14. DOI: 10.1016/j.thromres.2005.03.007. PMID: 15894353.

**Michelson AD**, Benoit SE, Kroll MH, Li JM, Rohrer MJ, Kestin AS, Barnard MR. The activation-induced decrease in the platelet surface expression of the glycoprotein Ib-IX complex is reversible. Blood. 1994 Jun 15;83(12):3562-73. PMID: 8204882

**Michelson AD**. Flow cytometry: a clinical test of platelet function—a review. Blood 1996; 87:4925–4936. PMID: 8652804.

**Miyashita N**, Onozawa M, Hayasaka K, Yamada T, Migita O, Hata K, Okada K, Goto H, Nakagawa M, Hashimoto D, Kahata K, Kondo T, Kunishima S, Teshima T. A novel heterozygous ITGB3 p.T720del inducing spontaneous activation of integrin αIIbβ3 in autosomal dominant macrothrombocytopenia with aggregation dysfunction. Ann Hematol. 2018 Apr;97(4):629-640. DOI: 10.1007/s00277-017-3214-4. PMID: 29380037

**Mody M**, Lazarus AH, Semple JW, Freedman J. Preanalytical requirements for flow cytometric evaluation of platelet activation: choice of anticoagulant. Transfus Med. 1999 Jun;9(2):147-54. DOI: 10.1046/j.1365-3148.1999.00188.x. PMID: 10354385

**Nurden AT**, Pillois X, Fiore M, Heilig R, Nurden P. Glanzmann thrombasthenia-like syndromes associated with Macrothrombocytopenias and mutations in the genes encoding the αIIbβ3 integrin. Semin Thromb Hemost. 2011;37: 698-706. DOI: 10.1055/s-0031-1291380. PMID: 22102273. Review.

**Nurden AT**, Pillois X, Wilcox DA. Glanzmann thrombasthenia: state of the art and future directions. Semin Thromb Hemost. 2013 Sep;39(6):642-55. DOI: 10.1055/s-0033-1353393. PMID: 23929305. Review.

**Nurden AT**, Pillois X. ITGA2B and ITGB3 gene mutations associated with Glanzmann thrombasthenia. Platelets. 2018 Jan;29(1):98-101. DOI: 10.1080/09537104.2017.1371291. PMID: 29125375. Review.

**Pedersen OH**, Nissen PH, Hvas A-M. Platelet function investigation by flow cytometry: Sample volume, needle size, and reference intervals. Platelets. 2018;29: 199-202. DOI: 10.1080/09537104.2017.1353684. PMID: 28960147

**Rosner B**. Fundamentals of Biostatistics. 6th ed. Duxbury: Thomson Brooks/Cole; 2006.

**Shattil SJ**, Hoxie JA, Cunningham M, Brass LF. Changes in the platelet membrane glycoprotein IIb.IIIa complex during platelet activation. J Biol Chem. 1985;260: 11107-11114. PMID: 2411729

**Ugarova TP**, Budzynski AZ, Shattil SJ, Ruggeri ZM, Ginsberg MH, Plow EF. Conformational changes in fibrinogen elicited by its interaction with platelet membrane glycoprotein GPIIb-IIIa. J Biol Chem. 1993;268(28):21080-7. PMID: 7691805

**van Asten I**, Schutgens REG, Baaij M, Zandstra J, Roest M, Pasterkamp G, et al. Validation of flow cytometric analysis of platelet function in patients with a suspected platelet function defect. J Thromb Haemost. 2018;16: 689-698. DOI: 10.1111/jth.13952. PMID: 29337406

**van Asten I**, Schutgens REG, Urbanus RT. Toward Flow Cytometry Based Platelet Function Diagnostics. Semin Thromb Hemost. 2018;44: 197-205. DOI: 10.1055/s-0038-1636901. PMID: 29554715. Review.

**Vassallo RR**, Kieber-Emmons T, Cichowski K, Brass LF. Structure-function relationships in the activation of platelet thrombin receptors by receptor-derived peptides. J Biol Chem. 1992;267: 6081-6085. PMID: 1313429

### ABBREVIATIONS

ADP, adenosine diphosphate

AIBS, activation-induced binding sites

BAS, basal conditions (before stimulation)

BC, Beckman Coulter, Hialeah, FL, USA

BD, Becton Dickinson, San Jose, CA, USA

bFG, bound fibrinogen

BSA, bovine serum albumin

CLC, Chrono-Log Corporation, Havertown, PA, USA

DK, Dako, Glostrup, Denmark

EDTA, ethylene diamine tetracetic acid

FCM, flow cytometry

FG, fibrinogen

FITC, fluorescein isothiocyanate

FSC, forward scatter

GP, glycoprotein

GTLS, Glanzmann´s Thrombasthenia like syndrome

IOT, Immunotech

LIBS, ligand-induced binding sites (on the GPIIb/IIIa receptor)

mAb, monoclonal antibody

MFI, median fluorescence intensity

NV, normal values

PAI, platelet activation index

PAI/ADP/bFG, PAI upon stimulation with ADP, as evaluated with anti-bFG monoclonal antibody

PAI/ADP/PAC-1, PAI upon stimulation with ADP, as evaluated with anti-PAC-1 monoclonal antibody

PAI/BAS/bFG, PAI at basal conditions, as evaluated with anti-bFG monoclonal antibody

PAI/BAS/PAC-1, PAI at basal conditions, as evaluated with anti-PAC-1 monoclonal antibody

PAI/TRAP-6/bFG, PAI upon stimulation with TRAP-6, as evaluated with anti-bFG monoclonal antibody

PAI/TRAP-6/PAC-1, PAI upon stimulation with TRAP-6, as evaluated with anti-PAC-1 monoclonal antibody

PB, peripheral blood

PBS, phosphate buffered saline

PBS-BSA(0.2%), PBS containing 0.2% (w/v) of bovine serum albumin

PBS-BSA(0.2%)-EDTA(9mM), PBS containing 0.2% (w/v) of bovine serum albumin and EDTA 9 mM.

PE, phycoerythrin

PLT, platelet

RIBS, receptor induced binding sites (on the GPIIb/IIIa receptor ligand, fibrinogen)

SSC, sideward scatter

TRAP-6, protease activated receptor (PAR)-1 agonist thrombin receptor activator peptide

### TECHNICAL SHEETS (MABS)

- PE-conjugated mouse anti-human CD42b (GPIb) (clone AN51, IgG2a/k; DK, Ref. R7014)
- FITC-conjugated mouse anti-human CD41a (GPIIbIIIa) (clone HIP8, IgG1/k; BD, Ref. 333147)
- FITC-conjugated mouse anti-human CD61 (GPIIIa) (clone RUU-PL7F12, IgG1/k; BD, Ref. 347407)
- PE-conjugated mouse anti-human CD62P (P-Selectin) (clone CLBThromb/6; IgG1; BC Immunotech, Ref. IM1759)
- FITC-conjugated mouse anti-human PAC-1 (clone PAC-1; IgM/k; BD, Ref. 340507)
- FITC-conjugated mouse anti-human bound fibrinogen (clone 9F9, IgG1/k; Biocytex, Ref. 5009-F100T).
- Platelet Fibrinogen KIT (bFG) (Biocytex, Marseille, France; Ref. 7012)
- Platelet Gp Screen KIT (CD61/GPIIIa, CD42b/GPIb, CD49b/GPIa) (Biocytex, Marseille, France; Ref. 7008)
- Platelet Gp/Receptors KIT (CD41/GPIIb, CD42b/GPIb, CD62P/GMP-140) (Biocytex (Marseille, France; Ref. 7004).
