# Supplementary material for: αIIbβ3 variants in ten families with autosomal dominant macrothrombocytopenia: Expanding the mutational and clinical spectrum
Source: PLoS One. 2020 Dec 4;15(12):e0235136. doi: 10.1371/journal.pone.0235136 (PMC7717987; doi:10.1371/journal.pone.0235136)
Supplement: S2 File — (DOCX) [file pone.0235136.s002.docx]

Title**: αIIbβ3 variants in ten families with autosomal dominant macrothrombocytopenia: expanding the mutational and clinical spectrum**

Short title: **Familial macrothrombocytopenia with αIIbβ3 integrin deficiency**

**AUTHORS:** Sara Morais, Jorge Oliveira, Catarina Lau, Mónica Pereira, Marta Gonçalves, Catarina Monteiro, Ana Rita Gonçalves, Rui Matos, Marco Sampaio, Eugénia Cruz, Inês Freitas, Rosário Santos, Margarida Lima

**SUPPLEMENTARY FILE 2 (S2 FILE)**

## S2 File | METHODS: NEXT GENERATION SEQUENCING PANEL AND PRIMERS USED FOR GENE SEQUENCING

We describe the genes included in the Ion AmpliSeq Hematology Research Panel (Thermo Fisher Scientific, Waltham, Massachusetts, U.S.A) used for Next Generation Sequencing (NGS) (**S2 File** | **Table 1**) and the primers used for Sanger sequencing, to confirm the presence of the pathogenic variants detected by NGS (**S2 File** | **Table 2**).

### S2 File | Table 1. List of the 394 genes included in the Ion AmpliSeq Hematology Research Panel (Thermo Fisher Scientific) used in this study

| *ABCA1* | *ABCB6* | *ABCB7* | *ABCD1* | *ABCD4* | *ACP2* | *ACSBG1* | *ACSL6* | *ACTN1* | *ACVR1* | *ADA* | *ADAMTS10* | *ADAMTS13* | *AHSP* |
| --- | --- | --- | --- | --- | --- | --- | --- | --- | --- | --- | --- | --- | --- |
| *AK1* | *AK2* | *ALAS1* | *ALAS2* | *ALDH2* | *ALDOA* | *AMN* | *ANK1* | *ANKRD26* | *ANO6* | *ANXA4* | *ANXA5* | *AP3B1* | *APITD1* |
| *ARTN* | *ASXL1* | *ATM* | *ATR* | *ATRX* | *AVPR2* | *B3GALT4* | *B4GALT1* | *BCL11A* | *BGLAP* | *BLM* | *BLOC1S3* | *BMP4* | *BMX* |
| *BPGM* | *BRCA2* | *BRIP1* | *BST1* | *BST2* | *BTF3P11* | *BTK* | *BTNL2* | *C19orf40* | *C19orf70* | *C1orf86* | *C21orf33* | *C3* | *CASP3* |
| *CBFA2T3* | *CBS* | *CCL14* | *CCND1* | *CD27* | *CD36* | *CD3D* | *CD3E* | *CD46* | *CD59* | *CD63* | *CDAN1* | *CDKN1A* | *CFB* |
| *CFH* | *CFHR5* | *CFI* | *CISD2* | *CLCN7* | *CNTN1* | *COL1A1* | *COQ2* | *COX10* | *COX4I2* | *CSF1* | *CSF2* | *CSF2RB* | *CSF3* |
| *CSF3R* | *CUBN* | *CXCL12* | *CXCR4* | *CYCS* | *DCLRE1B* | *DDX11* | *DGKE* | *DHFR* | *DIDO1* | *DKC1* | *DTNBP1* | *EGLN1* | *EIF2AK2* |
| *ELANE* | *EPAS1* | *EPB41* | *EPB42* | *ERCC1* | *ERCC2* | *F10* | *F11* | *F12* | *F13A1* | *F13B* | *F2* | *F2R* | *F2RL1* |
| *F2RL2* | *F3* | *F5* | *F7* | *F8* | *F8A3* | *F9* | *FANCA* | *FANCB* | *FANCC* | *FANCD2* | *FANCE* | *FANCF* | *FANCG* |
| *FANCI* | *FANCL* | *FANCM* | *FARS2* | *FAS* | *FASLG* | *FASN* | *FCGR2C* | *FECH* | *FERMT3* | *FGA* | *FGB* | *FGG* | *FLT1* |
| *FN1* | *FTH1* | *FTL* | *FTMT* | *FZD4* | *G6PD* | *GATA1* | *GBA* | *GCLC* | *GCLM* | *GFI1B* | *GGCX* | *GIF* | *GLA* |
| *GLRX5* | *GNAS* | *GNPDA1* | *GP1BA* | *GP1BB* | *GP5* | *GP6* | *GP9* | *GPI* | *GPX1* | *GSS* | *GSTM1* | *GSTP1* | *GSTT1* |
| *HAX1* | *HBA1* | *HBA2* | *HBB* | *HBD* | *HBG1* | *HBG2* | *HBM* | *HBQ1* | *HBZ* | *HES1* | *HK1* | *HMBS* | *HMOX1* |
| *HOOK1* | *HOXA11* | *HPS1* | *HPS3* | *HPS4* | *HPS5* | *HPS6* | *HRG* | *IDH1* | *IFNG* | *IL2RG* | *IL6* | *IL7R* | *IRF1* |
| *IRF4* | *IRF8* | *ITGA2* | *ITGA2B* | *ITGB3* | *ITK* | *JAK2* | *KCNA4* | *KITLG* | *KLF1* | *KLKB1* | *KNG1* | *KRAS* | *LCAT* |
| *LIG4* | *LIPA* | *LMAN1* | *LMBRD1* | *LRP5* | *LYST* | *MAN2B1* | *MASTL* | *MCFD2* | *MECOM* | *MIF* | *MMP9* | *MMRN1* | *MPL* |
| *MRPL36* | *MT3* | *MTAP* | *MTHFR* | *MUC5B* | *MYH9* | *NBN* | *NDP* | *NDUFS1* | *NEU1* | *NF1* | *NFKB1* | *NFKBIA* | *NHP2* |
| *NOG* | *NOP10* | *NOS3* | *NPC1* | *NPC2* | *NQO1* | *NT5C3A* | *OAT* | *P2RX1* | *P2RY12* | *PALB2* | *PARP1* | *PDCD1* | *PDGFRB* |
| *PEPD* | *PF4* | *PGAP1* | *PGK1* | *PIF1* | *PIGA* | *PIGM* | *PINX1* | *PKLR* | *PLA2G7* | *PLAT* | *PLAU* | *PLAUR* | *PLG* |
| *PNP* | *POLG* | *POT1* | *PPARG* | *PPY* | *PRF1* | *PRNP* | *PROC* | *PROCR* | *PROS1* | *PROZ* | *PSTPIP1* | *PTEN* | *PTPRC* |
| *PUS1* | *RAB27A* | *RAB40AL* | *RABL2A* | *RAC2* | *RAD50* | *RAD51* | *RAD51C* | *RAD54L* | *RAF1* | *RASGRP2* | *RBM8A* | *RHAG* | *RHCE* |
| *RMRP* | *RPL11* | *RPL26* | *RPL35A* | *RPL5* | *RPS10* | *RPS14* | *RPS17* | *RPS19* | *RPS24* | *RPS26* | *RPS7* | *RTEL1* | *RUNX1* |
| *SARS2* | *SBDS* | *SCGB1D1* | *SEC23B* | *SERPINA1* | *SERPINC1* | *SERPIND1* | *SERPINE1* | *SERPINF2* | *SERPING1* | *SF3B1* | *SF3B2* | *SFTPA1* | *SFTPA2* |
| *SH2D1A* | *SLC11A2* | *SLC19A2* | *SLC22A3* | *SLC25A19* | *SLC25A33* | *SLC25A37* | *SLC25A38* | *SLC2A1* | *SLC35A1* | *SLC40A1* | *SLC46A1* | *SLC4A1* | *SLX4* |
| *SMAD7* | *SMARCAL1* | *SMPD1* | *SPTA1* | *SPTB* | *SRP72* | *STAT1* | *STIM1* | *STK4* | *STX11* | *STXBP2* | *SUMF1* | *SYK* | *TBXA2R* |
| *TBXAS1* | *TCIRG1* | *TCN2* | *TELO2* | *TEP1* | *TERC* | *TERF1* | *TERF2* | *TERF2IP* | *TERT* | *TET2* | *TFPI* | *TFR2* | *THBD* |
| *THBS1* | *THPO* | *TINF2* | *TMPRSS6* | *TNF* | *TNFRSF11B* | *TNKS* | *TNKS1BP1* | *TNKS2* | *TPI1* | *TPMT* | *TSPAN12* | *TTC37* | *TUBB1* |
| *UNC13D* | *UROS* | *USP1* | *VENTX* | *VKORC1* | *VPS13B* | *VWF* | *WDR19* | *WIPF1* | *WRAP53* | *XK* | *XPA* | *XRCC1* | *YARS2* |
| *ZNF160* | *ZNF224* |  |  |  |  |  |  |  |  |  |  |  |  |

### S2 File | Table 2. Primers used for Sanger sequencing, to confirm the presence of the pathogenic variants detected by massive parallel sequencing

| **Gene** | **Variant** | **Region of the gene studied** | **Primer Sequence (5´-3´)** |
| --- | --- | --- | --- |
| ***ITGA2B*** | c.3020G>T | Exon 29 | F – CAGTGCCCAGCCAGAAAAG  R – TCCCTGGATTACCCACTTGG |
|  | c.3076C>T | Exon 30 | F – TCCAGGGAGGTGCTCATAGC  R – CAGCATCAGGGCTCAGTCTC |
|  | c.3077G>A |  |  |
| ***ITGB3*** | c.2236A>C | Exon 14 | F – GCCAGTTCAAGTGACTCCTGC  R – GAGACCATCTCTCCCAGTTTCC |
|  | c.2243A>C |  |  |
|  | c.2245G>C |  |  |
|  | c.2278C>T |  |  |

Abbreviations: F, forward; R, reverse
