# Supplementary material for: αIIbβ3 variants in ten families with autosomal dominant macrothrombocytopenia: Expanding the mutational and clinical spectrum
Source: PLoS One. 2020 Dec 4;15(12):e0235136. doi: 10.1371/journal.pone.0235136 (PMC7717987; doi:10.1371/journal.pone.0235136)
Supplement: S3 File — (DOCX) [file pone.0235136.s003.docx]

Title**: αIIbβ3 variants in ten families with autosomal dominant macrothrombocytopenia: expanding the mutational and clinical spectrum**

Short title: **Familial macrothrombocytopenia with αIIbβ3 integrin deficiency**

**AUTHORS:** Sara Morais, Jorge Oliveira, Catarina Lau, Mónica Pereira, Marta Gonçalves, Catarina Monteiro, Ana Rita Gonçalves, Rui Matos, Marco Sampaio, Eugénia Cruz, Inês Freitas, Rosário Santos, Margarida Lima

**SUPPLEMENTARY FILE 3 (S3 FILE)**

## S3 File | RESULTS: CLINICAL AND LABORATORY FINDINGS

### S3 File | Table 1. Individual clinical and laboratory data from the study population, including gender, year of diagnosis, age at the diagnosis, bleeding scores, platelet counts, platelet indexes, platelet morphology, and platelet glycoprotein levels evaluated by flow cytometry.

| Identifier | Gender | Disease status | BS  (ISTH-BAT)  (i) | PLT count  (x 10^9^/L) (ii) | MPV  (fL)  (ii) | PDW  (%)  (ii) | IPF  (%)  (ii) | PLT morphology  (light microscopy) | CD41a  (%) (iii) | CD61  (%) (iii) | CD42b  (%) (iii) |
| --- | --- | --- | --- | --- | --- | --- | --- | --- | --- | --- | --- |
| **Family 1** |  |  |  |  |  |  |  |  |  |  |  |
| F1.I.2 | F | P (ND) | NA | 50 | NA | NA | NA | NA | 45 | 57 | NA |
| F1.II.2 | F | P (ND) | 5 | 68 | 13.0 | 15.3 | 4.5 | Aniso; Macro; Rare giant | 32 | 35 | 131 |
| F1.III.2 | F | P (+/-) | NA | 47 | 13.5 | 20.5 | 11.8 | Aniso; Macro; Rare giant | 43 | 51 | 158 |
| F1.III.4 | F | PI (+/-) | 8 | 80 | 13.1 | 20.5 | 10.9 | Aniso; Macro; Rare giant | 45 | 56 | 155 |
| F1.III.5 | F | P (ND) | 8 | 48 | 12.6 | 13.8 | 6.5 | Aniso; Macro; Rare giant | 40 | 50 | 124 |
| F1.III.7 | F | **P (+/-)** | 7 | 87 | 13.4 | 19.2 | 10.6 | Aniso; Macro; Rare giant | 46 | 56 | 141 |
| F1.IV.3 | M | P (ND) | NA | 85 | 10.7 | 17.7 | NA | NA | 41 | 50 | 113 |
| F1.IV.4 | F | **P (+/-)** | 7 | 74 | 11.9 | 16.9 | 17.4 | Aniso; Macro; Some large | 48 | 51 | 222 |
| F1.IV.1 | F | HR (ND) | NA | 167 | 8.4 | NA | NA | NA | 80 | 96 | 106 |
| F1.IV.2 | M | HR (ND) | NA | 206 | 6.6 | NA | NA | NA | 73 | 88 | 118 |
| F1.IV.5 | F | HR (ND) | NA | 268 | NA | NA | NA | NA | 75 | 75 | 96 |
| F1.IV.6 | F | HR (ND) | NA | 425 | 6.9 | NA | NA | NA | 91 | 87 | 62 |
| **Family 2** |  |  |  |  |  |  |  |  |  |  |  |
| F2.I.2 | F | P (+/-) | 14 | 108 | 13.1 | 20.7 | 12.7 | Aniso; Macro; Rare giant | 52 | 44 | 102 |
| F2.II.1 | M | PI (+/-) | 3 | 90 | 13.0 | NA | NA | Aniso; Macro; Rare giant | 45 | 46 | 111 |
| **Family 3** |  |  |  |  |  |  |  |  |  |  |  |
| F3.II.1 | M | PI (+/-) | 3 | 77 | 18.7 | 34.6 | 34.6 | Aniso; macro | 37 | 40 | 82 |
| F3.III.2 | M | P (+/-) | 3 | 63 | 14.1 | 20.8 | 16.1 | Aniso; Macro; Rare giant | 45 | 45 | 92 |
| F3.III.1 | F | HR (-/-) | 3 | 163 | 13.7 | 21.0 | 13.5 | Aniso; Macro; Rare giant; Small aggr | 105 | 108 | 86 |
| **Family 4** |  |  |  |  |  |  |  |  |  |  |  |
| F4.II.1 | M | PI (+/-) | 9 | 97 | 14.5 | 24.1 | 21.0 | Aniso; Macro | 49 | 44 | 105 |
| **Family 5** |  |  |  |  |  |  |  |  |  |  |  |
| F5.II.1 | M | P (+/-) | 3 | 98 | 12.7 | NA | 10.9 | Aniso; Macro; Rare giant | 49 | 44 | 104 |
| F5.III.1 | F | PI (+/-) | 6 | 87 | 13.6 | 23.8 | 20.4 | Aniso; Macro; Rare giant | 48 | 50 | 104 |
| **Family 6** |  |  |  |  |  |  |  |  |  |  |  |
| F6.I.1 | M | **P (+/-)** | 7 | 74 | 14.2 | NA | 14.0 | Aniso; Macro; Rare giant; Small aggr | 45 | 49 | 88 |
| F6.II.4 | F | PI (+/-) | 6 | 71 | 12.7 | 16.4 | 10.3 | Aniso; Macro | 38 | 41 | 85 |
| F6.III.1 | F | P (+/-) | 7 | 62 | 11.9 | 19.7 | 16.2 | Aniso; Macro; Rare giant; Small aggr | 40 | 42 | 82 |
| F6.III.2 | F | **P (+/-)** | 7 | 69 | 14.9 | 22.1 | 20.2 | Aniso; Macro; Rare giant | 39 | 40 | 87 |
| F6.IV.1 | M | HR (-/-) | NA | 165 | 9.8 | 11.0 | NA | NA | 101 | 110 | 102 |
| F6.IV.2 | F | HR (ND) | NA | 263 | NA | 16.1 | NA | NA | 116 | 105 | 104 |
| **Family 7** |  |  |  |  |  |  |  |  |  |  |  |
| F7.II.2 | F | PI (+/-) | 4 | 95 | 18.7 | NA | 36.2 | Aniso; Macro; Rare giant | 65 | 58 | 116 |
| F7.II.4 | F | P (+/-) | 5 | 144 | 14.1 | 20.3 | 18.7 | Aniso; Macro; Rare giant | 62 | 62 | 102 |
| F7.III.1 | M | HR (-/-) | 0 | 294 | 11.7 | 14.3 | 5.2 | Some macro | 89 | 84 | 85 |
| **Family 8** |  |  |  |  |  |  |  |  |  |  |  |
| F8.I.1 | F | PI (ND) | NA | 48 | 10.0 | 16.1 | NA | NA | 52 | 77 | 129 |
| F8.II.2 | F | P (+/-) | 2 | 120 | 12.4 | 17.1 | 5.2 | Aniso, Macro; Rare giant; Small aggr | 77 | 80 | 104 |
| F8.III.2 | F | P (+/-) | 0 | 191 | 12.4 | 16.1 | 7.0 | Some giant | 83 | 84 | 107 |
| F8.IV.1 | F | P (+/-) | 0 | 194 | 10.2 | 12.5 | 4.1 | Normal | 80 | 83 | 112 |
| F8.III.3 | F | HR (-/-) | 0 | 208 | 11.3 | 13.8 | NA | Normal | 96 | 96 | 94 |
| **Family 9** |  |  |  |  |  |  |  |  |  |  |  |
| F9.II.2 | F | PI (+/-) | 3 | 95 | 13.2 | 17.5 | 9.4 | Aniso; Macro; Rare giant; Small aggr | 73 | 74 | 111 |
| F9.II.4 | F | P (+/-) | 3 | 102 | 12.6 | 17.2 | 7.4 | Aniso; Macro; Rare giant | 76 | 73 | 93 |
| F9.III.1 | F | P (+/-) | 3 | 162 | 12.7 | 18.6 | 9.3 | Aniso; Macro; Small aggr | 70 | 72 | 100 |
| F9.III.2 | F | P (+/-) | 4 | 91 | 13.4 | NA | 18.2 | Aniso; Macro; Rare giant | 79 | 86 | 119 |
| F9.III.4 | F | P (+/-) | 1 | 109 | 12.2 | 17.1 | 7.7 | Aniso; Macro | 73 | 69 | 82 |
| F9.III.5 | F | P (+/-) | 0 | 129 | 13.3 | 17.8 | 10.0 | Aniso; Macro | 77 | 71 | 94 |
| F9.II.5 | F | HR (-/-) | 0 | 246 | 9.9 | NA | 7.9 | Rare giant; Small aggr. | 95 | 97 | 88 |
| F9.III.3 | F | HR (-/-) | 0 | 306 | NA | NA | NA | NA | 79 | 86 | 93 |
| **Family 10** |  |  |  |  |  |  |  |  |  |  |  |
| F10.II.3 | M | PI (+/-) | 1 | 112 | 13.3 | 18.6 | 18.6 | Aniso; Some giant | 66 | 65.5 | 113 |
| F10.III.5 | F | P (+/-) | 0 | 144 | 12.5 | 18.4 | 14.8 | Aniso | 71 | 80 | NA |

Color code: Patient (index case) – red; Patient – blue; Healthy relative – green.

Abbreviations: Aniso, platelet anisocytosis; BS, Bleeding score; F, female; HR, healthy relative; IPF, immature platelet fraction (%); ISTH-BAT, International Society on Thrombosis and Hemostasis – Bleeding Assessment Tool; M, male; Macro, platelet macrocytosis; MPV, mean platelet volume; NA, not available; PDW, Platelet volume distribution width; P, patient; PI, patient, index case; PLT, platelets; Small aggr, small platelet aggregates.

The levels of PLT glycoproteins are expressed as percentage of normal (%n), which was calculated by dividing the MFI obtained in PLTs from the patients after staining with fluorochrome conjugated monoclonal antibodies directed against the correspondent PLT glycoprotein, by the mean MFI obtained in the PLTs of blood samples from four adult healthy individuals (blood donors) studied in parallel with each patient.

1. BS (ISTH-BAT) – Normal range: The cut-off used for an abnormal BS was ≥4 in adult males, ≥6 in adult females and ≥3 in children. This cutoff was based in a previous study in which data from 1040 normal adults and 328 children were evaluated, which showed that the normal range is 0-3 for adult males, 0-5 for adult females and 0-2 in children for both males and females (29)
2. PLT count – Normal range: 150 – 400 x 10^9^/L; MPV – Normal range: 7 – 11 fL; PDW – Normal range: 9 – 14%; IPF – Normal range: 1-7%
3. Normal range (Percentiles 5-95, 50 healthy adults/blood donors; non-published results from our Center): CD41a (GPIIb/IIIa) (85-115%); CD61 (GPIIIa) (80-120%); CD42b (GPIb) (70-130%).
