# Supplementary material for: αIIbβ3 variants in ten families with autosomal dominant macrothrombocytopenia: Expanding the mutational and clinical spectrum
Source: PLoS One. 2020 Dec 4;15(12):e0235136. doi: 10.1371/journal.pone.0235136 (PMC7717987; doi:10.1371/journal.pone.0235136)
Supplement: S4 File — (DOCX) [file pone.0235136.s004.docx]

Title**: αIIbβ3 variants in ten families with autosomal dominant macrothrombocytopenia: expanding the mutational and clinical spectrum**

Short title: **Familial macrothrombocytopenia with αIIbβ3 integrin deficiency**

**AUTHORS:** Sara Morais, Jorge Oliveira, Catarina Lau, Mónica Pereira, Marta Gonçalves, Catarina Monteiro, Ana Rita Gonçalves, Rui Matos, Marco Sampaio, Eugénia Cruz, Inês Freitas, Rosário Santos, Margarida Lima

## SUPPLEMENTARY FILE 4 (S4 FILE)

## S4 File | RESULTS: PLATELET FUNCTION AND LUMIAGGREGOMETRY ASSAYS

### S4 File | Table 1. Closure times, as evaluated by platelet function assays, and platelet aggregation/agglutination and ATP release, as evaluated by lumiaggregometry in families studied

|  |  |  | **Platelet function assays** | |  | **Platelet lumiaggregometry assays** | | | | | | | | | | | |
| --- | --- | --- | --- | --- | --- | --- | --- | --- | --- | --- | --- | --- | --- | --- | --- | --- | --- |
|  |  |  | **PFA-100** | |  | **COL** | | **ADP** | | **AA** | | **EPI** | | **TRAP-6** | | **RIST** | |
| Family | Patient |  | **Closure times** | |  | 1µg/ml | | 10µM | | 1mM | | 10µM | | 25µM | | 1mM | 0.5mM |
|  |  |  | **COL/EPI**  (seconds) | **COL/ADP**  (seconds) |  | **PAGR**  (%) | **ATP-R**  (nmol) | **PAGR**  (%) | **ATP-R**  (nmol) | **PAGR**  (%) | **ATP-R**  (nmol) | **PAGR**  (%) | **ATP-R**  (nmol) | **PAGR**  (%) | **ATP-R**  (nmol) | **PAGL**  (%) | **PAGL**  (nmol) |
| F1 | II.2 |  | 293 ↑  (300/286) | 171 ↑  (147/194) |  | 56 ↓ | 0.32 ↓ | 51 ↓ | 0.11 ↓ | 30 ↓ | 0.14 ↓ | NA | NA | 48 ↓ | 0.39 ↓ | NA | NA |
| F2 | II.1 |  | 245 ↑  (262/229) | 101  (78/123) |  | NA | NA | NA | NA | NA | NA | NA | NA | NA | NA | NA | NA |
| F3 | II.1 |  | 119  (130/108) | 126 ↑  (89/163) |  | 79 | 0.17 ↓ | 39 ↓ | 0 ↓ | 67 | 0 ↓ | 24 | 0 ↓ | 57 ↓ | 0 ↓ | 72 | 0 |
| F4 | II.1 |  | 104  (104/119/102) | 121 ↑  (96/121/145) |  | 65 | 0.67 ↓ | 50 ↓ | 0.43 ↓ | 41 ↓ | 0.29 ↓ | 42 ↓ | 0.24 ↓ | 38 ↓ | 0.52 ↓ | 72 | 0 |
| F5 | III.1 |  | 100  (84/115) | 127 ↑  (120/133) |  | 26 ↓ | 0.27 ↓ | 28 ↓ | 0 ↓ | 13 ↓ | 0 ↓ | 9 ↓ | 0 ↓ | 25 ↓ | 0.23 ↓ | 7 ↓ | 0 |
| F6 | III.1 |  | 118  (116/165/120/67) | 85  (88/135/77/81) |  | 0 ↓ | 0 ↓ | 5 ↓ | 0.19 ↓ | 0 ↓ | 0 ↓ | 12 ↓ | 0 ↓ | 4 ↓ | 0.19 ↓ | 57 | 0 |
| F7 | II.2 |  | 90  (93/87) | 73  73/72 |  | 39 ↓ | 0.27 ↓ | 20 ↓ | 0.16 ↓ | 76 | 0.20 ↓ | 8 ↓ | 0 ↓ | 16 ↓ | 0 ↓ | 83 | NA |
| F8 | II.2 |  | 101  (98/105/101) | 81  (85/81/76) |  | 59 ↓ | 0.55 ↓ | 61 | 0 ↓ | 77 | 0 ↓ | 49 ↓ | 0 ↓ | 77 | 0.27 ↓ | 68 | 0 |
| F9 | II.4 |  | 89  (99/90/77) | 98  (85/102/107) |  | 58 ↓ | 0.54 ↓ | 54 ↓ | 0.38 ↓ | 42 ↓ | 0.33 ↓ | 60 | 0.25 ↓ | 33 ↓ | 0.17 ↓ | 74 | 0 |
| F10 | III.5 |  | 125 | 90 |  | NA | NA | NA | NA | NA | NA | NA | NA | NA | NA | NA | NA |
| Normal range | |  | 88-150 | 56-120 |  | 60-112 | 0.7-2.7 | 61-100 | 0.3-2.6 | 62-105 | 0.6-2.3 | 56-112 | 0.5-2.5 | 70-113 | 0.5-2.5 | 57-111 | 0.0 |

Abbreviations: AA, arachidonic acid; ADP, Adenosine diphosphate; ATP, adenosine triphosphate; ATPR, ATP release; COL, collagen; EPI, epinephrine; NA, not available; PAGR, Platelet aggregation; PAGL, platelet agglutination; PFA, Platelet Function Assay; RIST, ristocetin; TRAP-6, Thrombin Receptor Agonist Peptide.

For simplicity, only a representative patient is presented per family, in this table.

PFA-100: COL/EPI and COL/ADP occlusion times presented in this table are the median of at least two independent determinations.

Platelet aggregation studies were performed in platelet rich plasma; results are given as percentage maximum aggregation.
