# Supplementary material for: αIIbβ3 variants in ten families with autosomal dominant macrothrombocytopenia: Expanding the mutational and clinical spectrum
Source: PLoS One. 2020 Dec 4;15(12):e0235136. doi: 10.1371/journal.pone.0235136 (PMC7717987; doi:10.1371/journal.pone.0235136)
Supplement: S5 File — (DOCX) [file pone.0235136.s005.docx]

Title**: αIIbβ3 variants in ten families with autosomal dominant macrothrombocytopenia: expanding the mutational and clinical spectrum**

Short title: **Familial macrothrombocytopenia with αIIbβ3 integrin deficiency**

**AUTHORS:** Sara Morais, Jorge Oliveira, Catarina Lau, Mónica Pereira, Marta Gonçalves, Catarina Monteiro, Ana Rita Gonçalves, Rui Matos, Marco Sampaio, Eugénia Cruz, Inês Freitas, Rosário Santos, Margarida Lima

**SUPPLEMENTARY FILE 5 (S5 FILE)**

## S5 File | RESULTS: FLOW CYTOMETRY BASED PLATELET ACTIVATION STUDIES

### S5 File | Table 1. Activation-induced binding sites on the GPIIb/IIIa receptor, as recognized by anti-PAC-1 monoclonal antibody, and receptor induced binding sites on its ligand, fibrinogen, as recognized by anti-bound fibrinogen monoclonal antibody, in inhibitory conditions, in basal conditions, and after stimulation with TRAP-6 or with ADP, in blood samples from GTLS patients and healthy controls studied in parallel

| Individual | | TRAP-6 | | | | | | |  | ADP | | | | | | |
| --- | --- | --- | --- | --- | --- | --- | --- | --- | --- | --- | --- | --- | --- | --- | --- | --- |
|  |  | AIBS (PAC-1) | | |  | RIBS (bFG, 9F9) | | |  | AIBS (PAC-1) | | |  | RIBS (bFG, 9F9) | | |
|  |  | INH  (EDTA) | BAS  (PBS) | STI  (TRAP-6)  (20 µM) |  | INH  (EDTA) | BAS  (PBS) | STI  (TRAP-6)  (20 µM) |  | INH  (EDTA) | BAS  (PBS) | STI  (ADP)  (10 µM) |  | INH  (EDTA) | BAS  (PBS) | STI  (ADP)  (20 µM) |
|  | C1 | 0.121 | 0.796 | 12.300  (92%) [76%] |  | 0.775 | 1.250 | 12.800  (105%) [131%] |  | 0.129 | 1.520 | 11.000  (89%) [77%] |  | 0.879 | 1.290 | 12.600  (103%) [111%] |
|  | C2 | 0.121 | 0.702 | 14.400  (108%) [89%] |  | 0.911 | 1.010 | 11.800  (95%) [119%] |  | 0.133 | 1.290 | 13.800  (111%) [97%] |  | 0.768 | 0.944 | 11.800  (97%) [104%] |
| F1 | III.4 | 0.180 | 0.494 | 1.690  **(11%) [9%]** |  | 1.120 | 1.270 | 2.520  **(12%) [15%]** |  | 0.163 | 0.517 | 6.590  **(52%) [46%]** |  | 1.060 | 1.120 | 5.460  **(39%) [42%]** |
|  | C3 | 0.102 | 0.280 | 16.600  (100%) [103%] |  | 0.619 | 0.576 | 11.800  (100%) [122%] |  | 0.102 | 0.268 | 13.400  (96%) [95%] |  | 0.522 | 0.576 | 12.700  (109%) [115%] |
|  | C4 | 0.102 | 0.327 | NA  (NA) [NA] |  | 0.551 | 0.561 | NA  (NA) [NA] |  | 0.102 | 0.307 | 14.400  (104%) [102%] |  | 0.517 | 0.581 | 10.600  (91%) [96%] |
| F2 | I.2 | 0.112 | 0.541 | 2.340  **(14%) [14%]** |  | 0.551 | 0.647 | 1.590  **(9%) [11%]** |  | 0.104 | 0.613 | 8.870  **(64%) [62%]** |  | 0.586 | 0.734 | 5.170  **(41%) [43%]** |
|  | C5 | 0.102 | 0.273 | 14.000  (93%) [87%] |  | 0.460 | 0.481 | 4.990  (80%) [49%] |  | 0.102 | 0.336 | 13.900  (93%) [98%] |  | 0.428 | 0.464 | 5.080  (66%) [44%] |
|  | C6 | 0.102 | 0.929 | 16.100  (107%) [100%] |  | 0.508 | 0.561 | 7.280  (120%) [74%] |  | 0.102 | 0.754 | 16.100  (107%) [114%] |  | 0.503 | 0.546 | 9.880  (134%) [89%] |
| F4 | II.1 | 0.102 | 1.010 | 7.020  **(46%) [43%]** |  | 0.613 | 0.734 | 3.880  **(58%) [36%]** |  | 0.102 | 0.818 | 8.250  **(55%) [58%]** |  | 0.576 | 0.677 | 7.890  **(104%) [69%]** |
|  | C7 | 0.151 | 0.796 | 16.500  (102%) [102%] |  | 0.683 | 0.671 | 5.510  (86%) [53%] |  | 0.247 | 0.456 | 15.900  (108%) [111%] |  | 0.624 | 0.636 | 8.250  (109%) [72%] |
|  | C8 | 0.158 | 0.503 | 15.900  (98%) [98%] |  | 0.741 | 0.754 | 7.080  (114%) [69%] |  | 0.151 | 0.283 | 13.400  (92%) [94%] |  | 0.782 | 0.803 | 7.210  (91%) [61%] |
| F5 | II.1 | 0.180 | 0.586 | 5.360  **(32%) [32%]** |  | 0.818 | 0.911 | 6.450  **(101%) [61%]** |  | 0.168 | 0.499 | 9.880  **(67%) [69%]** |  | 0.833 | 0.928 | 5.170  **(62%) [41%]** |
| F5 | III.1 | 0.179 | 0.928 | 8.250  **(50%) [50%]** |  | 0.789 | 0.936 | 3.070  **(41%) [25%]** |  | 0.163 | 0.659 | 12.000  **(82%) [84%]** |  | 0.810 | 0.970 | 6.650  **(83%) [55%]** |
|  | C9 | 0.102 | 2.430 | 18.700  (88%) [116%] |  | 0.503 | 1.050 | 16.900  (101%) [179%] |  | 0.102 | 1.800 | 15.800  (84%) [112%] |  | 0.531 | 0.741 | 15.300  (89%) [140%] |
|  | C10 | 0.121 | 4.640 | 23.600  (112%) [147%] |  | 0.659 | 1.580 | 16.600  (99%) [174%] |  | 0.107 | 4.850 | 22.000  (116%) [156%] |  | 0.653 | 1.260 | 19.000  (111%) [174%] |
| F6 | III.2 | 0.125 | 0.597 | 1.350  **(6%) [8%]** |  | 0.494 | 0.517 | 0.653  **(1%) [2%]** |  | 0.120 | 0.581 | 4.240  **(22%) [29%]** |  | 0.512 | 0.531 | 1.550  **(6%) [10%]** |
|  | C9 | 0.102 | 2.430 | 18.700  (88%) [116%] |  | 0.503 | 1.050 | 16.900  (101%) [179%] |  | 0.102 | 1.800 | 15.800  (84%) [112%] |  | 0.531 | 0.741 | 15.300  (89%) [140%] |
|  | C10 | 0.121 | 4.640 | 23.600  (112%) [147%] |  | 0.659 | 1.580 | 16.600  (99%) [174%] |  | 0.107 | 4.850 | 22.000  (116%) [156%] |  | 0.653 | 1.260 | 19.000  (111%) [174%] |
| F7 | II.2 | 0.165 | 1.270 | 5.920  **(27%) [36%]** |  | 0.647 | 0.683 | 2.410  **(11%) [19%]** |  | 0.148 | 1.110 | 8.030  **(42%) [46%]** |  | 0.665 | 0.768 | 4.940  **(26%) [40%]** |
|  | C3 | 0.102 | 0.280 | 16.600  (100%) [103%] |  | 0.619 | 0.576 | 11.800  (100%) [122%] |  | 0.102 | 0.268 | 13.400  (96%) [95%] |  | 0.522 | 0.576 | 12.700  (109%) [115%] |
|  | C4 | 0.102 | 0.327 | NA  (NA) [NA] |  | 0.551 | 0.561 | NA  (NA) [NA] |  | 0.102 | 0.307 | 14.400  (104%) [102%] |  | 0.517 | 0.581 | 10.600  (91%) [96%] |
| F8 | II.2 | 0.105 | **2.300** | 11.200  **(67%) [69%]** |  | 0.541 | 0.689 | 6.710  **(55%) [67%]** |  | 0.102 | **2.240** | 13.400  **(96%) [95%]** |  | 0.546 | 0.734 | 14.400  **(124%) [131%]** |
|  | C5 | 0.102 | 0.273 | 14.000  (93%) [87%] |  | 0.460 | 0.481 | 4.990  (80%) [49%] |  | 0.102 | 0.336 | 13.900  (93%) [98%] |  | 0.428 | 0.464 | 5.080  (66%) [44%] |
|  | C6 | 0.102 | 0.929 | 16.100  (107%) [100%] |  | 0.508 | 0.561 | 7.280  (120%) [74%] |  | 0.102 | 0.754 | 16.100  (107%) [114%] |  | 0.503 | 0.546 | 9.880  (134%) [89%] |
| F9 | III.2 | 0.108 | **1.520** | 4.090  **(27%) [25%]** |  | 0.619 | 0.641 | 2.660  **(36%) [22%]** |  | 0.102 | **1.370** | 9.030  **(60%) [64%]** |  | 0.597 | 0.677 | 9.110  **(121%) [81%]** |
|  | C11 | 0.101 | 0.988 | 19.600  (120%) [122%] |  | 0.485 | 0.561 | 9.135  (100%) [57%] |  | 0.102 | 0.754 | 17.700  (117%) [125%] |  | 0.472 | 0.555 | 15.100  (128%) [139%] |
|  | C12 | 0.102 | 0.780 | 13.200  (80%) [82%] |  | 0.517 | 0.546 | 9.183  (100%) [57%] |  | 0.102 | 0.796 | 12.700  (83%) [90%] |  | 0.405 | 0.404 | 8.650  (72%) [78%] |

Abbreviations: ADP, adenosine diphosphate; AIBS, activation-induced binding sites on GPIIb/IIIa; BAS, basal state; C, control; EDTA, ethylene-diamine-tetracetic acid; F, family; INH, inhibitory state; RIBS, receptor induced binding sites on fibrinogen bound to GPIIb/IIIa; STI, stimulated state; TRAP-6, Thrombin Receptor Agonist Peptide 6; Final concentrations: EDTA-K3 (7 mM); ADP (10 µM); TRAP-6 (20 µM).

Results from the GTLS patients are preceded by the results obtained in the healthy controls studied in parallel. No patients from F3 and F10 were studied.

The results are expressed as median fluorescence intensity (MFI, arbitrary units). Values indicated between curved parenthesis (…) were calculated as a function of the 2 normal samples of the day, processed in parallel with the patient samples; values ​​indicated between square brackets […] were calculated as a function of the 12 normal samples evaluated.

### S5 File | Table 2. Basal and agonist-induced platelet activation status as determined by flow cytometry in healthy controls and in representative patients from the GTLS families, as evaluated by the platelet activation index and percentages of the normal values.

|  | Platelet activation indexes (PAI) | | | | | | | | | | |  | % normal values | | | | | | |
| --- | --- | --- | --- | --- | --- | --- | --- | --- | --- | --- | --- | --- | --- | --- | --- | --- | --- | --- | --- |
|  | TRAP-6 experiments | | | | |  | ADP experiments | | | | |  | TRAP-6 experiments | | |  | ADP experiments | | |
|  | AIBS (PAC-1) | |  | RIBS (bFG) | |  | AIBS (PAC-1) | |  | RIBS (bFG) | |  | AIBS (PAC-1) |  | RIBS (bFG) |  | AIBS (PAC-1) |  | RIBS (bFG) |
|  | Bas | Stim |  | Bas | Stim |  | Bas | Stim |  | Bas | Stim |  | Stim |  | Stim |  | Stim |  | Stim |
| HEALTHY CONTROLS (n=12) | | | | | | | | | | | |  |  |  |  |  |  |  |  |
| C1 | 6x | 101x |  | 1x | 16x |  | 11x | 84x |  | 1x | 13x |  | 76% |  | 131% |  | 77% |  | 111% |
| C2 | 5x | 118x |  | 0x | 12x |  | 9x | 103x |  | 0x | 14x |  | 89% |  | 119% |  | 97% |  | 105% |
| C3 | 2x | 162x |  | 0x | 18x |  | 2x | 130x |  | 0x | 23x |  | 103% |  | 122% |  | 95% |  | 115% |
| C4 | 2x | NA |  | 0x | NA |  | 2x | 140x |  | 0x | 20x |  | NA |  | NA |  | 102% |  | 96% |
| C5 | 2x | 136x |  | 0x | 10x |  | 2x | 135x |  | 0x | 11x |  | 87% |  | 49% |  | 98% |  | 44% |
| C6 | 8x | 157x |  | 0x | 13x |  | 6x | 157x |  | 0x | 19x |  | 100% |  | 74% |  | 114% |  | 89% |
| C7 | 4x | 108x |  | 0x | 7x |  | 1x | 63x |  | 0x | 12x |  | 102% |  | 53% |  | 111% |  | 72% |
| C8 | 2x | 100x |  | 0x | 9x |  | 1x | 88x |  | 0x | 8x |  | 98% |  | 69% |  | 94% |  | 61% |
| C9 | **23x** | 182x |  | 1x | 33x |  | **17x** | 154x |  | 0x | 28x |  | 116% |  | 179% |  | 112% |  | 140% |
| C10 | **37x** | 194x |  | 1x | 24x |  | **44x** | 205x |  | 1x | 28x |  | 147% |  | 174% |  | 156% |  | 174% |
| C11 | 9x | 193x |  | 0x | 19x |  | 6x | 173x |  | 0x | 31x |  | 122% |  | 100% |  | 125% |  | 139% |
| C12 | 7x | 128x |  | 0x | 18x |  | 7x | 124x |  | 0x | 15x |  | 82% |  | 100% |  | 90% |  | 59% |
| GT PATIENTS (n=1) |  |  |  |  |  |  |  |  |  |  |  |  |  |  |  |  |  |  |  |
| GT patient | 0x | 0x |  | 0x | 1x |  | 0x | 0x |  | 0x | 0x |  | 0% |  | 0% |  | 0% |  | 0% |
| GTLS PATIENTS (n=9) | | | | | | | | | | | |  |  |  |  |  |  |  |  |
| F1.III.4 | 2x | 8x |  | 0x | 1x |  | 2x | 39x |  | 0x | 4x |  | 9% |  | 15% |  | 46% |  | 42% |
| F2.I.2 | 4x | 20x |  | 0x | 2x |  | 5x | 84x |  | 0x | 8x |  | 14% |  | 11% |  | 62% |  | 43% |
| F3 | NA | NA |  | NA | NA |  | NA | NA |  | NA | NA |  | NA |  | NA |  | NA |  | NA |
| F4.II.1 | 9x | 68x |  | 0x | 5x |  | 7x | 80x |  | 0x | 13x |  | 43% |  | 36% |  | 58% |  | 69% |
| F5.II.1 | 2x | 29x |  | 0x | 7x |  | 2x | 58x |  | 0x | 5x |  | 32% |  | 61% |  | 69% |  | 41% |
| F5.I.1 | 4x | 45x |  | 0x | 3x |  | 3x | 73x |  | 0x | 7x |  | 51% |  | 25% |  | 84% |  | 55% |
| F6.III.2 | 4x | 10x |  | 0x | 0x |  | 4x | 34x |  | 0x | 2x |  | 8% |  | 2% |  | 29% |  | 10% |
| F7.II.2 | 7x | 35x |  | 0x | 3x |  | 7x | 53x |  | 0x | 6x |  | 36% |  | 19% |  | 56% |  | 41% |
| F8.II.2 | **21x** | 106x |  | 0x | 11x |  | **21x** | 130x |  | 0x | 25x |  | 69% |  | 67% |  | 95% |  | 131% |
| F9.III.2 | **13x** | 37x |  | 0x | 3x |  | **12x** | 88x |  | 0x | 14x |  | 27% |  | 22% |  | 64% |  | 81% |
| F10 | NA | NA |  | NA | NA |  | NA | NA |  | NA | NA |  | NA |  | NA |  | NA |  | NA |

Abbreviations: ADP, adenosine diphosphate; AIBS, activation-induced binding sites on the GPIIb/IIIa receptor; BAS, basal conditions, unstimulated (PBS, no agonist); bFG, bound fibrinogen; C, control; FI, fold increase; GT, Glanzmann Thrombasthenia; GTLS, Glanzmann Thrombasthenia like syndrome; NA, not available; PAI, platelet activation index; PBS, phosphate buffered saline; RIBS, receptor-induced binding sites on the fibrinogen ligand; STIM, stimulated; TRAP-6, Thrombin Receptor Agonist Peptide-6,
